# Supplementary material for: Anal human papillomavirus infection among men who have sex with men and transgender women living with and without HIV in Pakistan: findings from a cross-sectional study
Source: BMJ Open. 2021 Nov 1;11(11):e052176. doi: 10.1136/bmjopen-2021-052176 (PMC8562516; doi:10.1136/bmjopen-2021-052176)
Supplement: Author's manuscript [file bmjopen-2021-052176.draft_revisions.pdf]

BMJ Open is committed to open peer review. As part of this commitment we make the peer review history of every article we publish publicly available.

When an article is published we post the peer reviewers' comments and the authors' responses online. We also post the versions of the paper that were used during peer review. These are the versions that the peer review comments apply to.

The versions of the paper that follow are the versions that were submitted during the peer review process. They are not the versions of record or the final published versions. They should not be cited or distributed as the published version of this manuscript.

BMJ Open is an open access journal and the full, final, typeset and author-corrected version of record of the manuscript is available on our site with no access controls, subscription charges or pay-per-view fees (http://bmjopen.bmj.com).

If you have any questions on BMJ Open's open peer review process please email info.bmjopen@bmj.com

BMJ Open

Type-specific prevalence of anal human papillomavirus infection among men who have sex with men and transgender women living with and without HIV in Karachi Pakistan: Implications for prevention strategies

Journal:	*BMJ Open*
Manuscript ID	bmjopen-2021-052176
Article Type:	Original research
Date Submitted by the Author:	07-Apr-2021
Complete List of Authors:	Ejaz, Muslima; Aga Khan University Medical College Pakistan, Community Health Sciences; Karolinska Institutet, Public Health Department<br>Andersson, Soren; Orebro Universitet, Lab Medicine<br>Batool, Salma; Sindh Institute of Urology and Transplantation, Molecular Biology<br>Ali, Tazeen; Aga Khan University Medical College Pakistan, Community Health Sciences & School of Nursing<br>Ekström, Anna; Karolinska Institutet, Department of Public Health Sciences
Keywords:	Epidemiology < INFECTIOUS DISEASES, HIV & AIDS < INFECTIOUS DISEASES, MOLECULAR BIOLOGY, PUBLIC HEALTH, SEXUAL MEDICINE

SCHOLARONE™
Manuscripts

I, the Submitting Author has the right to grant and does grant on behalf of all authors of the Work (as defined in the below author licence), an exclusive licence and/or a non-exclusive licence for contributions from authors who are: i) UK Crown employees; ii) where BMJ has agreed a CC-BY licence shall apply, and/or iii) in accordance with the terms applicable for US Federal Government officers or employees acting as part of their official duties; on a worldwide, perpetual, irrevocable, royalty-free basis to BMJ Publishing Group Ltd ("BMJ") its licensees and where the relevant Journal is co-owned by BMJ to the co-owners of the Journal, to publish the Work in this journal and any other BMJ products and to exploit all rights, as set out in our licence.

The Submitting Author accepts and understands that any supply made under these terms is made by BMJ to the Submitting Author unless you are acting as an employee on behalf of your employer or a postgraduate student of an affiliated institution which is paying any applicable article publishing charge ("APC") for Open Access articles. Where the Submitting Author wishes to make the Work available on an Open Access basis (and intends to pay the relevant APC), the terms of reuse of such Open Access shall be governed by a Creative Commons licence – details of these licences and which Creative Commons licence will apply to this Work are set out in our licence referred to above.

Other than as permitted in any relevant BMJ Author's Self Archiving Policies, I confirm this Work has not been accepted for publication elsewhere, is not being considered for publication elsewhere and does not duplicate material already published. I confirm all authors consent to publication of this Work and authorise the granting of this licence.

For peer review only

Type-specific prevalence of anal human papillomavirus infection among men who have sex with men and transgender women living with and without HIV in Karachi Pakistan: Implications for prevention strategies

Muslima Ejaz, MD MSc[1,2] Sören Andersson MD, PhD[3], Salma Batool PhD[4], Tazeen Saeed Ali PhD[5], Anna Mia Ekström MD MPH PhD[1]

[1]Department of Global Public Health, Karolinska Institutet Stockholm Sweden

[2] Department of Community Health Sciences the Aga Khan University Karachi Pakistan

[3] Department of Laboratory Medicine, Faculty of Medicine and Health, Örebro University, Örebro, Sweden

[4] Department Molecular Biology, Sindh Institute of Urology and Transplantation Karachi Pakistan

[5] School of Nursing, The Aga Khan University Karachi Pakistan

*Corresponding author

Dr. Muslima Ejaz

Doctoral candidate at Department of Global Public Health

Global & Sexual Health Research group (GloSH)

Karolinska Institutet

Stockholm Sweden

Muslima.ejaz@ki.se

Senior Instructor at Department of Community Health Sciences

Aga Khan University (AKU)

Stadium Road, P.O. Box 3500

Karachi 74800, Pakistan

Phone: 92-21-34864886

Muslima.ejaz@aku.edu

For peer review only

For peer review only - http://bmjopen.bmj.com/site/about/guidelines.xhtml

ABSTRACT

Objectives: Anal infection with Human Papillomavirus (HPV) is very common worldwide among men having sex with men (MSM) and in particular among MSM living–with-HIV**.** The high prevalence of HIV among MSM and male-to-female transgendered individuals in Pakistan is a significant health concern since access-to-screening and health-seeking often is delayed in this stigmatized key population. Their participation in commercial sex-work also makes this a public health concern at larger population level. The aim of this study was to determine the prevalence of infection, genotypes and risk factors for HPV among MSM and transgender-women living with-and-without HIV in Karachi, Pakistan.

Design: This cross-sectional study was conducted between March-2016-to-November-2017.

Participants, setting and data-collection: This study recruited MSM and transgender-women who self-reported to have had anal-sex in the last 6-months and be at least 18-years of age, from the sexual-health and anti-retroviral-therapy centers. Structured questionnaires were administered and blood-samples were obtained to confirm HIV-status. Anal-swabs were collected for HPV-DNA detection and typing.

Main outcome measures: The primary outcome was the prevalence of HPV-DNA infection. The prevalence-ratios (PR) were calculated using Cox-proportional-hazard-model algorithms to analyze the association between exposure variables and HPV-infection.

Results: Complete data were available for 298 MSM and transgender-women (HIV+ n=131; HIV- n=167). The overall HPV-DNA prevalence was 65.1%, higher in participants living-with-HIV as compared to HIV-negative (87%versus48%; p=<0.001). The most frequent HPV-types were HPV6/11(46.9%), followed by HPV16(35.1%), HPV18(23.2%), HPV35(21.1%), HIV-status(PR: 1.81 95% CI 1.16-2.82), and never-condom-use (PR:2.31, 95%CI(1.03-5.20)) were independently associated with prevalence of any-type anal-HPV-infection, when adjusting for confounding.

Conclusion: High prevalence of HPV indicates a substantial future risk of anal-cancer in Pakistani MSM and transgender-women, and in those living-with-HIV in particular. Current findings support HPV vaccination efforts to this high-risk population. Dedicated efforts are needed to make those services available for both public health and human rights purposes.

For peer review only

For peer review only - http://bmjopen.bmj.com/site/about/guidelines.xhtml

Strengths and limitations of this study

- This is the first-ever conducted study in extremely conservative heteronormative society
- Reaching this population and gaining their trust was challenging, as they prefer to live secretive lives due to continuous targets for ridicule and physical assault in an overtly hostile environment.
- HPV high oncogenic risk type 35 which is not included in 9-valent vaccine was more common in Pakistan compared to elsewhere with the exception of India with implications on anal cancer prevention in the South Asian region
- The cross sectional nature of the study preclude us to built any temporal relationships with the potential risk factors
- We cannot exclude the possibility of under- or over-reporting of risky sexual behaviors and lifestyle factors that might also lead to information bias since they were self-reported.
-

review only

For peer review only - http://bmjopen.bmj.com/site/about/guidelines.xhtml

INTRODUCTION:

Human papillomaviruses (HPV) is the most common sexually transmitted infection (STI) worldwide [1], and rapidly acquired after sexual debut[2]. A meta-analysis of global data on HPV in MSM found prevalence rates of anal HPV to be 93% in MSM living with HIV and 64% in HIV-negative MSM, and these rates did not decrease with increasing age[3]. More than 90 genotypes of HPV have been identified and classified either as low risk (LR) types causing genital warts (predominantly 6 and 11) or high risk (HR) types which can cause cancer of the cervix, anus and oropharynx (predominantly 16 and 18). Whilst the burden of HPV-related disease in all men is lower than in all women, certain at risk groups such as MSM and transgender women, and particularly those with HIV infection, are at high risk for HPV infection[4], and related anal cancer and its precursor high-grade squamous intraepithelial lesions[5]. The reported incidence of anal cancer is 1 – 2/100,000 in general population[6], while in MSM it has been reported up to 35 cases per 100,000[7] and even up to 131/100,000, in MSM living with HIV[8,9] regardless if they are on antiretroviral treatment or not [10-12].

In Pakistan the demography of MSM is broadly classified as; ***MSM*** who are in relationships but outwardly closeted, as Male Sex Workers (***MSW)*** who are males and undertakes receptive anal or oral sexual activity with a man in return of money or other financial benefits, and as ***hijras*** (transvestites/transgender women) who are biologically male with male genitals but function in society as if female and take the receptive role in anal sex [13,14]. The "hijra" is a South-Asian identity traced back many centuries to India, where they were held in high-esteem and were political advisors to kings and bodyguards to queens. Today, however, they often face social exclusion and many engage in commercial sex work[15].

According to the survey conducted for mapping of key populations in 2015-16, the estimated number of MSM in 23 cities of Pakistan was 46,264 (range: 39,273 – 53,257) who were congregated at 8,606 geographical spots. Karachi reported the largest spot sizes and the estimated number of MSM was 18,361, mapped at 3,495 spots. Estimates of MSM from Karachi alone made an approximately 35% of the total estimates calculated [16]. Pakistan's national government has lately recognized MSM as a vulnerable population, and prevention and control programs targeting MSMs in its big cities are being organized. However, program service delivery, such as HIV counseling and testing and other STI screening services is performed by non-governmental and community based organizations. , They are dependent on national and international funders, this has led to fragmented and incomplete coverage of preventive services for this key population. [17,18]

For peer review only - http://bmjopen.bmj.com/site/about/guidelines.xhtml

Pakistan is an authoritarian Islamic republic with conservative social values, particularly on issues of sex. MSM, and in particular MSW, face high levels of discrimination and contempt from society and are subject to harassment, sexual violence and detention by the state authorities. Their health-seeking behaviors are often poor due to stigmatization in healthcare settings and for the fear of being identified. As a result they are reluctant to seek health care not only in public set-up but also in private facilities. One previous study on sex work and MSW in Pakistan, showed that the majority of MSWs are poor, have little knowledge about safe sex, and cannot negotiate the use of condoms and safe sex with their paying clients, predisposing them to acquire STIs[14].

The prevalence of HIV in these groups is reaching alarming proportions. Results from the 5th round of Integrated Behavior and Biological second-generation surveillance for HIV/AIDS in Pakistan 2016-17 reported over 9% HIV prevalence in MSM and almost 13% in Transgender women living in Karachi[16].

So far there is no research on the burden of HPV infection and HPV-related anal squamous intraepithelial lesions in this key population in Pakistan. Furthermore, the bivalent vaccine protects against HPV16/18 and quadrivalent vaccine protects against HPV16/18 and the low-risk types HPV6/11[19]. Currently, HPV vaccine (bi and quadrivalent) is available with pharmacies in Pakistan but is not yet made available to the target population. The Ministry of Health has not yet included it in their National immunization program for any group in society.

We conducted a cross sectional study in Karachi Pakistan in 2016-17 with the objective to determine the prevalence and risk factors for anal HPV infection including sexual risk behaviors and the association with HIV infection in this dynamic population to inform future policy and prevention strategies.

METHODS

Setting and participants:

Enrollment for this cross-sectional study occurred from March 2016 to November 2017. MSM and transgender women were eligible for the study who self-reported to have had anal sex in the last preceding at least 6 months and of age 18 and above. On their pre-scheduled ART collection date study participation was offered to consecutive MSM and TG women living with HIV in a public sector antiretroviral treatment for HIV (ART) center run by the National AIDS Control Program of Pakistan in Civil Hospital Karachi (CHK) - the largest (1,900 bed) tertiary care public hospital of Karachi Pakistan. Study participation was also offered to MSM and transgender women coming to a sexual health clinic run by a community-based organization, Perwaaz Trust Karachi Pakistan. Here the study population was also reached using snowball technique and peer referrals.

For peer review only - http://bmjopen.bmj.com/site/about/guidelines.xhtml

Patient and public Involvement: No patients or member of the public were involved in the design, or conduct, or reporting, or dissemination plans of our study. Verbal informed consent was obtained from all study participants. Data were kept confidential and anonymous and was used for research purpose only.

Study Procedure: The Ethics Committee on Human Research of Aga Khan (3612-CHS-ERC-15) and Dow (IRB-557/DUHS/APPROVAL/2015/84) Universities of Health Sciences approved the study. Informed consent was obtained for *all study procedures*. Participants were informed that they were free to interrupt the interview or withdraw from the study at any time without any negative consequences in terms of clinical services provided to them. A 35-40 minutes long structured questionnaire was administered at a private space to interview the study participants to collect data concerning socio-demographic information, sexual and reproductive history and medical history relevant to HIV, any anal disease and use of ART. A blood sample was obtained for confirmation of their HIV status (through Architect HIV Ag-Ab Combo kit by ABBOTT), viral load (Real time PCR Artus Qiagen) and CD4+ T-cell count (through FACS Count TM flow cytometry, Becton Dickinson Co., Franklin Lakes, NJ, USA). Two anal swabs for HPV testing were collected by a trained general physician. A water-moistened swab was inserted 5 – 6 cm proximal to the anal verge and the samples were obtained from anorectal transition zone around the dentate line in the anal canal. The swab was then agitated vigorously in a tube containing 3 mL of methanol-based fixative - a Sample Transport Medium (UTM-RT viral transport media with flocked polyester swabs, Copan Diagnostics Inc., Corona, CA) and transported to the lab. In Molecular lab the solution was stored at -70 °C before further processing and was later used for the extraction and detection of HPV DNA by the polymerase chain reaction (PCR). The DNA from rectal swab was extracted by using Qiagen DNA mini kit and DNA was eluted in 80 ul of elution buffer and stored at -80 °C for further use. HPV DNA was detected by using MY09/MY11 primers from L1 region resulted in amplification of 450 bp of L1 gene. PCR for Housekeeping gene Glyceraldehyde 3-phosphate dehydrogenase (GAPDH) was done for every extracted DNA and samples tested negative for GAPDH gene were excluded from study. Nested Multiplex PCR (NMPCR) assay was used [20] for detection HPV subtypes included low risk types 16/11 and high-risk (group 1) types 16, 18, 31, 33, 35,52,56,58 and 59 as described by Sotlar et al[21]. Primers for first-round PCR for GP-E6/E7 consensus sequence and second round PCR for HPV subtypes 16, 18, 16/11 31, 33, 35,52,56,58 and 59 were synthesized from Eurofins MWG/Operon Germany. The HPV subtype specific primers were used in four cocktails and size of the nested PCR product was used for the identification of each HPV type by gel electrophoresis[22].

For peer review only - http://bmjopen.bmj.com/site/about/guidelines.xhtml

Statistical Analysis

For the estimation of HPV prevalence, we had an estimated sample size of 267 MSM with 6% precision and 5% level of significance. An additional sample of 20% was added to cater for inadequate biological samples. For the analysis of factors associated with HPV infection**,** we aimed for a sample of at least 293 MSM to achieve 80% power with an anticipated prevalence ratio of 2.5 or more between HPV positive and negative, and 5% level of significance. The database was analyzed using Statistical Package for Social Sciences (IBM SPSS Statistics for Windows, Version 24.0. Armonk, NY: IBM Corp). The mean value was calculated with standard deviation for normally distributed variables, while the median with interquartile ranges was used for non-parametric quantitative variables. For categorical variables proportions were employed and for comparison of proportions between groups $\chi 2$ test was used. For association between independent risk factors and the outcome variables "any anal HPV infection" i.e., all those HPV types that we identified in our study sample and "anal HPV **16** infection". A Cox proportion hazard model algorithm was used to estimate crude and adjusted prevalence ratios (PRs) and their 95% confidence intervals (CIs) in univariate and multivariate models. A significance level of p <0.05 was used for the final model.

Patient and public Involvement:

Patients or the public were not involved in the design, or conduct, or reporting, or dissemination plans of our study. Informed consent was obtained for all study procedures.

RESULTS:

Study Population Characteristics: The final analysis included 298 MSM and transgender women (235 MSM and 63 were transgender) to assess the prevalence of HPV DNA in the anal canal. Out of these 298, 131 (44%) had an HIV-infection and 167 (56%) were HIV-negative. Among MSM living with HIV, 33 (25%) were transgender women. Almost 90% of the participants living with HIV were on ART and two third of them had undetectable HIV viral load.

The overall mean age (±SD) of the 298 recruited participants was 28.8 (8.06). The level of education was low, 28% reported no formal education and 12% less than primary level. Most respondents were currently not married. Almost 80% described their sexual identity as homosexual and were sex workers with 12.4 years mean duration of sex work. The mean age at sexual debut was 16.4 years. Only 24.2% reported consistent condom use during sex (Table 1).

For peer review only - http://bmjopen.bmj.com/site/about/guidelines.xhtml

MSM with any anal HPV infection (n=194) were younger than MSM without detectable HPV infection (28.9 versus 30.2; p = 0.027). Smoking was more prevalent among HPV-positive MSM (82.5% versus 69.2%) than among those uninfected, with a significant difference in mean age (±SD) of 15.6 (4.5) at smoking initiation. The mean age (±SD) at sexual debut among study participants with an HPV infection was 15.7 (4.3) versus 17.6 (5.2) (p= 0.001). HPV-infected had more sexual partners, more inconsistent condom users and more often practiced receptive anal sex. (Table 1).

MSM living with HIV were however older than MSM who were HIV negative (mean (SD): 30.9 (0.70) versus 27.2(0.59) p=<0.001), more frequently smokers (p=0.01), younger at initiation of smoking (p=<0.001) and had smoked for a longer duration of time (p=<0.001). They also reported younger age at sexual debut (p=<0.001), had more sexual clients (p=<0.001), were mainly the receptive anal partner (p=<0.001), and used condoms less consistently than their HIV negative counterparts (p=<0.001).

HPV DNA Prevalence and Genotype Distribution

Overall, 65.1% (n=194) of the total study population was positive for HPV DNA (95% CI: 59.5-70.3%). MSM living with HIV had a significantly higher prevalence of HPV (87% (80.0-91.0%)) compared to those who did not have HIV (48% (40.0-55.0%)) as well as any oncogenic anal HPV infection (56.4 % (49.2-63.0%). The most common HPV types were HPV 6/11 (46.9%), HPV 16 (35.1%), HPV 18 (23.2%), HPV 35 (21.1%), and HPV 58 (8.2%) (Table 2) (Figure 1)

Sexual orientation of the study sample:

Among participants living with HIV, 45.1% reported their sexual orientation as homosexual, 29.8% as bisexuals, and 25.1% as transgender-women. The sexual orientations reported by HIV uninfected participants were, 64.7% as homosexuals, 17.3% as bisexuals and 18.0 % as transgender-women. (Figure 2)

HPV prevalence by HIV status and sexual orientation:

For participants living with HIV**,** the prevalence of any type of HPV infection were 88.1 % for homosexual men, 76.9% for bisexual men and 96.9% for transgender-women, and the prevalence for any high-risk HPV infection was 74.5%, 51.3% and 81.8% respectively (p=<0.001) (Table 3, figure 3)

For HIV uninfected participants, the prevalence of any type of HPV infection was 50.9 % for homosexual men, 31.0% for bisexual men and 53.4% for transgender-women, and the prevalence for any high-risk HPV infection was 37.1%, 20.7% and 43.3% respectively (p=0.001)

For peer review only - http://bmjopen.bmj.com/site/about/guidelines.xhtml

Independent variables associated with anal HPV infection

Being HIV positive (PR 1.81 (95% CI 1.16-2.82), and inconsistent/never condom use (PR 1.18 (1.07-4.44) / (PR 2.31 (1.03-5.20) were independent risk factors associated with increased prevalence of HPV infection, adjusted for all other demographic, sexual and behavioral risk factors (Table 4).

DISCUSSION

This is the first ever report on anal HPV infection among MSM living with and without HIV in Pakistan. The high prevalence of anal HPV among MSM living with HIV (87%) in Pakistan is similar to Thailand (85.0%)[23] and China 81% - 82% [24,25]l, slightly lower than in neighboring India (95%)[26] and Canada (98%)[27], but significantly higher than what has reported from The Netherlands (65%) [28]. In HIV-negative MSM however, studies previously carried out in the United States and Latin America showed similar prevalence levels of HPV prevalence ranging from 42% to 66% which is comparable to our 48% [29-31]. In Pakistan there is heterogeneity among MSMs and it is predominantly MSWs who register with the community based organizations - with a hope to get the free medical services of any kind in a secluded setting - from where we had to recruit our study participants. It is MSWs who accounted for 80% of our study population, partly explaining the differences in the prevalence of anal HPV infection between our study and others. In a study from India, however, with similar social and cultural values Hernandez et al [26] noticed HPV prevalence of 98% in MSM living with HIV and commented on the fluid nature of sexual identity without mentioning the exact prevalence of MSWs among them. Moreover, given the fact that our study has a cross-sectional design, incubation time for infection and variation in viral clearance could be other possible explanations for the differences in the prevalence.[23,32]

For the distribution of anal HPV types our study results are similar to the studies done elsewhere[33]. HPV 16 (35.1%) was the most common anal oncogenic HPV type detected both in MSM living with and without HIV (44.7% versus 21.2%, p <0.001). This is in close approximation of the summary prevalence for HPV 16 calculated from a recent meta-analysis [3] and by others [23,27,34,35]. HPV 16 has the ability to persist in the anal canal more than any other types and also tends to cause AIN [23,27].

The prevalence of HR-HPV type 35 (21.1%) in our study was similar to the levels reported India (20%)[26], but higher than HPV type 35 levels reported from other parts of the world [31-33]. Given the fact that HPV 35 is not included in the FDA-approved 9-valent vaccine its relatively high prevalence can have implications on anal cancer prevention in our region.

For peer review only - http://bmjopen.bmj.com/site/about/guidelines.xhtml

Similar to previous research [36] our results also revealed that almost 4 out of 5 MSM carried at least one of the high-risk genotypes in their anal canal (80% MSM living with HIV versus 73% of MSM without HIV infection). This is of concern in HIV-negative MSM in particular, since infection with HR HPV type is reported to increase the likelihood of acquiring HIV[37,38]. Thus, our results indicate a high risk of development of malignant disease related to HPV infection among MSM in Pakistan.

Our study detected a high prevalence of multiple HPV types - i.e., at least 2 types, (either low-risk and high risk or high-risk & high-risk) - of 47% in HPV positive MSM as a group which is similar to other studies [39]. This indeed is a matter of concern as anal infection with multiple HPV types has been associated with AIN and progression to higher grade AIN over time[3]. Moreover, its prevalence was significantly higher in MSM living with HIV. Higher HPV reinfection rates and lower clearance rates in individuals living with HIV may also play a crucial role in the development and the progression of anal neoplastic disease[23,40].

The results of our study suggest that the most frequently distributed HPV types among Pakistani MSM could be covered by the currently available nonavalent (93.3%) and quadravalent (84%) vaccines which means that most of HPV-related diseases including cancers and anogenital warts may potentially be preventable if offered to MSM at initiation of sexual debut. However, the 20% carrying HPV 35 would not be covered by the currently available vaccines.

More than 50% of HPV-positive MSM were young, below 25 years of age, most were single and sexually very active, most to make a living and to support their families through sex work[41]. As many as 80% of HPV-positive MSM had initiated sex work in their teens, which is in line with other studies[42]. The high number of parallel sexual partners may contribute to further spread of HPV and other STIs among MSM.

Among other sexual behaviors, inconsistent or never use of condom was the only variable that was significantly associated with any HPV, or specifically HPV 16 infection. For many in our study population, negotiating safe sex is not an option, as, on an average, a male sex worker will service 3 to 5 clients per night with one-time service charge as low as 100 – 150 Pakistani rupees (US$ 0.7 to 1.03).[13 43]. Thus, financial barriers may impede consistency in condom use and oftentimes higher rates may be charged for unprotected sex. In addition, receptive anal sex was significantly more common among HPV-infected MSM, considering the fact that this is the strong transmission route and posed high risk for HIV acquisition[44]. Since the prevalence of anal HPV was more than twice as common in MSM living with HIV, this finding indeed turned out to be consistent with our multiple regression model that identified HIV infection as an independent risk factor for any anal HPV or HPV 16 infection.

For peer review only - http://bmjopen.bmj.com/site/about/guidelines.xhtml

Our study had some limitations. First, the design of the study was cross-sectional, yet many HPV infections are transient in nature [45]. Moreover, the cross-sectional design precludes us to determine a temporal relationship, however the risk factors identified in our study appear robust and have also been found in studies from other countries. Second, our study results may have limited generalizability because participants were recruited from only one city of Pakistan that is Karachi and were recruited using peer referrals and snowball sampling techniques. Nevertheless, Karachi is the largest metropolitan city of Pakistan that represents all ethnicities of the country. Moreover, in country like Pakistan which is by and large heteronormative society and where the homophobia due to cultural and religious inhibitions prevailed against this sexual minority, using above mentioned sampling technique was the most feasible option for the recruitment process. Third, we cannot exclude the possibility of under- or overreporting of sexual behaviors and lifestyle factors that could not only influence the strength of association with the outcome but might also lead to information bias since they were self-reported.

In conclusion we believe that our study findings are of importance for clinical practice and public health policy. The relatively high prevalence of high-risk HPV indicates a future risk of anal cancer among MSM in general and among MSM living with HIV in particular. Our results emphasize the urgent need to improve both behavioral changes, frequent STI screening and general health advise as primary and secondary prevention strategies among MSM in Pakistan.

Recommendations: HIV care and treatment programs in Pakistan represent a potential window of opportunity for providing preventive clinical interventions to avert an anal cancer epidemic nested within the HIV epidemic. Providing cost-effective, life-saving anal cancer prevention services (i.e. screen and treat approach and vaccination) linked to HIV care could not only avert preventable HPV related malignancies but could also lead to a more efficient utilization of resources and improve health care access for those most at risk. Moreover, our study findings emphasize the need for government support of interventions that can improve access to and quality of services to MSM including MSW. Prevalent cultural and social stigma must also be addressed since this is essential for protecting the dignity and autonomy of this population.

Contributors: ME, SB conceptualized the study. ME designed the study. AME, SA & TSA supervised the main study implementation. Lab work was supervised by SB & ME. ME did the data collection, data cleaing and mining. ME did the data analysis. ME prepared the first draft of the manuscript with initial

For peer review only - http://bmjopen.bmj.com/site/about/guidelines.xhtml

revisison from SA and TSA. AME did the extensive revisions of the manuscript with ME. All authors reviewed and approved the final version of the manuscript.

Acknowledgements: We thank our all study participants and the staff at our study sites for their continous cooperation and colloboration. Special thanks to Dr Asli Kulane for her constant encouragemnet and guidence to conduct this study. Special thanks to Ms Shazia for her assistanace in the conduct of the lab work.

Financial support:

This resaerch has received no specific grant from any funding agency in the public, commercial or not-for-profit sectors. However, We thank the Sindh Institute of Urology and Transplantation (SIUT) for their lab supplies (reagents and the sample collection kits) and contribution to the lab analyses. We are grateful to the staff at the histopathology and molecular laboratories of SIUT who devoted their time and efforts to this project (in-kind). We are also grateful for the financial contribution from the Dean's Fund at The Aga Khan Hospital & Medical University which covered the cost of samples transporation to the SIUT labs from the two study sites.

Potential conflict of interest: All authors: No potential conflicts of interest

Data availability Statement: The data that support the findings of this study are available from Global & Sexual Health Research Group of Department of Global Public Health on reasonable request and with permission of Karolinska Institutet (KI) Stockholm Sweden

Presentation of this work: This work has been presented in 32nd International Papillomaviris Conference in conjunction with AOGIN 2018, being held in Sydney Australia from October 2-6,2018. This conference attandence was part of my Doctorol Program and financially being supported by Faculty Develoment Award by The Aga Khan Hospital & Medical University Pakistan.

Acceptance of the work: This work has ben accepted in STI and HIV 2019 World Congress (13th to 17th july 2019 at Vancouver Canada).

For peer review only - http://bmjopen.bmj.com/site/about/guidelines.xhtml

REFERENCES:

1. Baseman JG, Koutsky LA. The epidemiology of human papillomavirus infections. *J Clin Virol.* 2005;32 Suppl 1:S16-24.
2. Dunne EF, Unger ER, Sternberg M, et al. Prevalence of HPV infection among females in the United States. *JAMA: The Journal of the American Medical Association.*297(8): 813–819.
3. Machalek DA, Poynten M, Jin F, et al. Anal human papillomavirus infection and associated neoplastic lesions in men who have sex with men: a systematic review and meta-analysis. *Lancet Oncol.* 2012;13(5):487-500.
4. Cranston RD, Carballo-Diéguez A, Gundacker H, et al. Prevalence and determinants of anal human papillomavirus infection in men who have sex with men and transgender women. 2019;30(2):154-162.
5. Somia I. Prevalence of and risk factors for anal high-risk HPV among HIV-negative and HIV-positive MSM and transgender women in three countries at South-East Asia. Med. 97, e9898. In:2018.
6. Chesson HW, Ekwueme DU, Saraiya M, Dunne EF, Markowitz LEJV. The cost-effectiveness of male HPV vaccination in the United States. 2011;29(46):8443-8450.
7. Jemal A, Murray T, Samuels A, Ghafoor A, Ward E, Thun MJ. Cancer statistics, 2003. *CA Cancer J Clin.* 2003;53(1):5-26.
8. Silverberg MJ, Lau B, Justice AC, et al. Risk of anal cancer in HIV-infected and HIV-uninfected individuals in North America. *Clin Infect Dis.* 2012;54(7):1026-1034.
9. Patel P, Hanson DL, Sullivan PS, et al. Incidence of types of cancer among HIV-infected persons compared with the general population in the United States, 1992-2003. *Ann Intern Med.* 2008;148(10):728-736.
10. Houlihan CF, Larke NL, Watson-Jones D, et al. Human papillomavirus infection and increased risk of HIV acquisition. A systematic review and meta-analysis. *Aids.* 2012;26(17):2211-2222.
11. Lawton MD, Nathan M, Asboe D. HPV vaccination to prevent anal cancer in men who have sex with men. *Sex Transm Infect.* 2013;89(5):342-343.
12. Hernandez AL, Efird JT, Holly EA, Berry JM, Jay N, Palefsky JM. Incidence of and risk factors for type-specific anal human papillomavirus infection among HIV-positive MSM. *AIDS.* 2014;28(9):1341-1349.
13. Rajabali A, Khan S, Warraich HJ, Khanani MR, Ali SH. HIV and homosexuality in Pakistan. *The Lancet infectious diseases.* 2008;8(8):511-515.
14. Shaw SY, Emmanuel F, Adrien A, et al. The descriptive epidemiology of male sex workers in Pakistan: a biological and behavioural examination. *Sexually transmitted infections.* 2011;87(1):73-80.
15. Abdullah MA, Basharat Z, Kamal B, et al. Is social exclusion pushing the Pakistani Hijras (Transgenders) towards commercial sex work? A qualitative study. *BMC international health and human rights.* 2012;12(1):32.
16. Second-generation HIV surveillance in Pakistan round 5: Integrated Behavior and Biological Survaillance in Pakistan 2016-17. 2016-2017.

For peer review only - http://bmjopen.bmj.com/site/about/guidelines.xhtml

17. Mayhew S, Collumbien M, Qureshi A, et al. Protecting the unprotected: mixed-method research on drug use, sex work and rights in Pakistan's fight against HIV/AIDS. *Sexually transmitted infections.* 2009;85(Suppl 2):ii31-ii36.
18. Buse K, Lalji N, Mayhew S, Imran M, Hawkes S. Political feasibility of scaling-up five evidence-informed HIV interventions in Pakistan: a policy analysis. *Sexually Transmitted Infections.* 2009;85(Suppl 2):ii37-ii42.
19. Schiller JT, Castellsague X, Garland SM. A review of clinical trials of human papillomavirus prophylactic vaccines. *Vaccine.* 2012;30 Suppl 5:F123-138.
20. Essaada Belglaiaa, Hicham Elannaz, Bouchra Mouaouya, et al. Human papillomavirus genotypes among women with or without HIV infection: an epidemiological study of Moroccan women from the Souss area. *Infectious agents and cancer.* 2015;10(44).
21. Sotlar K, Diemer D, Dethleffs A, et al. Detection and typing of human papillomavirus by e6 nested multiplex PCR. *Journal of clinical microbiology.* 2004;42(7):3176-3184.
22. Kiani SJ, Malekshahi SS, Ghalejoogh ZY, et al. Detection and typing of human papilloma viruses by nested multiplex polymerase chain reaction assay in cervical cancer. 2015;8(12).
23. Phanuphak N, Teeratakulpisarn N, Pankam T, et al. Anal human papillomavirus infection among Thai men who have sex with men with and without HIV infection: prevalence, incidence, and persistence. *J Acquir Immune Defic Syndr.* 2013;63(4):472-479.
24. Ren X, Ke W, Zheng H, et al. Human Papillomavirus Positivity in the Anal Canal in HIV-Infected and HIV-Uninfected Men Who Have Anal Sex with Men in Guangzhou, China: Implication for Anal Exams and Early Vaccination. *Biomed Res Int.* 2017;2017:2641259.
25. Li X, Li M, Yang Y, et al. Anal HPV/HIV co-infection among Men Who Have Sex with Men: a cross-sectional survey from three cities in China. *Sci Rep.* 2016;6:21368.
26. Hernandez AL, Karthik R, Sivasubramanian M, et al. Prevalence of Anal HPV Infection Among HIV-Positive Men Who Have Sex With Men in India. *J Acquir Immune Defic Syndr.* 2016;71(4):437-443.
27. de Pokomandy A, Rouleau D, Ghattas G, et al. Prevalence, clearance, and incidence of anal human papillomavirus infection in HIV-infected men: the HIPVIRG cohort study. *J Infect Dis.* 2009;199(7):965-973.
28. Auvert B, Lissouba P, Cutler E, Zarca K, Puren A, Taljaard D. Association of oncogenic and nononcogenic human papillomavirus with HIV incidence. *J Acquir Immune Defic Syndr.* 2010;53(1):111-116.
29. Critchlow CW, Hawes SE, Kuypers JM, et al. Effect of HIV infection on the natural history of anal human papillomavirus infection. *Aids.* 1998;12(10):1177-1184.
30. Palefsky JM, Holly EA, Ralston ML, Jay N. Prevalence and risk factors for human papillomavirus infection of the anal canal in human immunodeficiency virus (HIV)-positive and HIV-negative homosexual men. *J Infect Dis.* 1998;177:361-367.
31. Goldstone S, Palefsky JM, Giuliano AR, et al. Prevalence of and risk factors for human papillomavirus (HPV) infection among HIV-seronegative men who have sex with men. *J Infect Dis.* 2011;203(1):66-74.
32. Dona MG, Vescio MF, Latini A, et al. Anal human papillomavirus in HIV-uninfected men who have sex with men: incidence and clearance rates, duration of infection, and risk factors. *Clin Microbiol Infect.* 2016;22(12):1004 e1001-1004 e1007.

For peer review only - http://bmjopen.bmj.com/site/about/guidelines.xhtml

33. Liu X, Lin H, Chen X, et al. Prevalence and genotypes of anal human papillomavirus infection among HIV-positive vs. HIV-negative men in Taizhou, China. 2019;147.
34. Sahasrabuddhe VV, Castle PE, Follansbee S, et al. Human papillomavirus genotype attribution and estimation of preventable fraction of anal intraepithelial neoplasia cases among HIV-infected men who have sex with men. *J Infect Dis.* 2013;207(3):392-401.
35. Phanuphak N, Teeratakulpisarn N, Triratanachat S, et al. High prevalence and incidence of high-grade anal intraepithelial neoplasia among young Thai men who have sex with men with and without HIV. *AIDS.* 2013;27(11):1753-1762.
36. Blas MM, Brown B, Menacho L, Alva IE, Silva-Santisteban A, Carcamo C. HPV Prevalence in Multiple Anatomical Sites among Men Who Have Sex with Men in Peru. *PLoS One.* 2015;10(10):e0139524.
37. Rositch AF, Gravitt PE, Smith JS. Growing evidence that HPV infection is associated with an increase in HIV acquisition: exploring the issue of HPV vaccination. In: BMJ Publishing Group Ltd; 2013.
38. Houlihan CF, Larke NL, Watson-Jones D, et al. HPV infection and increased risk of HIV acquisition. A systematic review and meta-analysis. 2012;26(17).
39. van Aar F, Mooij SH, van der Sande MA, et al. Anal and penile high-risk human papillomavirus prevalence in HIV-negative and HIV-infected MSM. *AIDS.* 2013;27(18):2921-2931.
40. Tobian AA, Kigozi G, Gravitt PE, et al. Human papillomavirus incidence and clearance among HIV-positive and HIV-negative men in sub-Saharan Africa. *Aids.* 2012;26(12):1555-1565.
41. Baral SD, Friedman MR, Geibel S, et al. Male sex workers: practices, contexts, and vulnerabilities for HIV acquisition and transmission. *The Lancet.* 2015;385(9964):260-273.
42. Harrison A, Cleland J, Gouws E, Frohlich J. Early sexual debut among young men in rural South Africa: heightened vulnerability to sexual risk? *Sexually transmitted infections.* 2005;81(3):259-261.
43. Noman ul Haq, Sohail Riaz, Aqeel Nasim, Tahir. M. The transgender prostitution: Threat to the rise of AIDS in Pakistan. *Journal of Infection and Public Health.* 2018;11:448.
44. Winkelstein W, Lyman DM, Padian N, et al. Sexual practices and risk of infection by the human immunodeficiency virus: the San Francisco Men's Health Study. *Jama.* 1987;257(3):321-325.
45. Shvetsov YB, Hernandez BY, McDuffie K, et al. Duration and clearance of anal human papillomavirus (HPV) infection among women: the Hawaii HPV cohort study. *Clin Infect Dis.* 2009;48(5):536-546.

For peer review only

TABLE 1: Socio-demographic and life style characteristics of MSM and transgender women in Karachi, Pakistan by HPV status				
**Characteristics**	**Overall (n=298)**	**HPV POSITIVE (n=194)**	**HPV NEGATIVE (n=104)**	**P- value**
**Age: mean (SD)**	28.8(±8.06)	28.9 (7.3)	30.2 (9.1)	0.027
**Age categories in years n (% ages)**				
< 25	102(34.2)	72 (37.1)	30 (28.8)	0.254
25 – 29	90(30.2)	59 (30.4)	31 (29.8)	
30 – 34	41(13.8)	27 (13.9)	14 (13.5)	
>35	65(21.8)	36 (18.6)	29 (27.9)	
**Education n (% ages)**				
None	83(27.9)	56 (29.2)	27 (26.2)	0.334
Primary school	36 (12.1)	29 (15.1)	07 (6.8)	
Middle school	41 (13.8)	24 (12.5)	17 (16.5)	
Matric school	78 (26.2)	49 (25.5)	29 (28.2)	
Intermediate	38 (12.8)	23 (12.0)	15 (14.6)	
Graduate	19 (6.4)	11 (5.7)	08 (7.8)	
**Marital Status n (% ages)**				
Unmarried	234 (78.5)	156 (80.4)	78 (75.0)	
Married	57 (19.1)	32 (16.5)	25 (24.0)	0.256
Separated/divorced	7 (2.3)	06 (3.1)	01 (1.0)	
**Smoking status n (% ages)**				
Yes	232 (77.9)	160(82.5)	72(69.2)	0.009
**Age at smoking initiation; mean (SD)**	16.5 (±4.9)	15.6 (4.5)	18.5 (5.7)	<0.001
**Average number of cigarettes/day n (% ages)**				
<10	128 (55.2)	82 (52.9)	46(63.9)	0.035
10-20	79 (34.1)	52 (33.5)	24(33.3)	
>20	25 (10.7)	21 (13.5)	2(2.8)	
**Duration of smoking in years: mean (SD)**	12.4 (±8.5)	12.9 5(8.5)	12.2(9.2)	0.568
**Alcohol drinking: n (% ages)**				
Yes	71 (23.8)	44 (23.4)	37 (20.4)	0.555
**Sexual behavior: n (% ages)**				
Bisexual	68 (22.8)	39 (20.1)	29 (27.9)	0.127
Homosexual	167 (56.0)	108 (54.6)	59 (56.71)	
Transgender	63 (21.2)	47 (25.3)	16 (15.4)	
**Male Sex Workers: n(% ages)**	237 (79.5)	155 (79.5)	82 (78.8)	0.830
**Age first had sexual intercourse mean(SD)**	16.42 (±4.7)	15.7(4.3)	17.6 (5.2)	0.001
**Duration of sex work: mean (SD)**	12.42(±8.2)	12.3(7.7)	12.6(9.1)	0.765
**Average monthly income from sex work mean (SD) in Pakistani Rupee**	12 563 (±9055)	12 701 (±9,941)	7 806 (±7,561)	0.001
**Anal receptive sexual partners during the last 6 months* n (% ages)**				
<25	25 (8.4)	16 (8.7)	9 (10.2)	0.004
25 – 50	36 (12.1)	17 (9.3)	19 (21.6)	
51 - 100	89 (29.9)	56 (30.6)	33 (37.5)	
>100	121 (40.6)	94 (51.4)	27 (30.7)	
**Condom use during anal receptive sex n(% ages)**				
Consistent use	72 (24.2)	15 (7.7)	49 (47.1)	<0.001
Inconsistent use	175 (58.7)	141 (72.7)	41 (39.4)	
Never	51 (17.1)	38 (19.6)	14 (13.5)	
**Preferred Anal sex role n(% ages)**				
Mainly receptive	264 (88.6)	173(89.2)	81 (77.9)	0.009
Mainly insertive	34 (11.4)	21 (10.8)	23 (22.1)	
**Other STI (gonorrhea, Trichomonas) n (% ages)**	167 (56.0)	133(68.6)	34(32.7)	<0.001

*missing values = 9.1%

For peer review only - http://bmjopen.bmj.com/site/about/guidelines.xhtml

TABLE 2: Distribution of HPV type among MSM and transgender women in Karachi, Pakistan stratified by HIV/HPV Co-infection and HPV only infection			
**HPV TYPES**	**HIV/HPV co-infection N= 114/131HIV infected**	**Only HPV infection N =80/167**	**ALL (N =194)**
	**n (%)**	**n (%)**	**n (%)**
**Any type**	**114** (58.76) 87.0	**80** (41.2) 48.0	194 (65.1)
**High risk types**			
HPV 16	51 (44.7)	17 (21.2)	68 (35.1)
HPV 18	30 (26.3)	15 (18.8)	45 (23.2)
HPV 31	8 (7.5)	6 (7.0)	14 (7.2)
HPV 33	13 (11.4)	0 (0.0)	13 (6.7)
HPV 35	25 (21.9)	16 (20.0)	41 (21.1)
HPV 45	6 (5.3)	8 (10.0)	14 (7.2)
HPV 52	4 (3.5)	9 (11.3)	13 (6.7)
HPV 58	13 (11.4)	3 (3.8)	16 (8.2)
HPV 59	10 (8.8)	11 (13.8)	21 (10.8)
HPV 56	8 (7.0)	7 (8.8)	15 (7.7)
**Low risk types**			
HPV 6/11	42 (36.8)	49 (61.3)	91 (46.9)
**VACCINE SPECIFIC TYPES**			
**Bivalent vaccine type** HPV 16 & /18	75 (65.8)	32 (40.0)	107 (55.2)
**4-valent vaccine type** HPV 6/11/16/18	94 (82.4)	69 (86.2)	163 (84.0)
***9-valent vaccine type** HPV 6/11/16/18/31 /33/45/52/58	105 (92.1)	76 (95.0)	181 (93.3)#

* Includes 9-valent all types which come as alone, in combination with other 9-valent specific types or in combination with non-9-valent HR types such as HPV type 35, when we excluded HPV 35 (n=33) # 93.3 changed to 76.3

Table 3: Prevalence of any HPV and any high-risk HPV by HIV status and sexual orientation												
	**HIV POSITIVE**						**HIV NEGATIVE**					
	**OVERALL**		**Any HPV**		**Any High-Risk HPV**		**OVERALL**		**ANY HPV**		**Any High-Risk HPV**	
	n	%	n	%	n	%	n	%	n	%	n	%
**HOMOSEXUALS**	59	45.03	52	88.13	44	74.57	108	64.67	55	50.92	40	37.03
**BISEXUALS**	39	29.77	30	76.92	20	51.28	29	17.36	9	31.03	6	20.68
**TRANSGENDER**	33	25.20	32	96.96	27	81.81	30	17.96	16	53.33	13	43.33

For peer review only - http://bmjopen.bmj.com/site/about/guidelines.xhtml

TABLE 4: Factors associated with any type of detectable HPV- infection, and HPV type 16, among MSM in transgender women in Karachi, Pakistan (N=298)

RISK FACTORS	Overall N (%)	Any Anal HPV infection Crude PR# (95% CI)	p-value	Any Anal HPV infection Adjusted PR (95% CI)	Anal HPV 16 infection Crude PR (95% CI)	Anal HPV 16 infection Adjusted PR (95% CI)
**HIV status**						
No (ref)	167(56.0)			**1.0**	1.0	**1.0**
Yes	131 (44.0)	1.81 (1.36-2.4)	<0.001	**1.44 (1.05-1.97)**	2.33(1.55-3.51)	**1.81 (1.16-2.82)**
**Other STI***						
No (ref)	131(44.0)	1.0		**1.0**	1.0	1.0
yes	167 (56.0)	1.52 (1.08-2.13)	0.014	**1.39 (1.02-1.91)**	1.97 (1.28-3.04)	1.52 (0.99-2.37)*
**Preferred anal sex role**						
Insertive (ref)	34(11.4)	1.0		1.0	1.0	
Receptive	264 (88.6)	1.95 (1.09-3.50)	0.025	1.56 (0.87-2.83)	2.49 (1.02-6.14)	
**Condom use**						
Consistent (ref)	72 (24.2)	1.0		1.0	1.0	**1.0**
Inconsistent	175 (58.7)	3.30 (1.94-5.6)	<0.001	**2.61 (1.50-4.50)**	2.96 (1.48-5.92)	**1.18 (1.07-4.44)**
never	51 (17.1)	3.11 (1.71-5.66)	<0.001	**3.08 (1.69-5.60)**	2.32 (1.04-5.21)	**2. 31 (1.03-5.20)**
**Smoking**						
No	66 (22.1)	1.0			1.0	
yes	232 (77.9)	1.34 (0.93-1.93)			1.17(0.71-1.90)	

PR: Prevalence ratio; * Trichomonas, Gonorrhea; p-value for multivariate model for anal HPV 16 [0.066]
Adjusted for age, and all other sexual behaviors.

For peer review only

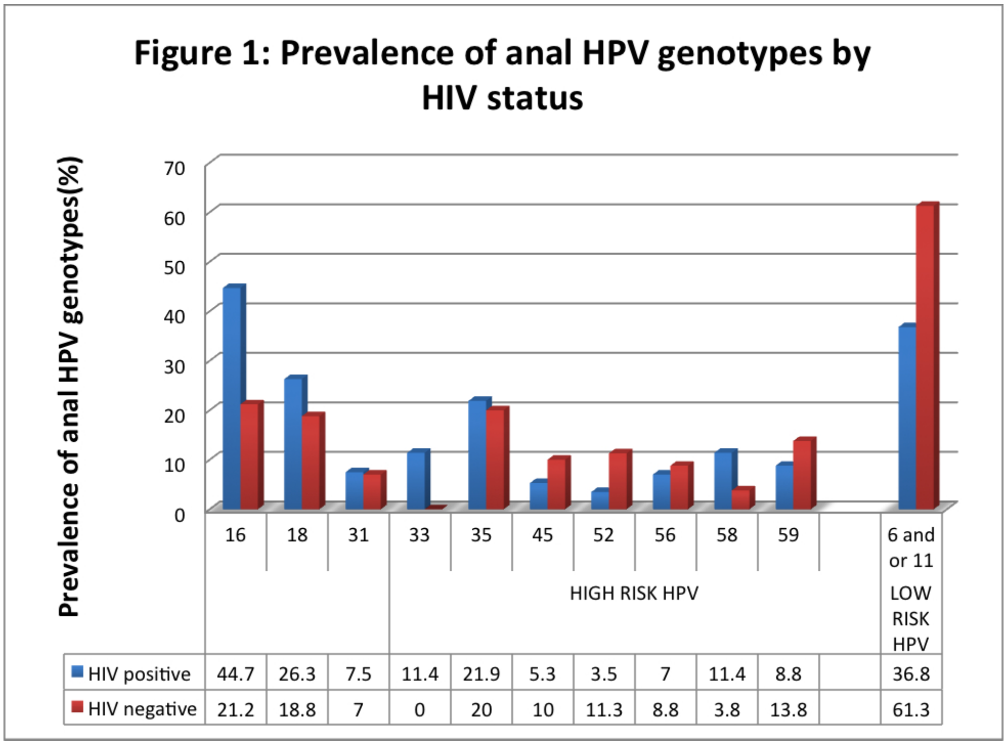

Figure 1: Prevalence of anal HPV genotypes by HIV status
Legend: HPV Human Papilloma virus - HIV Human immunodeficiency virus

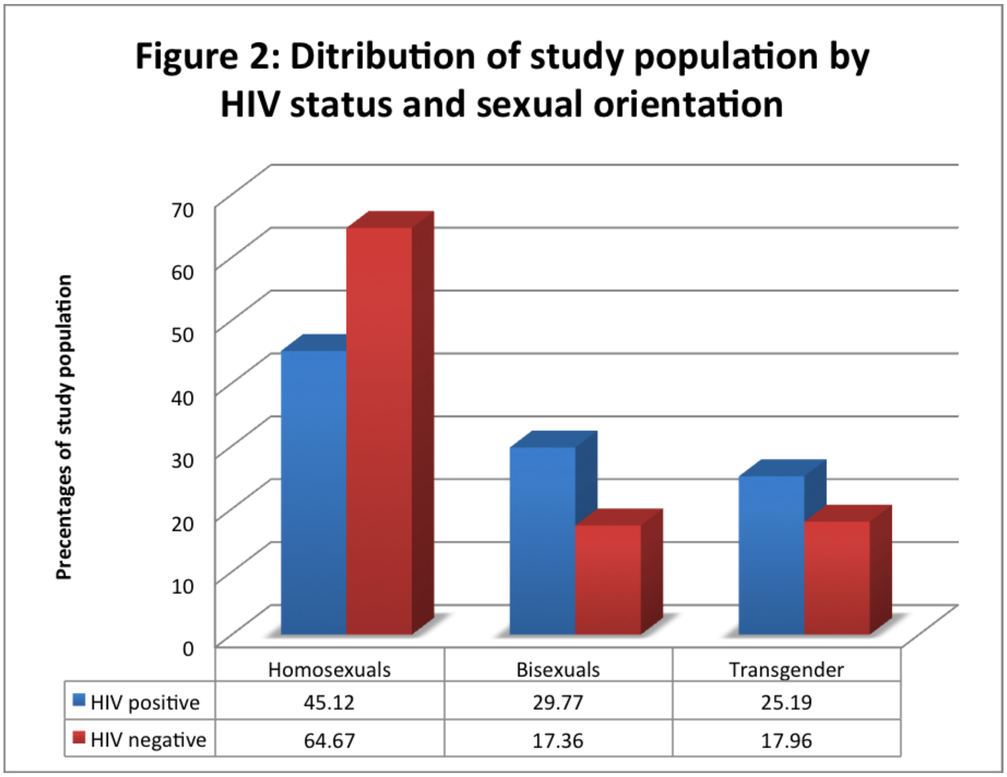

Figure 2: Distribution of study population by HIV status and sexual orientation

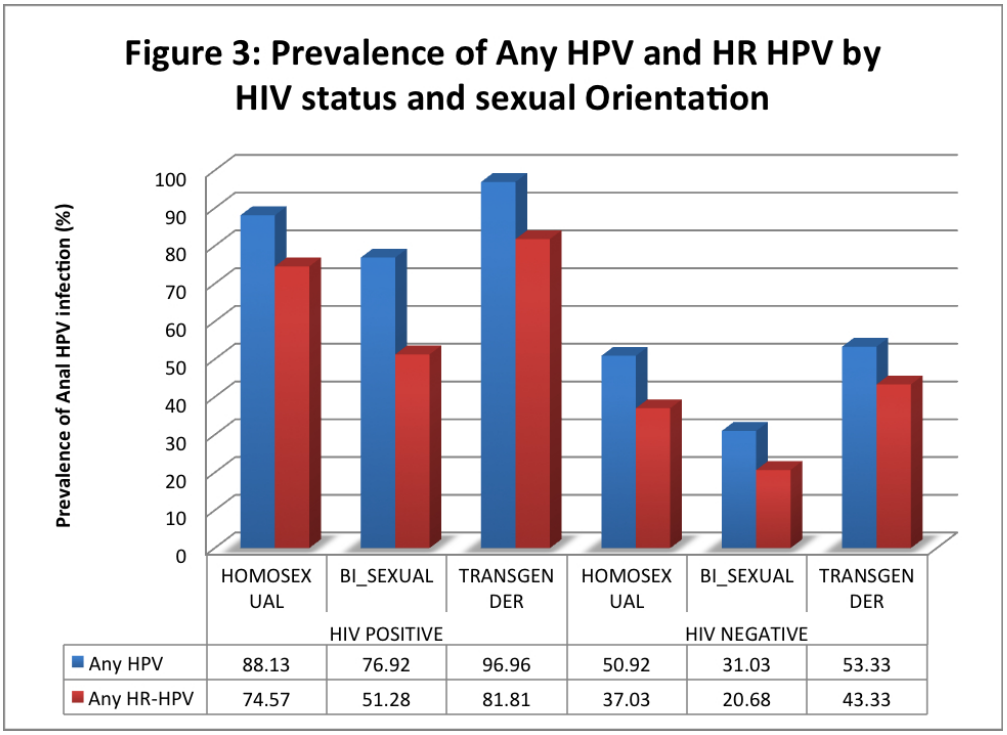

Figure 3: Prevalence of Any HPV and High-risk HPV by HIV status and sexual orientation

RESEARCH CHECKLIST

Type-specific prevalence of anal human papillomavirus infection among men who have sex with men and transgender women in Karachi Pakistan: Implications for prevention strategies

Muslima Ejaz, MD MSc[1,2] Sören Andersson MD, PhD[3], Salma Batool PhD[4], Tazeen Saeed Ali PhD[5], Anna Mia Ekström MD MPH PhD[1]

	PAGE NUMBER
Title	1
Abstract	2
**Introduction:**	
Background/rationale	3
Objective	4
**METHODS**	
Study Design	4
Setting	4
Participants	4
Variables	5
Data Sources/measurement	5
Study size	5
Quantitative variables	6
Statistical methods	6
**RESULTS**	
Participants	6
Descriptive data	6
Outcome data	7
Main results	7
**DISCUSSION**	
Key results	8
Limitations	9
Generalisability	9
Interpretation	10
**Other information**	
Contribution	11
Acknowledgement	11
Funding	11
Conflict of interest	11
Presentation of this work	11

For peer review only

BMJ Open

Anal human papillomavirus infection among men who have sex with men and transgender women living with and without HIV in Pakistan: Findings from the cross-sectional study

Journal:	*BMJ Open*
Manuscript ID	bmjopen-2021-052176.R1
Article Type:	Original research
Date Submitted by the Author:	14-Aug-2021
Complete List of Authors:	Ejaz, Muslima; Aga Khan University Medical College Pakistan, Community Health Sciences; Karolinska Institutet, Public Health Department<br>Andersson, Soren; Orebro Universitet, Lab Medicine<br>Batool, Salma; Sindh Institute of Urology and Transplantation, Molecular Biology<br>Ali, Tazeen; Aga Khan University Medical College Pakistan, Community Health Sciences & School of Nursing<br>Ekström, Anna; Karolinska Institutet, Department of Public Health Sciences
**Primary Subject Heading**:	Epidemiology
Secondary Subject Heading:	Sexual health, Public health, Infectious diseases, HIV/AIDS, Health policy
Keywords:	Epidemiology < INFECTIOUS DISEASES, HIV & AIDS < INFECTIOUS DISEASES, MOLECULAR BIOLOGY, PUBLIC HEALTH, SEXUAL MEDICINE

SCHOLARONE™ Manuscripts

I, the Submitting Author has the right to grant and does grant on behalf of all authors of the Work (as defined in the below author licence), an exclusive licence and/or a non-exclusive licence for contributions from authors who are: i) UK Crown employees; ii) where BMJ has agreed a CC-BY licence shall apply, and/or iii) in accordance with the terms applicable for US Federal Government officers or employees acting as part of their official duties; on a worldwide, perpetual, irrevocable, royalty-free basis to BMJ Publishing Group Ltd ("BMJ") its licensees and where the relevant Journal is co-owned by BMJ to the co-owners of the Journal, to publish the Work in this journal and any other BMJ products and to exploit all rights, as set out in our licence.

The Submitting Author accepts and understands that any supply made under these terms is made by BMJ to the Submitting Author unless you are acting as an employee on behalf of your employer or a postgraduate student of an affiliated institution which is paying any applicable article publishing charge ("APC") for Open Access articles. Where the Submitting Author wishes to make the Work available on an Open Access basis (and intends to pay the relevant APC), the terms of reuse of such Open Access shall be governed by a Creative Commons licence – details of these licences and which Creative Commons licence will apply to this Work are set out in our licence referred to above.

Other than as permitted in any relevant BMJ Author's Self Archiving Policies, I confirm this Work has not been accepted for publication elsewhere, is not being considered for publication elsewhere and does not duplicate material already published. I confirm all authors consent to publication of this Work and authorise the granting of this licence.

For peer review only

Anal human papillomavirus infection among men who have sex with men and transgender women living with and without HIV in Pakistan: Findings from the cross-sectional study

Muslima Ejaz, MD MSc[1,2] Sören Andersson MD, PhD[3], Salma Batool PhD[4], Tazeen Saeed Ali PhD[5], Anna Mia Ekström MD MPH PhD[1]

[1]Department of Global Public Health, Karolinska Institutet Stockholm Sweden

[2] Department of Community Health Sciences the Aga Khan University Karachi Pakistan

[3] Department of Laboratory Medicine, Faculty of Medicine and Health, Örebro University, Örebro, Sweden

[4] Department Molecular Biology, Sindh Institute of Urology and Transplantation Karachi Pakistan

[5] School of Nursing, The Aga Khan University Karachi Pakistan

*Corresponding author

Dr. Muslima Ejaz

Doctoral candidate at Department of Global Public Health

Global & Sexual Health Research group (GloSH)

Karolinska Institutet

Stockholm Sweden

Muslima.ejaz@ki.se

Senior Instructor at Department of Community Health Sciences

Aga Khan University (AKU)

Stadium Road, P.O. Box 3500

Karachi 74800, Pakistan

Phone: 92-21-34864886

Muslima.ejaz@aku.edu

For peer review only

ABSTRACT

Objectives: The aim of this study was to determine the prevalence of infection, genotypes and risk-factors for Human Papillomavirus (HPV) among men who have sex with men (MSM) and transgender-women living with and without HIV in Pakistan. Anal infection with HPV is very common worldwide among MSM, particularly among MSM living-with-HIV**.** The high prevalence of HIV among MSM and male-to-female transgendered individuals in Pakistan is a significant health concern since access to screening and health-seeking is often delayed in this stigmatized key population.

Design: This cross-sectional study was conducted between March-2016-to-November-2017.

Participants, setting and data-collection: This study recruited MSM and transgender-women who self-reported to have had anal-sex in the last 6-months, and were at least 18-years-of-age, from the sexual-health and anti-retroviral-therapy centers. Structured questionnaires were administered, and blood-samples were obtained to confirm HIV-status. Anal-swabs were collected for HPV-DNA detection and typing.

Main outcome measures: The primary outcome was the prevalence of "HPV-DNA infection". The prevalence-ratios (PR) were calculated using Cox-proportional-hazard-model algorithms to analyze the association between exposure variables and HPV-infection.

Results: Complete data were available for 298 MSM and transgender-women (HIV+ n=131; HIV- n=167). The overall HPV-DNA prevalence was 65.1% and was higher in participants living-with-HIV as compared to HIV-negative (87%versus48%; p=<0.001). Likewise, 28.9% of participants living-with-HIV were infected with two or more-than-two types of HPV as compared to 18.8% participants without HIV(p=<0.001). The most frequent HPV-type was HPV6/11(46.9%), followed by HPV16(35.1%), HPV18(23.2%), and HPV35(21.1%). HIV-status (PR:1.81;95%CI 1.16-2.82) and never-condom-use (PR:2.31;95%CI(1.03-5.20)) were independently associated with prevalence of "anal-HPV-type-16 infection" when adjusting for confounding for age, other sexual and behavioral factors e.g., smoking and alcohol consumption.

Conclusion: High prevalence of HPV indicates a substantial future risk of anal cancer in Pakistani MSM and transgender-women, and particularly in those living-with-HIV. Current findings support anal Pap-smear HPV screening for this particular group and vaccination efforts for future generations.

For peer review only - http://bmjopen.bmj.com/site/about/guidelines.xhtml

For peer review only

For peer review only - http://bmjopen.bmj.com/site/about/guidelines.xhtml

Strengths and limitations of this study

STRENGTHS

- This study is the first description of the epidemiology of anal HPV infection among sexual and gender minorities (men who have sex with men, and transgender women) living with HIV in Pakistan.
- Given the strong stigma, homophobia and discrimination against sexual minorities in Pakistan, data collection included a careful process of gaining trust among study participants.
- The results may be of a public health importance in Pakistan, indicating the need for gender-neutral HPV vaccination before sexual debut, targeting both women and men.

LIMITATIONS

- The cross-sectional study design precludes any conclusion on temporal relationships between potential risk factors and the outcome.
- Any under- or over-reporting regarding sexual behaviors or lifestyle factors leading to information bias cannot be excluded.

For peer review only - http://bmjopen.bmj.com/site/about/guidelines.xhtml

INTRODUCTION:

Human papillomaviruses (HPV) is the most common sexually transmitted infection (STI) worldwide[1], and is rapidly acquired after sexual debut[2]. A meta-analysis of global data on HPV in men who have sex with men (MSM) found prevalence rates of anal HPV to be 93% in MSM living with HIV and 64% in HIV-negative MSM, and these rates did not decrease with increasing age[3]. More than 90 genotypes of HPV have been identified and classified either as low risk (LR) types (predominantly 6 and 11) causing genital warts. High risk (HR) HPV types (predominantly HPV 16 and 18) cause cancer of the cervix[4] , its pathogenic role has also been demonstrated by others with oropharyngeal cancers[5,6]. Persistent infection with HPV 16 is the major cause of anal cancer[7,8] . Whilst the burden of HPV-related disease in all men is lower than in all women, certain at-risk groups such as MSM and transgender women[9,10], and particularly those with HIV infection[7], are at high risk for HPV infection, and related anal cancer and its precursor high-grade squamous intraepithelial lesions[11]. The reported incidence of anal cancer is 1 – 2/100,000 in the general population[12], while in MSM it has been reported up to 35 cases per 100,000[13] and even up to 131/100,000 in MSM living with HIV[14,15] regardless if they are on antiretroviral treatment or not [16-18].

In Pakistan, the demography of MSM is broadly classified as: ***MSM*** who are in relationships but outwardly closeted; as Male Sex Workers (***MSW)*** who are males and undertakes receptive anal or oral sexual activity with a man in return of money or other financial benefits; and as ***hijras*** (transvestites/transgender women) who are biologically male with male genitals but function in society as if female and take the receptive role in anal sex [19,20]. The "hijra" is a South-Asian identity traced back many centuries to India, where they were held in high-esteem and were political advisors to kings and bodyguards to queens. Today, however, they often face social exclusion and many engage in commercial sex work[21].

Pakistan is an authoritarian Islamic republic with conservative social values, particularly on issues of sex. MSM, and in particular MSW, face high levels of discrimination and contempt from society and are subject to harassment, sexual violence and detention by the state authorities. Their health-seeking behaviors are often poor due to stigmatization in healthcare settings and for the fear of being identified. As a result, they are reluctant to seek health care not only in public set-ups but also in private facilities. One previous study on sex work and MSW in Pakistan showed that the majority of MSWs are poor, have little knowledge about safe sex, and cannot negotiate the use of condoms and safe sex with their paying clients, predisposing them to acquiring STIs[20].

For peer review only - http://bmjopen.bmj.com/site/about/guidelines.xhtml

Pakistan's national government has lately recognized MSM as a vulnerable population, and prevention and control programs targeting MSMs in its big cities are being organized (of the estimated 46,264 MSM in Pakistan, Karachi is home to an estimated 18,361 MSM, according a survey conducted for mapping key populations in 2015-2016)[22]. However, program service delivery, such as HIV counseling and testing and other STI screening services, is performed by non-governmental and community-based organizations. They are dependent on national and international funders, which has led to fragmented and incomplete coverage of preventive services for this key population.[23,24] Furthermore, the prevalence of HIV in these groups is reaching alarming proportions. Results from the 5th round of Integrated Behavior and Biological second-generation surveillance for HIV/AIDS in Pakistan 2016-17 reported over 9% HIV prevalence in MSM and almost 13% in transgender women living in Karachi[22].

So far there is no research on the burden of HPV infection and HPV-related anal squamous intraepithelial lesions in this key population in Pakistan. Currently, HPV vaccines, in which the bivalent vaccine protects against HPV16/18 and quadrivalent vaccine protects against HPV16/18 and the low-risk types HPV6/11[25], are available in pharmacies in Pakistan but are not yet made available to the target population. The Ministry of Health has not yet included it in their national immunization program for any group in society.

We conducted a cross sectional study in Karachi Pakistan in 2016-17 with the objective to determine the prevalence and risk factors for anal HPV infection including sexual risk behaviors and the association with HIV infection in this dynamic population to inform future policy and prevention strategies.

METHODS

Setting and participants:

Enrollment occurred in two phases for this cross-sectional study. The initial recruitment (from March 2016 to May 2016) started at the ART clinic with transgender women, a center run by the National AIDS Control Program of Pakistan in Civil Hospital Karachi (CHK) - the largest (1,900 bed) tertiary care public hospital of Karachi Pakistan (n=33). Low pace, logistic issues and privacy concerns led to the relocation and setting-up of recruitment center at a sexual health clinic run by a community-based organization, Perwaaz Trust Karachi Pakistan, where the study population was reached using snowball technique and peer referrals. During the second phase between April 2017 to November 2017 a total of 265 MSM and transgender women living with and without HIV were recruited. Thus, any influence from sampling date has been taken care of by adjusting for sexual orientation. MSM and transgender women were eligible

For peer review only - http://bmjopen.bmj.com/site/about/guidelines.xhtml

for the study who self-reported to have had anal sex in the last preceding at least 6 months and were age 18 and above.

Patient and public Involvement: No patients or member of the public were involved in the design, conduct, reporting, or dissemination plans of our study. Data were kept confidential and anonymous and were used for research purposes only.

Study Procedure: The Ethics Committee on Human Research of Aga Khan (3612-CHS-ERC-15) and Dow (IRB-557/DUHS/APPROVAL/2015/84) Universities of Health Sciences approved the study. Verbal and written informed consent were obtained from all eligible and willing participants for all study procedures. For participants who could not read or write their name, a thumb impression was obtained. Participants were informed that they were free to interrupt the interview or withdraw from the study at any time without any negative consequences in terms of clinical services provided to them. A 35-40-minute-long structured questionnaire was administered at a private space to interview the study participants in order to collect data concerning socio-demographic information, sexual and reproductive history and medical history relevant to HIV, any anal disease and use of ART. A blood sample was obtained for confirmation of their HIV status (through Architect HIV Ag-Ab Combo kit by ABBOTT), viral load (Real time PCR Artus Qiagen) and CD4+ T-cell count (through FACS Count TM flow cytometry, Becton Dickinson Co., Franklin Lakes, NJ, USA). Two anal swabs for HPV testing were collected by a trained general physician. A water-moistened swab was inserted 5 – 6 cm proximal to the anal verge and the samples were obtained from anorectal transition zone around the dentate line in the anal canal. The swab was then agitated vigorously in a tube containing 3 mL of methanol-based fixative - a Sample Transport Medium (UTM-RT viral transport media with flocked polyester swabs, Copan Diagnostics Inc., Corona, CA) and transported to the lab. In Molecular lab the solution was stored at -70 °C before further processing and was later used for the extraction and detection of HPV DNA by the polymerase chain reaction (PCR). The DNA from rectal swab was extracted by using Qiagen DNA mini kit and DNA was eluted in 80 ul of elution buffer and stored at -80 °C for further use. HPV DNA was detected by using MY09/MY11 primers from L1 region resulted in amplification of 450 bp of L1 gene. PCR for Housekeeping gene Glyceraldehyde 3-phosphate dehydrogenase (GAPDH) was done for every extracted DNA and samples tested negative for GAPDH gene were excluded from study. Nested Multiplex PCR (NMPCR) assay was used [26] for detection HPV subtypes included low risk types 16/11 and high-risk (group 1) types 16, 18, 31, 33, 35,52,56,58 and 59 as described by Sotlar et al[27]. Primers for first-round PCR for GP-E6/E7 consensus sequence and second round PCR for HPV subtypes 16, 18, 16/11 31, 33, 35,52,56,58 and 59 were synthesized from Eurofins MWG/Operon Germany. The HPV subtype specific

For peer review only - http://bmjopen.bmj.com/site/about/guidelines.xhtml

primers were used in four cocktails and size of the nested PCR product was used for the identification of each HPV type by gel electrophoresis[28].

Sample size calculation:

For the estimation of HPV prevalence, a sample size of 267 participants were needed with an anticipated prevalence of HPV of 50%, 6% precision and 5% level of significance. For the analysis of factors associated with HPV infection**,** we aimed for a sample of at least 256 participants to achieve 80% power with a range of exposures between 9% to 39% and an anticipated prevalence ratio of 2.5 or more with 5% level of significance. An additional sample of 10% was added to cater for inadequate biological samples.

Data management and Statistical Analysis

The database was analyzed using Statistical Package for Social Sciences (IBM SPSS Statistics for Windows, Version 24.0. Armonk, NY: IBM Corp).

The mean value was calculated with standard deviation for symmetrically distributed variables and independent t-test was used for statistical significance, while the median with interquartile ranges was used for asymmetrical quantitative variables. For categorical variables, proportions were employed; for comparison of proportions between groups, $\chi 2$ or Fisher Exact test (where needed) were used. The overall HPV DNA prevalence (as determined by PCR) is reported as the proportion of HPV positive participants among all included individuals. Conversely, type-specific HPV positivity is presented as the proportion of HPV- subtype positive individuals divided by all HPV positive individuals[7]. Prevalence of "any HPV" and "any HR-HPV" stratified by HIV status for all reported sexual orientations was also assessed using Pearson $\chi 2$. For multiple comparisons for each sexual orientation stratified by HIV status for "any HPV" and "any HR-HPV" Bonferroni Correction Method was used, the p-value threshold was (α/n) = 0.05/6 = 0.0083.

Since the design of the study was cross sectional and the outcome of the interest was common (HPV prevalence); for the association between independent risk factors and the outcome variables "any anal HPV infection" i.e., all those HPV types that we identified in our study sample and "anal HPV **16** infection", we employed a Cox proportion hazard model algorithm to estimate crude and adjusted prevalence ratios (PRs) and their 95% confidence intervals (CIs) in univariate and multivariate models'. Moreover, we employed robust SEs to get the closest CIs. A significance level of p <0.05 was used for the final model.

For peer review only - http://bmjopen.bmj.com/site/about/guidelines.xhtml

RESULTS:

Study Population Characteristics: The final analysis included 298 MSM and transgender women (235 MSM and 63 transgender women) to assess the prevalence of HPV DNA in the anal canal. Out of these 298, 131 (44%) had an HIV-infection and 167 (56%) were HIV-negative. Among MSM living with HIV, 33 (25%) were transgender women. Almost 90% of the participants living with HIV were on ART and two thirds of them had an undetectable HIV viral load.

The overall mean age (±SD) of the 298 recruited participants was 28.8 (±8.06). The level of education was low: 28% reported no formal education and 12% less than primary level. Most respondents were currently not married. Among study participants almost 78% were smokers with mean age of smoking initiation 16.5 (±4.9) and duration of 12 (±8.5) years. Almost 80% were sex workers, with 12.4 (8.5) years mean duration of sex work. The mean age at sexual debut was 16.4 (±4.7) years. Only 24.2% reported consistent condom use during sex (Table 1).

MSM with any anal HPV infection (n=194) were younger than MSM without detectable HPV infection (28.9 versus 30.2; p = 0.027). Smoking was more prevalent among HPV-positive MSM (82.5% versus 69.2%) than among those uninfected, with a significant difference in mean age (±SD) of 15.6 (4.5) at smoking initiation. The mean age (±SD) at sexual debut among study participants with an HPV infection was 15.7 (4.3) versus 17.6 (5.2) (p= 0.001). HPV-infected had more sexual partners, more inconsistent condom users and more often practiced receptive anal sex. (Table 1).

MSM living with HIV were, however, older than MSM who were HIV negative (mean (SD): 30.9 (0.70) versus 27.2(0.59) p=<0.001), more frequently smokers (p=0.01), younger at initiation of smoking (p=<0.001) and had smoked for a longer duration of time (p=<0.001). They also reported younger age at sexual debut (p=<0.001), had more sexual clients (p=<0.001), were mainly the receptive anal partner (p=<0.001), and used condoms less consistently than their HIV negative counterparts (p=<0.001).

Sexual orientation of the study sample:

Among participants living with HIV, 45.1% reported their sexual orientation as homosexual, 29.8% as bisexuals, and 25.1% as transgender women. The sexual orientations reported by HIV uninfected participants were, 64.7% as homosexuals, 17.3% as bisexuals and 18.0 % as transgender women.

HPV DNA Prevalence and Genotype Distribution

Overall, 65.1% (n=194/298) of the total study population was positive for HPV DNA (95% CI: 59.5-70.3%). MSM living with HIV had a significantly higher prevalence of HPV (87% (80.0-91.0%)) compared to those who did not have HIV (48% (40.0-55.0%)) as well as any oncogenic anal HPV infection (83.2% (75.8-

For peer review only - http://bmjopen.bmj.com/site/about/guidelines.xhtml

88.6%) verses (35.3% (28.4-42.8%). Likewise, 28.9% of participants living with HIV were infected with two or more-than-two types of HPV as compared to 18.8% participants without HIV (p=<0.001). The most common HPV types were HPV 6/11 (46.9%), HPV 16 (35.1%), HPV 18 (23.2%), HPV 35 (21.1%), and HPV 58 (8.2%). Moreover, the distribution of HPV 6/11 was more among HIV uninfected participants (61.3%) as compared to HIV infected participants (36.8%) (p-value;0.001). However, HPV type 16 was more common among HIV infected participants (44.7% verses 21.2%) and the difference was statistically significant (p-value; 0.001) (Table 2)

HPV DNA prevalence by HIV status and sexual orientation:

For **participants living with HIV,** the prevalence of "any HPV infection" were 88.1 % for homosexual men, 76.9% for bisexual men and 96.9% for transgender women, and the prevalence for "any high-risk HPV" infection" was 74.5%, 51.3% and 81.8% respectively. (p=0.001) (Figure 1)

For **HIV uninfected participants**, the prevalence of any HPV infection was 50.9 % for homosexual men, 31.0% for bisexual men and 53.4% for transgender women, and the prevalence for any high-risk HPV infection was 37.1%, 20.7% and 43.3% respectively. (p=0.001)

Post-hoc analysis with the Bonferroni Correction Method determined that the homosexuals were significantly different with being infected with "any HPV" and "Any anal HR-HPV" (p-values: 0.0014 and 0.0019) when compared to Bisexuals and Transgender women living with and without HIV

Independent variables associated with anal HPV infection

Being HIV positive (PR 1.81 (95% CI 1.16-2.82) and inconsistent/never condom use (PR 1.18 (1.07-4.44) / (PR 2.31 (1.03-5.20) were independent risk factors associated with increased prevalence of HPV type 16 infection, adjusted for all other demographic, sexual and behavioral risk factors (Table 3).

DISCUSSION

To the best of our knowledge, this is the first report on anal HPV infection among MSM living with and without HIV in Pakistan. The high prevalence of anal HPV among MSM living with HIV (87%) in Pakistan is similar to Thailand (85.0%)[29] and China (81% - 82%) [30,31], slightly lower than in neighboring India (95%)[32] and Canada (98%)[33], but significantly higher than what has reported from the Netherlands (65%) [34]. In HIV-negative MSM, however, studies previously carried out in the United States and Latin America showed similar prevalence levels of HPV prevalence ranging from 42% to 66%, which is comparable to our 48% [35-37]. In Pakistan, there is heterogeneity among MSMs, and it is predominantly MSWs who

For peer review only - http://bmjopen.bmj.com/site/about/guidelines.xhtml

register with the community-based organizations with a hope to get free medical services of any kind in a secluded setting. It is MSW who accounted for 80% of our study population, partly explaining the differences in the prevalence of anal HPV infection between our study and others. In a study from India, which has similar social and cultural values, Hernandez et al [32] noticed HPV prevalence of 98% in MSM living with HIV and commented on the fluid nature of sexual identity without mentioning the exact prevalence of MSW among them. Moreover, given the fact that our study has a cross-sectional design, incubation time for infection and variation in viral clearance could be other possible explanations for the differences in the prevalence.[29,38]

For the distribution of anal HPV types, our study results are similar to the studies done elsewhere[39]. HPV 16 (35.1%) was the most common anal oncogenic HPV type detected both in MSM living with and without HIV (44.7% versus 21.2%, $p < 0.001$). This is in close approximation of the summary prevalence for HPV 16 calculated from a recent meta-analyses[7,40] and by others [29,33,41,42]. HPV 16 has the ability to persist in the anal canal more than any other types and not only tends to cause AIN [29] but also associated with disease severity[7].

The prevalence of HR-HPV type 35 (21.1%) in our study was similar to the levels reported India (20%)[32], but higher than HPV type 35 levels reported from other parts of the world [31-33]. Given the fact that HPV 35 is not included in the FDA-approved 9-valent vaccine, its relatively high prevalence can have implications on anal cancer prevention in our region.

Similar to previous research [43], our results also revealed that almost 4 out of 5 MSM carried at least one of the high-risk genotypes in their anal canal (80% MSM living with HIV versus 73% of MSM without HIV infection). This is of concern in HIV-negative MSM in particular, since infection with HR-HPV types is reported to increase the likelihood of acquiring HIV[44]. Thus, our results further emphasize the need to screen Pakistani MSM more frequently for both HPV and HIV given their high risk of development of malignant disease.

Our study detected a high prevalence of multiple HPV types – i.e., at least 2 types (either low-risk and high risk or high-risk & high-risk) – of 47% in HPV positive MSM as a group, which is similar to other, studies [45]. This indeed is a matter of concern as anal infection with multiple HR-HPV types increases the likelihood of an individual to be infected with HPV-16 and, infection with HPV 16 has been associated with AIN and progression to higher grade AIN over time[3]. Moreover, its prevalence was significantly higher in MSM living with HIV. Higher HPV reinfection rates and lower clearance rates in individuals living with HIV may also play a crucial role in the development and the progression of anal neoplastic disease[29,46].

For peer review only - http://bmjopen.bmj.com/site/about/guidelines.xhtml

The results of our study suggest that the most frequently distributed HPV types among Pakistani MSM could be covered by the currently available nonavalent (93.3%) and quadrivalent (84%) vaccines, which means that most of HPV-related diseases including cancers and anogenital warts may potentially be preventable if offered to MSM at initiation of sexual debut. However, the 20% carrying HPV 35 would not be covered by the currently available vaccines.

More than 50% of HPV-positive MSM were young, below 25 years of age, most were single and sexually very active, and most make a living and support their families through sex work[47]. As many as 80% of HPV-positive MSM had initiated sex work in their teens, which is in line with other studies[48]. The high number of parallel sexual partners may contribute to further spread of HPV and other STIs among MSM.

Among other sexual behaviors, inconsistent or never use of condom was the only variable that was significantly associated with any HPV, specifically HPV 16 infection. For many in our study population, negotiating safe sex is not an option as, on an average, a male sex worker will service 3 to 5 clients per night with a one-time service charge as low as 100 – 150 Pakistani rupees (US$ 0.7 to 1.03)[19 49], though higher rates may be charged for unprotected sex. Thus, financial barriers may impede consistency in condom use. In addition, receptive anal sex was significantly more common among HPV-infected MSM, considering the fact that this is the strong transmission route and poses high risk for HIV acquisition[50]. Since the prevalence of anal HPV was more than twice as common in MSM and transgender women living with HIV, this finding indeed turned out to be consistent with our multiple regression model that identified HIV infection as an independent risk factor for any anal HPV or HPV 16 infection confirming MSM and transgender women living with HIV as a priority for the prevention of anal cancer[40] .

Limitations of the study: Our study had some limitations. First, the design of the study was cross-sectional, yet many HPV infections are non-persistent in nature[51]. Moreover, the cross-sectional design precludes us to determine a temporal relationship, however the risk factors identified in our study appear robust and have also been found in studies from other countries. Second, our study results may have limited generalizability because participants were recruited from only one city of Pakistan (Karachi) and were recruited using peer referrals and snowball sampling techniques. Nevertheless, Karachi is the largest metropolitan city of Pakistan, representing all ethnicities of the country. Moreover, in a country like Pakistan, which is by and large a heteronormative society and where homophobia prevails against this sexual minority due to cultural and religious inhibitions, using above mentioned sampling technique was the most feasible option for the recruitment process. Third, we cannot exclude the possibility of under- or over-reporting of sexual behaviors and lifestyle factors that could not only influence the

For peer review only - http://bmjopen.bmj.com/site/about/guidelines.xhtml

For peer review only

strength of association with the outcome but might also lead to information bias since they were self-reported.

Recommendations: HIV care and treatment programs in Pakistan represent a potential window of opportunity for providing preventive clinical interventions to avert an anal cancer epidemic nested within the HIV epidemic. Providing cost-effective, life-saving anal cancer prevention services (i.e., screen and treat approach and vaccination) linked to HIV care could not only avert preventable HPV-related malignancies but could also lead to a more efficient utilization of resources and improve health care access for those most at risk. Moreover, our study findings emphasize the need for government support of interventions that can improve access to and quality of services to MSM including MSW. Prevalent cultural and social stigma must also be addressed since this is essential for protecting the dignity and autonomy of this population.

Conclusions: We believe that our study findings are of importance for clinical practice and public health policy. The relatively high prevalence of high-risk HPV indicates a future risk of anal cancer among MSM in general and among MSM living with HIV in particular. Given that HPV 35 currently is not included in the 9-valent HPV vaccine, we strongly feel that this is one of our most important contributions and that there is a need for regular anal pap screening among MSM and transgender women to enable early detection and treatment to prevent anal cancer.

Contributors: ME, SB conceptualized the study. ME designed the study. AME, SA & TSA supervised the main study implementation. Lab work was supervised by SB & ME. ME did the data collection, data cleaing and mining. ME did the data analysis. ME prepared the first draft of the manuscript with initial revisison from SA and TSA. AME did the extensive revisions of the manuscript with ME. All authors reviewed and approved the final version of the manuscript.

Acknowledgements: We thank our all study participants and the staff at our study sites for their continous cooperation and colloboration. Special thanks to Dr Asli Kulane for her encouragemnet and guidence to conduct this study. Special thanks to Ms Shazia for her assistanace in the conduct of the lab work.

Financial support: This resaerch has received no specific grant from any funding agency in the public, commercial or not-for-profit sectors. However, we thank the Sindh Institute of Urology and Transplantation (SIUT) for their lab supplies (reagents and the sample collection kits) and contribution to the lab analyses. We are grateful to the staff at the histopathology and molecular laboratories of SIUT who devoted their time and efforts to this project (in-kind). We are also grateful for the financial

For peer review only

For peer review only - http://bmjopen.bmj.com/site/about/guidelines.xhtml

contribution from the Dean's Fund at The Aga Khan Hospital & Medical University which covered the cost of samples transporation to the SIUT labs from the two study sites.

Potential conflict of interest: All authors: No potential conflicts of interest

Data availability Statement: The data that support the findings of this study are available from Global & Sexual Health Research Group of Department of Global Public Health on reasonable request and with permission of Karolinska Institutet (KI) Stockholm Sweden

Presentation of this work: This work has been presented in 32nd International Papillomavirus Conference in conjunction with AOGIN 2018, being held in Sydney Australia from October 2-6,2018. This conference attendance was part of my Doctoral Program and financially being supported by Faculty Development Award by The Aga Khan Hospital & Medical University Pakistan.

Acceptance of the work: This work has ben accepted in STI and HIV 2019 World Congress (13th to 17th july 2019 at Vancouver Canada).

For peer review only

For peer review only - http://bmjopen.bmj.com/site/about/guidelines.xhtml

REFERENCES:

1. Sehnal B, Rozsypal H, Nipčová M, Sláma J. The prevalence, incidence, persistence and transmission ways of human papillomavirus infection (HPV). *Epidemiology.* 2017;66(4):198-209.
2. Zhang L, Regan DG, Ong JJ, et al. Targeted human papillomavirus vaccination for young men who have sex with men in Australia yields significant population benefits and is cost-effective. *Vaccine.* 2017;35(37):4923-4929.
3. Machalek DA, Poynten M, Jin F, et al. Anal human papillomavirus infection and associated neoplastic lesions in men who have sex with men: a systematic review and meta-analysis. *Lancet Oncol.* 2012;13(5):487-500.
4. Fani M, Mahmoodi P, Emadzadeh M, et al. Correlation of human papillomavirus 16 and 18 with cervical cancer and their diagnosis methods in Iranian women: A systematic review and meta-analysis. *Current problems in cancer.* 2020;44(1):100493.
5. Rapado-González Ó, Martínez-Reglero C, Salgado-Barreira Á, et al. Association of Salivary Human Papillomavirus Infection and Oral and Oropharyngeal Cancer: A Meta-Analysis. *Journal of clinical medicine.* 2020;9(5):1305.
6. Nogues JC, Fassas S, Mulcahy C, Zapanta PE. Human papillomavirus-associated head and neck cancer. *The Journal of the American Board of Family Medicine.* 2021;34(4):832-837.
7. Lin C, Franceschi S, Clifford GM. Human papillomavirus types from infection to cancer in the anus, according to sex and HIV status: a systematic review and meta-analysis. *The Lancet Infectious Diseases.* 2018;18(2):198-206.
8. International Agency for Research on Cancer. IARC Monograph on the Evaluation of Carcinogenic Risks to Humans. Biological agents. A review of human carcinogens. *IARC Monogr Eval Carcinog Risks Hum* 2012;100 (B) (1-441).
9. Kobayashi T, Sigel K, Gaisa M. Prevalence of Anal Dysplasia in HIV-infected Transgender Women. *Sexually transmitted diseases.* 2017;44(11):714.
10. Cranston RD, Carballo-Diéguez A, Gundacker H, et al. Prevalence and determinants of anal human papillomavirus infection in men who have sex with men and transgender women. *International journal of STD.* 2019;30(2):154-162.
11. Somia IKA, Teeratakulpisarn N, Jeo WS, et al. Prevalence of and risk factors for anal high-risk HPV among HIV-negative and HIV-positive MSM and transgender women in three countries at South-East Asia. *Medicine.* 2018;97(10).
12. Siegel R, Naishadham D, Jemal A. Cancer statistics, 2012.CA. *Cancer J Clin.* 2012;62:10–29.
13. Jemal A, Murray T, Samuels A, Ghafoor A, Ward E, Thun MJ. Cancer statistics, 2003. *CA Cancer J Clin.* 2003;53(1):5-26.
14. Silverberg MJ, Lau B, Justice AC, et al. Risk of anal cancer in HIV-infected and HIV-uninfected individuals in North America. *Clin Infect Dis.* 2012;54(7):1026-1034.
15. Patel P, Hanson DL, Sullivan PS, et al. Incidence of types of cancer among HIV-infected persons compared with the general population in the United States, 1992-2003. *Ann Intern Med.* 2008;148(10):728-736.
16. Houlihan CF, Larke NL, Watson-Jones D, et al. Human papillomavirus infection and increased risk of HIV acquisition. A systematic review and meta-analysis. *AIDS.* 2012;26(17):2211-2222.

For peer review only - http://bmjopen.bmj.com/site/about/guidelines.xhtml

17. Lawton MD, Nathan M, Asboe D. HPV vaccination to prevent anal cancer in men who have sex with men. *Sex Transm Infect.* 2013;89(5):342-343.
18. Hernandez AL, Efird JT, Holly EA, Berry JM, Jay N, Palefsky JM. Incidence of and risk factors for type-specific anal human papillomavirus infection among HIV-positive MSM. *AIDS.* 2014;28(9):1341-1349.
19. Rajabali A, Khan S, Warraich HJ, Khanani MR, Ali SH. HIV and homosexuality in Pakistan. *The Lancet infectious diseases.* 2008;8(8):511-515.
20. Shaw SY, Emmanuel F, Adrien A, et al. The descriptive epidemiology of male sex workers in Pakistan: a biological and behavioural examination. *Sexually transmitted infections.* 2011;87(1):73-80.
21. Abdullah MA, Basharat Z, Kamal B, et al. Is social exclusion pushing the Pakistani Hijras (Transgenders) towards commercial sex work? A qualitative study. *BMC international health and human rights.* 2012;12(1):32.
22. Second-generation HIV surveillance in Pakistan round 5: Integrated Behavior and Biological Survaillance in Pakistan 2016-17. 2016-2017.
23. Mayhew S, Collumbien M, Qureshi A, et al. Protecting the unprotected: mixed-method research on drug use, sex work and rights in Pakistan's fight against HIV/AIDS. *Sexually transmitted infections.* 2009;85(Suppl 2):ii31-ii36.
24. Buse K, Lalji N, Mayhew S, Imran M, Hawkes S. Political feasibility of scaling-up five evidence-informed HIV interventions in Pakistan: a policy analysis. *Sexually Transmitted Infections.* 2009;85(Suppl 2):ii37-ii42.
25. Schiller JT, Castellsague X, Garland SM. A review of clinical trials of human papillomavirus prophylactic vaccines. *Vaccine.* 2012;30 Suppl 5:F123-138.
26. Essaada Belglaiaa, Hicham Elannaz, Bouchra Mouaouya, et al. Human papillomavirus genotypes among women with or without HIV infection: an epidemiological study of Moroccan women from the Souss area. *Infectious agents and cancer.* 2015;10(44).
27. Sotlar K, Diemer D, Dethleffs A, et al. Detection and typing of human papillomavirus by e6 nested multiplex PCR. *Journal of clinical microbiology.* 2004;42(7):3176-3184.
28. Kiani SJ, Malekshahi SS, Ghalejoogh ZY, et al. Detection and typing of human papilloma viruses by nested multiplex polymerase chain reaction assay in cervical cancer. *Jundishapur journal of microbiology.* 2015;8(12).
29. Phanuphak N, Teeratakulpisarn N, Pankam T, et al. Anal human papillomavirus infection among Thai men who have sex with men with and without HIV infection: prevalence, incidence, and persistence. *J Acquir Immune Defic Syndr.* 2013;63(4):472-479.
30. Ren X, Ke W, Zheng H, et al. Human Papillomavirus Positivity in the Anal Canal in HIV-Infected and HIV-Uninfected Men Who Have Anal Sex with Men in Guangzhou, China: Implication for Anal Exams and Early Vaccination. *Biomed Res Int.* 2017;2017:2641259.
31. Li X, Li M, Yang Y, et al. Anal HPV/HIV co-infection among Men Who Have Sex with Men: a cross-sectional survey from three cities in China. *Sci Rep.* 2016;6:21368.
32. Hernandez AL, Karthik R, Sivasubramanian M, et al. Prevalence of Anal HPV Infection Among HIV-Positive Men Who Have Sex With Men in India. *J Acquir Immune Defic Syndr.* 2016;71(4):437-443.

For peer review only - http://bmjopen.bmj.com/site/about/guidelines.xhtml

33. de Pokomandy A, Rouleau D, Ghattas G, et al. Prevalence, clearance, and incidence of anal human papillomavirus infection in HIV-infected men: the HIPVIRG cohort study. *J Infect Dis.* 2009;199(7):965-973.
34. Auvert B, Lissouba P, Cutler E, Zarca K, Puren A, Taljaard D. Association of oncogenic and nononcogenic human papillomavirus with HIV incidence. *J Acquir Immune Defic Syndr.* 2010;53(1):111-116.
35. Critchlow CW, Hawes SE, Kuypers JM, et al. Effect of HIV infection on the natural history of anal human papillomavirus infection. *AIDS.* 1998;12(10):1177-1184.
36. Palefsky JM, Holly EA, Ralston ML, Jay N. Prevalence and risk factors for human papillomavirus infection of the anal canal in human immunodeficiency virus (HIV)-positive and HIV-negative homosexual men. *J Infect Dis.* 1998;177:361-367.
37. Goldstone S, Palefsky JM, Giuliano AR, et al. Prevalence of and risk factors for human papillomavirus (HPV) infection among HIV-seronegative men who have sex with men. *J Infect Dis.* 2011;203(1):66-74.
38. Dona MG, Vescio MF, Latini A, et al. Anal human papillomavirus in HIV-uninfected men who have sex with men: incidence and clearance rates, duration of infection, and risk factors. *Clin Microbiol Infect.* 2016;22(12): 1004. e1-1004. e7.
39. Liu X, Lin H, Chen X, et al. Prevalence and genotypes of anal human papillomavirus infection among HIV-positive vs. HIV-negative men in Taizhou, China. *Epidemiology Infection.* 2019;147.
40. Marra E, Lin C, Clifford GM. Type-specific anal human papillomavirus prevalence among men, according to sexual preference and HIV status: a systematic literature review and meta-analysis. *The Journal of infectious Diseases.* 2019;219(4):590-598.
41. Sahasrabuddhe VV, Castle PE, Follansbee S, et al. Human papillomavirus genotype attribution and estimation of preventable fraction of anal intraepithelial neoplasia cases among HIV-infected men who have sex with men. *J Infect Dis.* 2013;207(3):392-401.
42. Phanuphak N, Teeratakulpisarn N, Triratanachat S, et al. High prevalence and incidence of high-grade anal intraepithelial neoplasia among young Thai men who have sex with men with and without HIV. *AIDS.* 2013;27(11):1753-1762.
43. Blas MM, Brown B, Menacho L, Alva IE, Silva-Santisteban A, Carcamo C. HPV Prevalence in Multiple Anatomical Sites among Men Who Have Sex with Men in Peru. *PLoS One.* 2015;10(10):e0139524.
44. Houlihan CF, Larke NL, Watson-Jones D, et al. HPV infection and increased risk of HIV acquisition. A systematic review and meta-analysis. *AIDS.* 2012;26(17).
45. van Aar F, Mooij SH, van der Sande MA, et al. Anal and penile high-risk human papillomavirus prevalence in HIV-negative and HIV-infected MSM. *AIDS.* 2013;27(18):2921-2931.
46. Tobian AA, Kigozi G, Gravitt PE, et al. Human papillomavirus incidence and clearance among HIV-positive and HIV-negative men in sub-Saharan Africa. *AIDS.* 2012;26(12):1555-1565.
47. Baral SD, Friedman MR, Geibel S, et al. Male sex workers: practices, contexts, and vulnerabilities for HIV acquisition and transmission. *The Lancet.* 2015;385(9964):260-273.

For peer review only - http://bmjopen.bmj.com/site/about/guidelines.xhtml

48. Harrison A, Cleland J, Gouws E, Frohlich J. Early sexual debut among young men in rural South Africa: heightened vulnerability to sexual risk? *Sexually transmitted infections.* 2005;81(3):259-261.

49. Noman ul Haq, Sohail Riaz, Aqeel Nasim, Tahir. M. The transgender prostitution: Threat to the rise of AIDS in Pakistan. *Journal of Infection and Public Health.* 2018;11:448.

50. Winkelstein W, Lyman DM, Padian N, et al. Sexual practices and risk of infection by the human immunodeficiency virus: the San Francisco Men's Health Study. *JAMA.* 1987;257(3):321-325.

51. Alizon S, Murall CL, Bravo IG. Why human papillomavirus acute infections matter. *Viruses.* 2017;9(10):293.

For peer review only

For peer review only - http://bmjopen.bmj.com/site/about/guidelines.xhtml

Table 1: Socio-demographic and lifestyle characteristics of MSM and transgender women in Karachi, Pakistan by HPV status				
**Characteristics**	**Overall (n=298)**	**HPV POSITIVE (n=194)**	**HPV NEGATIVE (n=104)**	**P- value**
**Age categories in years n (%)**				
< 25	102(34.2)	72 (37.1)	30 (28.8)	
25 – 29	90 (30.2)	59 (30.4)	31 (29.8)	0.254
30 – 34	41(13.8)	27 (13.9)	14 (13.5)	
>35	65(21.8)	36 (18.6)	29 (27.9)	
**Education n (%)**				
None	83(27.9)	56 (29.2)	27 (26.2)	0.334
Primary school	36 (12.1)	29 (15.1)	07 (6.8)	
Middle school	41 (13.8)	24 (12.5)	17 (16.5)	
Matric school	78 (26.2)	49 (25.5)	29 (28.2)	
Intermediate	38 (12.8)	23 (12.0)	15 (14.6)	
Graduate	19 (6.4)	11 (5.7)	08 (7.8)	
**Marital Status n (%)**				
Unmarried	234 (78.5)	156 (80.4)	78 (75.0)	
Married	57 (19.1)	32 (16.5)	25 (24.0)	0.256
Separated/divorced	7 (2.3)	06 (3.1)	01 (1.0)	
**Smoking status n (%)**				
Yes	232 (77.9)	160(82.5)	72(69.2)	0.009
**Average number of cigarettes/day n (%)**				
<10	128 (55.2)	82 (52.9)	46(63.9)	0.035
10-20	79 (34.1)	52 (33.5)	24(33.3)	
>20	25 (10.7)	21 (13.5)	2(2.8)	
**Alcohol drinking: n (%)**				
Yes	71 (23.8)	44 (23.4)	37 (20.4)	0.555
**Sexual behavior: n (%)**				
Bisexual	68 (22.8)	39 (20.1)	29 (27.9)	0.127
Homosexual	167 (56.0)	108 (54.6)	59 (56.71)	
Transgender	63 (21.2)	47 (25.3)	16 (15.4)	
**Male Sex Workers: n (%)**	237 (79.5)	155 (79.5)	82 (78.8)	0.830
**Anal receptive sexual partners during the last 6 months* n (%)**				
<25	25 (8.4)	16 (8.7)	9 (10.2)	0.004
25 – 50	36 (12.1)	17 (9.3)	19 (21.6)	
51 – 100	89 (29.9)	56 (30.6)	33 (37.5)	
>100	121 (40.6)	94 (51.4)	27 (30.7)	
**Condom use during anal receptive sex n (%)**				
Consistent use	72 (24.2)	15 (7.7)	49 (47.1)	<0.001
Inconsistent use	175 (58.7)	141 (72.7)	41 (39.4)	
Never	51 (17.1)	38 (19.6)	14 (13.5)	
**Preferred Anal sex role n (%)**				
Mainly receptive	264 (88.6)	173(89.2)	81 (77.9)	0.009
Mainly insertive	34 (11.4)	21 (10.8)	23 (22.1)	
**Other STI (gonorrhea, Trichomonas) n (%)**	167 (56.0)	133(68.6)	34(32.7)	<0.001
**Recruitment settings n(%)**				
**ART center**	33 (11)	20(10.3)	13(12.5)	0.566
**CBO**	265(89)	174(89.7)	91(87.5)	

*missing values = 9.1%

For peer review only

For peer review only - http://bmjopen.bmj.com/site/about/guidelines.xhtml

Table 2: Distribution of HPV types among MSM and transgender women in Karachi, Pakistan stratified by HIV Status

HPV TYPES	ALL	HPV among HIV Positive	HPV among HIV negative	p-value
	**n (%)**	**n (%)**	**n (%)**	**p-value**
**Any type**	194 (65.1)	**114** (58.8) 87.0	**80** (41.2) 48.0	<0.0001
**High risk types**				
HPV 16	68 (35.1)	51 (44.7)	17 (21.2)	0.001
HPV 18	45 (23.2)	30 (26.3)	15 (18.8)	0.219
HPV 31	14 (7.2)	8 (7.5)	6 (7.0)	0.898
HPV 33	13 (6.7)	13 (11.4)	0 (0.0)	NC
HPV 35	41 (21.1)	25 (21.9)	16 (20.0)	0.746
HPV 45	14 (7.2)	6 (5.3)	9 (11.3)	0.124
HPV 52	13 (6.7)	4 (3.5)	9 (11.3)	0.043*
HPV 58	16 (8.2)	13 (11.4)	3 (3.8)	0.066*
HPV 59	21 (10.8)	10 (8.8)	11 (13.8)	0.272
HPV 56	15 (7.7)	8 (7.0)	7 (8.8)	0.657
**Low risk types**				
HPV 6/11	91 (46.9)	42 (36.8)	49 (61.3)	0.001
**VACCINE SPECIFIC TYPES**				
**Bivalent vaccine type** HPV 16 & /18	107 (55.2)	75 (65.8)	32 (40.0)	0.000
**4-valent vaccine type** HPV 6/11/16/18	163 (84.0)	94 (82.4)	69 (86.2)	0.478
****9-valent vaccine type** HPV 6/11/16/18/31 /33/45/52/58	181 (93.3) #	105 (92.1)	76 (95.0)	0.427

NC: Not calculated, one of the cell counts was 0

*Fisher's Exact Test

** Includes 9-valent all types which come as alone, in combination with other 9-valent specific types or in combination with non-9-valent HR types such as HPV type 35, when we excluded HPV 35 (n=33)

93.3% changed to 76.3%

For peer review only

For peer review only - http://bmjopen.bmj.com/site/about/guidelines.xhtml

Table 3: factors associated with any type of detectable HPV infection, and HPV type 16, among MSM and transgender women in Karachi Pakistan (N=298)

**Risk factors**	**HPV positive N (%)**	**Any Anal HPV infection Crude PR# (95% CI)**	**Any Anal HPV infection Adjusted PR (95% CI)**	**Anal HPV 16 - infection Crude PR (95% CI)**	**Anal HPV 16 - infection Adjusted PR (95% CI)**
**HIV status**					
No (ref)	80 (48.0)	1.81 (1.36-2.4)	**1.0**	1.0	**1.0**
Yes	114 (87.0)		**1.44 (1.05-1.97)**	2.33(1.55-3.51)	**1.81 (1.16-2.82)**
**Other STI****					
No (ref)	61 (31.4)	1.0	**1.0**	1.0	1.0
yes	133 (68.6.0)	1.52 (1.08-2.13)	**1.39 (1.02-1.91)**	1.97 (1.28-3.04)	1.52 (0.99-2.37)
**Preferred anal sex role**					
Insertive (ref)	21 (10.8)	1.0	1.0	1.0	
Receptive	173 (89.2)	1.95 (1.09-3.50)	1.56 (0.87-2.83)	2.49 (1.02-6.14)	
**Condom use**					
Consistent (ref)	15 (7.7)	1.0	1.0	1.0	**1.0**
Inconsistent	141 (72.7)	3.30 (1.94-5.6)	**2.61 (1.50-4.50)**	2.96 (1.48-5.92)	**1.18 (1.07-4.44)**
never	38 (19.6)	3.11 (1.71-5.66)	**3.08 (1.69-5.60)**	2.32 (1.04-5.21)	**2. 31 (1.03-5.20)**
**Smoking**					
No	34 (17.5)	1.0		1.0	
yes	160 (82.5)	1.34 (0.93-1.93)		1.17(0.71-1.90)	

PR: Prevalence ratio; ** Trichomonas, Gonorrhea *
All significant variables were adjusted for age, sociodemographic and all other sexual behaviors.

For peer review only

For peer review only - http://bmjopen.bmj.com/site/about/guidelines.xhtml

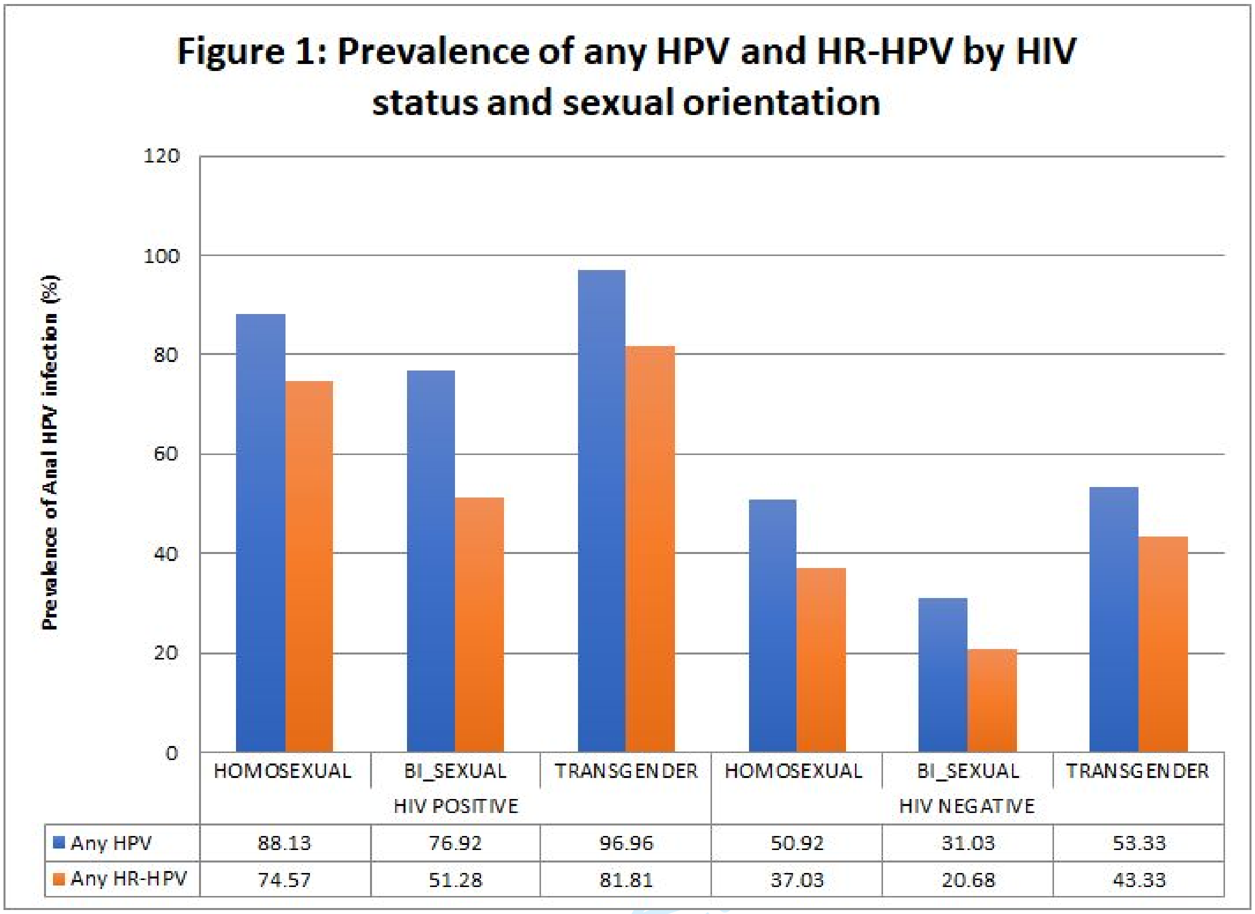

For peer review only - http://bmjopen.bmj.com/site/about/guidelines.xhtml

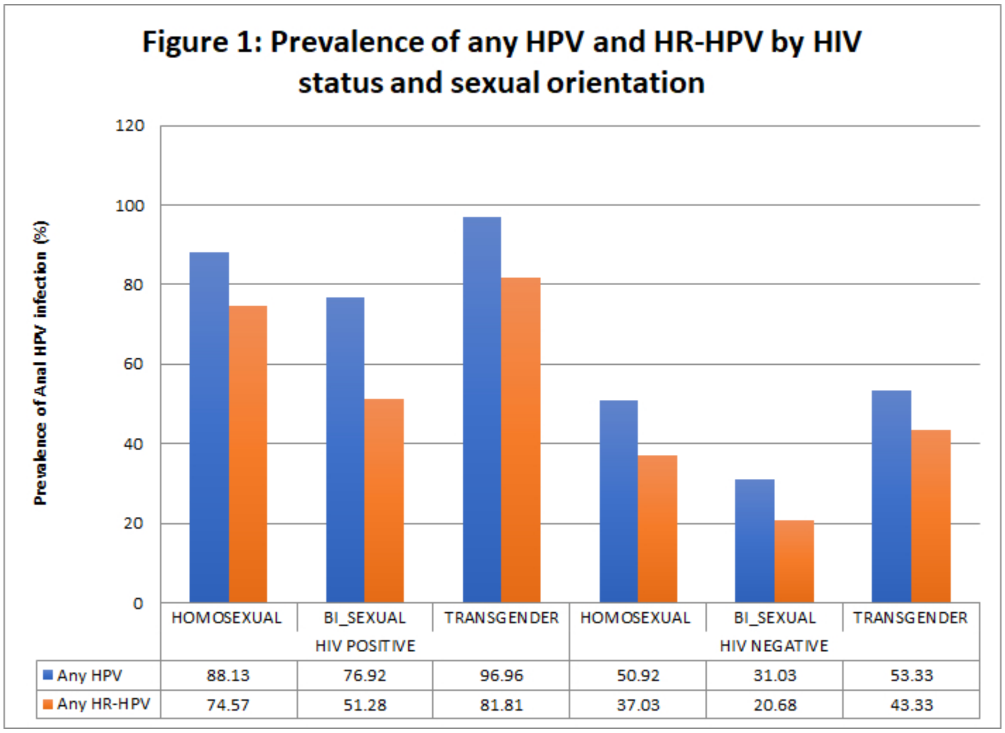

Figure1: Prevalence of any HPV and HR-HPV by HIV status and sexual orientation

187x135mm (96 x 96 DPI)

RESEARCH CHECKLIST

Anal human papillomavirus infection among men who have sex with men and transgender women in Pakistan: Findings from a cross sectional study

Muslima Ejaz, MD MSc[1,2] Sören Andersson MD, PhD[3], Salma Batool PhD[4], Tazeen Saeed Ali PhD[5], Anna Mia Ekström MD MPH PhD[1]

	PAGE NUMBER
Title	1
Abstract	2
**Introduction:**	
Background/rationale	4
Objective	5
**METHODS**	
Study Design	5
Setting	5
Participants	5
Variables	6
Data Sources/measurement	6
Study size	6
Quantitative variables	6
Statistical methods	7
**RESULTS**	
Participants	7
Descriptive data	7
Outcome data	8
Main results	8
**DISCUSSION**	
Key results	9
Limitations	11
Generalisability	11
Interpretation	11
**Other information**	
Contribution	12
Acknowledgement	12
Funding	12
Conflict of interest	12
Presentation of this work	12

For peer review only

BMJ Open

Anal human papillomavirus infection among men who have sex with men and transgender women living with and without HIV in Pakistan: Findings from a cross-sectional study

Journal:	*BMJ Open*
Manuscript ID	bmjopen-2021-052176.R2
Article Type:	Original research
Date Submitted by the Author:	27-Sep-2021
Complete List of Authors:	Ejaz, Muslima; Aga Khan University Medical College Pakistan, Community Health Sciences; Karolinska Institutet, Public Health Department<br>Andersson, Soren; Orebro Universitet, Lab Medicine<br>Batool, Salma; Sindh Institute of Urology and Transplantation, Molecular Biology<br>Ali, Tazeen; Aga Khan University Medical College Pakistan, Community Health Sciences & School of Nursing<br>Ekström, Anna; Karolinska Institutet, Department of Public Health Sciences
**Primary Subject Heading**:	Epidemiology
Secondary Subject Heading:	Sexual health, Public health, Infectious diseases, HIV/AIDS, Health policy
Keywords:	Epidemiology < INFECTIOUS DISEASES, HIV & AIDS < INFECTIOUS DISEASES, MOLECULAR BIOLOGY, PUBLIC HEALTH, SEXUAL MEDICINE

SCHOLARONE™
Manuscripts

I, the Submitting Author has the right to grant and does grant on behalf of all authors of the Work (as defined in the below author licence), an exclusive licence and/or a non-exclusive licence for contributions from authors who are: i) UK Crown employees; ii) where BMJ has agreed a CC-BY licence shall apply, and/or iii) in accordance with the terms applicable for US Federal Government officers or employees acting as part of their official duties; on a worldwide, perpetual, irrevocable, royalty-free basis to BMJ Publishing Group Ltd ("BMJ") its licensees and where the relevant Journal is co-owned by BMJ to the co-owners of the Journal, to publish the Work in this journal and any other BMJ products and to exploit all rights, as set out in our licence.

The Submitting Author accepts and understands that any supply made under these terms is made by BMJ to the Submitting Author unless you are acting as an employee on behalf of your employer or a postgraduate student of an affiliated institution which is paying any applicable article publishing charge ("APC") for Open Access articles. Where the Submitting Author wishes to make the Work available on an Open Access basis (and intends to pay the relevant APC), the terms of reuse of such Open Access shall be governed by a Creative Commons licence – details of these licences and which Creative Commons licence will apply to this Work are set out in our licence referred to above.

Other than as permitted in any relevant BMJ Author's Self Archiving Policies, I confirm this Work has not been accepted for publication elsewhere, is not being considered for publication elsewhere and does not duplicate material already published. I confirm all authors consent to publication of this Work and authorise the granting of this licence.

For peer review only

Anal human papillomavirus infection among men who have sex with men and transgender women living with and without HIV in Pakistan: Findings from a cross-sectional study

Muslima Ejaz, MD MSc[1,2] Sören Andersson MD, PhD[3], Salma Batool PhD[4], Tazeen Saeed Ali PhD[5], Anna Mia Ekström MD MPH PhD[1]

[1]Department of Global Public Health, Karolinska Institutet Stockholm Sweden

[2] Department of Community Health Sciences the Aga Khan University Karachi Pakistan

[3] Department of Laboratory Medicine, Faculty of Medicine and Health, Örebro University, Örebro, Sweden

[4] Department Molecular Biology, Sindh Institute of Urology and Transplantation Karachi Pakistan

[5] School of Nursing, The Aga Khan University Karachi Pakistan

*Corresponding author

Dr. Muslima Ejaz

Doctoral candidate at Department of Global Public Health

Global & Sexual Health Research group (GloSH)

Karolinska Institutet

Stockholm Sweden

Muslima.ejaz@ki.se

Senior Instructor at Department of Community Health Sciences

Aga Khan University (AKU)

Stadium Road, P.O. Box 3500

Karachi 74800, Pakistan

Phone: 92-21-34864886

Muslima.ejaz@aku.edu

For peer review only

ABSTRACT

Objectives: The aim of this study was to determine the prevalence of infection, genotypes and risk-factors for Human Papillomavirus (HPV) among men who have sex with men (MSM) and transgender-women living with and without HIV in Pakistan. Anal infection with HPV is very common worldwide among MSM, particularly among MSM living-with-HIV**.** The high prevalence of HIV among MSM and male-to-female transgendered individuals in Pakistan is a significant health concern since access to screening and health-seeking is often delayed in this stigmatized key population.

Design: This cross-sectional study was conducted between March-2016-to-November-2017.

Participants, setting and data-collection: This study recruited MSM and transgender-women who self-reported to have had anal-sex in the last 6-months, and were at least 18-years-of-age, from the sexual-health and anti-retroviral-therapy centers. Structured questionnaires were administered, and blood-samples were obtained to confirm HIV-status. Anal-swabs were collected for HPV-DNA detection and typing.

Main outcome measures: The primary outcome was the prevalence of "HPV-DNA infection". The prevalence-ratios (PR) were calculated using Cox-proportional-hazard-model algorithms to analyze the association between exposure variables and HPV-infection.

Results: Complete data were available for 298 MSM and transgender-women (HIV+ n=131; HIV- n=167). The overall HPV-DNA prevalence was 65.1% and was higher in participants living-with-HIV as compared to HIV-negative (87%versus48%; χ2p=<0.001). Likewise, 28.9% of participants living-with-HIV were infected with two or more-than-two types of HPV as compared to 18.8% participants without HIV(χ2 p=<0.001). The most frequent HPV-type was HPV6/11(46.9%), followed by HPV16(35.1%), HPV18(23.2%), and HPV35(21.1%). HIV-status (**PR:2.81;95%CI 2.16-3.82**) and never-condom-use (**PR:3.08;95%CI 1.69-5.60)**) were independently associated with prevalence of "**Anal-HPV16 infection**" when adjusting for confounding for age, other sexual and behavioral factors e.g., smoking and alcohol consumption.

Conclusion: High prevalence of HPV indicates a substantial future risk of anal cancer in Pakistani MSM and transgender-women, and particularly in those living-with-HIV. Current findings support anal Pap-smear HPV screening for this particular group and vaccination efforts for future generations.

For peer review only - http://bmjopen.bmj.com/site/about/guidelines.xhtml

Strengths and limitations of this study

STRENGTHS

- This study is the first description of the epidemiology of anal HPV infection among sexual and gender minorities (men who have sex with men, and transgender women) living with HIV in Pakistan.
- Given the strong stigma, homophobia and discrimination against sexual minorities in Pakistan, data collection included a careful process of gaining trust among study participants.
- The results may be of a public health importance in Pakistan, indicating the need for gender-neutral HPV vaccination before sexual debut, targeting both women and men.

LIMITATIONS

- The cross-sectional study design precludes any conclusion on temporal relationships between potential risk factors and the outcome.
- Any under- or over-reporting regarding sexual behaviors or lifestyle factors leading to information bias cannot be excluded.

INTRODUCTION:

For peer review only - http://bmjopen.bmj.com/site/about/guidelines.xhtml

Human papillomaviruses (HPV) is the most common sexually transmitted infection (STI) worldwide[1], and is rapidly acquired after sexual debut[2]. A meta-analysis of global data on HPV in men who have sex with men (MSM) found prevalence rates of anal HPV to be 93% in MSM living with HIV and 64% in HIV-negative MSM, and these rates did not decrease with increasing age[3]. More than 90 genotypes of HPV have been identified and classified either as low-risk (LR) types (predominantly HPV6 and HPV11) causing genital warts. High-risk (HR) HPV types (predominantly HPV16 and HPV18) cause cancer of the cervix[4] , its pathogenic role has also been demonstrated by others with oropharyngeal cancers[5,6]. Persistent infection with HPV16 is the major cause of anal cancer[7,8] . Whilst the burden of HPV-related disease in all men is lower than in all women, certain at-risk groups such as MSM and transgender women[9,10], and particularly those with HIV infection[7], are at high risk for HPV infection, and related anal cancer and its precursor high-grade squamous intraepithelial lesions[11]. The reported incidence of anal cancer is 1 – 2/100,000 in the general population[12], while in MSM it has been reported up to 35 cases per 100,000[13] and even up to 131/100,000 in MSM living with HIV[14,15] regardless if they are on antiretroviral treatment or not [16-18].

In Pakistan, the demography of MSM is broadly classified as: ***MSM*** who are in relationships but outwardly closeted; as Male Sex Workers (***MSW)*** who are males and undertakes receptive anal or oral sexual activity with a man in return of money or other financial benefits; and as ***hijras*** (transvestites/transgender women) who are biologically male with male genitals but function in society as if female and take the receptive role in anal sex [19,20]. The "hijra" is a South-Asian identity traced back many centuries to India, where they were held in high-esteem and were political advisors to kings and bodyguards to queens. Today, however, they often face social exclusion and many engage in commercial sex work[21].

Pakistan is an authoritarian Islamic republic with conservative social values, particularly on issues of sex. MSM, and in particular MSW, face high levels of discrimination and contempt from society and are subject to harassment, sexual violence and detention by the state authorities. Their health-seeking behaviors are often poor due to stigmatization in healthcare settings and for the fear of being identified. As a result, they are reluctant to seek health care not only in public set-ups but also in private facilities. One previous study on sex work and MSW in Pakistan showed that the majority of MSWs are poor, have little knowledge about safe sex, and cannot negotiate the use of condoms and safe sex with their paying clients, predisposing them to acquiring STIs[20].

Pakistan's national government has lately recognized MSM as a vulnerable population, and prevention and control programs targeting MSMs in its big cities are being organized (of the estimated 46,264 MSM in Pakistan, Karachi is home to an estimated 18,361 MSM, according a survey conducted for mapping key populations in 2015-2016)[22]. However, program service delivery, such as HIV counseling and testing and

For peer review only - http://bmjopen.bmj.com/site/about/guidelines.xhtml

other STI screening services, is performed by non-governmental and community-based organizations. They are dependent on national and international funders, which has led to fragmented and incomplete coverage of preventive services for this key population.[23,24] Furthermore, the prevalence of HIV in these groups is reaching alarming proportions. Results from the 5th round of Integrated Behavior and Biological second-generation surveillance for HIV/AIDS in Pakistan 2016-17 reported over 9% HIV prevalence in MSM and almost 13% in transgender women living in Karachi[22].

So far there is no research on the burden of HPV infection and HPV-related anal squamous intraepithelial lesions in this key population in Pakistan. Currently, HPV vaccines, in which the bivalent vaccine protects against HPV16/18 and quadrivalent vaccine protects against HPV16/18 and the low-risk types HPV6/11[25], are available in pharmacies in Pakistan but are not yet made available to the target population. The Ministry of Health has not yet included it in their national immunization program for any group in society.

We conducted a cross sectional study in Karachi Pakistan in 2016-17 with the objective to determine the prevalence and risk factors for anal HPV infection including sexual risk behaviors and the association with HIV infection in this dynamic population to inform future policy and prevention strategies.

METHODS

Setting and participants:

Enrollment occurred in two phases for this cross-sectional study. The initial recruitment (from March 2016 to May 2016) started at the ART clinic with transgender women, a center run by the National AIDS Control Program of Pakistan in Civil Hospital Karachi (CHK) - the largest (1,900 bed) tertiary care public hospital of Karachi Pakistan (recruited n=33). Low pace, logistic issues and privacy concerns led to the relocation and setting-up of recruitment center at a sexual health clinic run by a community-based organization, Perwaaz Trust Karachi Pakistan, where the study population was reached using snowball technique and peer referrals. During the second phase between April 2017 to November 2017 a total of 265 MSM and transgender women living with and without HIV were recruited. Thus, any influence from sampling date has been taken care of by adjusting for sexual orientation. MSM and transgender women were eligible for the study who self-reported to have had anal sex in the last preceding at least 6 months and were age 18 and above.

Patient and public Involvement: No patients or member of the public were involved in the design, conduct, reporting, or dissemination plans of our study. Data were kept confidential and anonymous and were used for research purposes only.

For peer review only - http://bmjopen.bmj.com/site/about/guidelines.xhtml

Study Procedure: The Ethics Committee on Human Research of Aga Khan (3612-CHS-ERC-15) and Dow (IRB-557/DUHS/APPROVAL/2015/84) Universities of Health Sciences approved the study. Verbal and written informed consent were obtained from all eligible and willing participants for all study procedures. For participants who could not read or write their name, a thumb impression was obtained. Participants were informed that they were free to interrupt the interview or withdraw from the study at any time without any negative consequences in terms of clinical services provided to them. A 35-40-minute-long structured questionnaire was administered at a private space to interview the study participants in order to collect data concerning socio-demographic information, sexual and reproductive history and medical history relevant to HIV, any anal disease and use of ART. A blood sample was obtained for confirmation of their HIV status (through Architect HIV Ag-Ab Combo kit by ABBOTT), viral load (Real time PCR Artus Qiagen) and CD4+ T-cell count (through FACS Count TM flow cytometry, Becton Dickinson Co., Franklin Lakes, NJ, USA). Two anal swabs for HPV testing were collected by a trained general physician. A water-moistened swab was inserted 5 – 6 cm proximal to the anal verge and the samples were obtained from anorectal transition zone around the dentate line in the anal canal. The swab was then agitated vigorously in a tube containing 3 mL of methanol-based fixative - a Sample Transport Medium (UTM-RT viral transport media with flocked polyester swabs, Copan Diagnostics Inc., Corona, CA) and transported to the lab. In Molecular lab the solution was stored at -70 °C before further processing and was later used for the extraction and detection of HPV DNA by the polymerase chain reaction (PCR). The DNA from rectal swab was extracted by using Qiagen DNA mini kit and DNA was eluted in 80 ul of elution buffer and stored at -80 °C for further use. HPV DNA was detected by using MY09/MY11 primers from L1 region resulted in amplification of 450 bp of L1 gene. PCR for Housekeeping gene Glyceraldehyde 3-phosphate dehydrogenase (GAPDH) was done for every extracted DNA and samples tested negative for GAPDH gene were excluded from study. Nested Multiplex PCR (NMPCR) assay was used [26] for detection HPV subtypes included low risk types 16/11 and high-risk (group 1) types 16, 18, 31, 33, 35,52,56,58 and 59 as described by Sotlar et al[27]. Primers for first-round PCR for GP-E6/E7 consensus sequence and second round PCR for HPV subtypes 16, 18, 16/11 31, 33, 35,52,56,58 and 59 were synthesized from Eurofins MWG/Operon Germany. The HPV subtype specific primers were used in four cocktails and size of the nested PCR product was used for the identification of each HPV type by gel electrophoresis[28].

Sample size calculation:

For the estimation of HPV prevalence, a sample size of 267 participants were needed with an anticipated prevalence of HPV of 50%, 6% precision and 5% level of significance. For the analysis of factors associated

For peer review only - http://bmjopen.bmj.com/site/about/guidelines.xhtml

with HPV infection**,** we aimed for a sample of at least 256 participants to achieve 80% power with a range of exposures between 9% to 39% and an anticipated prevalence ratio of 2.5 or more with 5% level of significance. An additional sample of 10% was added to cater for inadequate biological samples.

Data management and Statistical Analysis

The database was analyzed using Statistical Package for Social Sciences (IBM SPSS Statistics for Windows, Version 24.0. Armonk, NY: IBM Corp).

The mean value was calculated with standard deviation for symmetrically distributed variables and independent sample t-test was used for statistical significance, while the median with interquartile ranges was used for asymmetrical quantitative variables. For categorical variables, proportions were employed; for comparison of proportions between groups, $\chi 2$ or Fisher Exact test (where needed) were used for p-values. The overall HPV DNA prevalence (as determined by PCR) is reported as the proportion of HPV positive participants among all included individuals. Conversely, type-specific HPV positivity is presented as the proportion of HPV- subtype positive individuals divided by all HPV positive individuals[7]. The confidence intervals for the proportions were estimated using binomial distribution (n,p) assuming a Gaussian error. Prevalence of "Any anal HPV infection" and "Any anal HR-HPV infection" stratified by HIV status for all reported sexual orientations was also assessed using Pearson $\chi 2$. For multiple comparisons for each sexual orientation stratified by HIV status for "Any anal HPV infection" and "Any anal HR-HPV infection" Bonferroni Correction Method was used, the p-value threshold was (α/n) = 0.05/6 = 0.0083.

Since the design of the study was cross sectional and the outcome of the interest was common (HPV prevalence); for the association between independent risk factors and the outcome variables "any anal HPV infection" i.e., all those HPV types that we identified in our study sample and "anal HPV**16** infection", we employed a Cox proportion hazard model algorithm to estimate crude and adjusted prevalence ratios (PRs) and their 95% confidence intervals (CIs) in univariate and multivariate models'. Moreover, we employed robust SEs to get the closest CIs. A significance threshold for the *p value* <0.05 was used for the final model.

RESULTS:

Study Population Characteristics: The final analysis included 298 MSM and transgender women (235 MSM and 63 transgender women) to assess the prevalence of HPV DNA in the anal canal. Out of these 298, 131 (44%) had an HIV-infection and 167 (56%) were HIV-negative. Among MSM living with HIV, 33

For peer review only - http://bmjopen.bmj.com/site/about/guidelines.xhtml

(25%) were transgender women. Almost 90% of the participants living with HIV were on ART and two thirds of them had an undetectable HIV viral load.

The overall mean age (±SD) of the 298 recruited participants was 28.8 (8.06). The level of education was low: 28% reported no formal education and 12% less than primary level. Most respondents were currently not married. Among study participants almost 78% were smokers with mean age of smoking initiation 16.5 (±4.9) and duration of 12 (±8.5) years. Almost 80% were sex workers, with 12.4 (±8.5) years mean duration of sex work. The mean age at sexual debut was 16.4 (±4.7) years. Only 24.2% reported consistent condom use during sex (Table 1).

MSM with any anal HPV infection (n=194) were younger than MSM without detectable HPV infection (28.9 (±7.3) versus 30.2 (±9.1); independent sample t-test p-value = 0.027). Smoking was more prevalent among HPV-positive MSM (82.5% versus 69.2%) than among those uninfected, with a significant difference in mean age (±SD) of 15.6 (4.5) at smoking initiation. The mean age (±SD) at sexual debut among study participants with an HPV infection was 15.7 (4.3) versus 17.6 (5.2) (independent sample t-test-p= 0.001). HPV-infected had more sexual partners, more inconsistent condom users and more often practiced receptive anal sex. (Table 1).

MSM living with HIV were, however, older than MSM who were HIV negative (mean (±SD): 30.9 (0.70) versus 27.2(0.59) independent sample t-test p=<0.001), more frequently smokers (χ2p=0.01), younger at initiation of smoking (independent sample t-test p=<0.001) and had smoked for a longer duration of time ((χ2p=<0.001). They also reported younger age at sexual debut (independent sample t-test p=<0.001), had more sexual clients (χ2p=<0.001), were mainly the receptive anal partner (χ2p=<0.001), and used condoms less consistently than their HIV negative counterparts (χ2p=<0.001).

Sexual orientation of the study sample:

Among participants living with HIV, 45.1% reported their sexual orientation as homosexual, 29.8% as bisexuals, and 25.1% as transgender women. The sexual orientations reported by HIV uninfected participants were, 64.7% as homosexuals, 17.3% as bisexuals and 18.0 % as transgender women.

HPV DNA Prevalence and Genotype Distribution

Overall, 65.1% (n=194/298) of the total study population was positive for HPV DNA (95% CI: 59.5-70.3%). MSM living with HIV had a significantly higher prevalence of HPV (87% (80.0-91.0%)) compared to those who did not have HIV (48% (40.0-55.0%)) as well as any oncogenic anal HPV infection (83.2% (75.8-88.6%) verses (35.3% (28.4-42.8%). Likewise, 28.9% of participants living with HIV were infected with two or more-than-two types of HPV as compared to 18.8% participants without HIV ((χ2p=<0.001). The most

For peer review only

For peer review only - http://bmjopen.bmj.com/site/about/guidelines.xhtml

common HPV types were HPV6/11 (46.9%), HPV16 (35.1%), HPV18 (23.2%), HPV35 (21.1%), and HPV58 (8.2%). Moreover, the distribution of HPV6/11 was more among HIV uninfected participants (61.3%) as compared to HIV infected participants (36.8%) (($\chi 2$p-value;0.001). However, HPV16 was more common among HIV infected participants (44.7% verses 21.2%) and the difference was statistically significant (($\chi 2$ p-value; 0.001) (Table 2)

HPV DNA prevalence by HIV status and sexual orientation:

For **participants living with HIV,** the prevalence of "Any anal HPV infection" were 88.1 % for homosexual men, 76.9% for bisexual men and 96.9% for transgender women, and the prevalence for "Any anal HR-HPV infection" was 74.5%, 51.3% and 81.8% respectively. (($\chi 2$p=0.001) (Figure 1)

For **HIV uninfected participants**, the prevalence of "Any anal HPV infection" was 50.9 % for homosexual men, 31.0% for bisexual men and 53.4% for transgender women, and the prevalence for "Any anal HR-HPV infection" was 37.1%, 20.7% and 43.3% respectively. (($\chi 2$p=0.001)

Post-hoc analysis with the Bonferroni Correction Method determined that the homosexuals were significantly different with being infected with "Any anal HPV infection" and "Any anal HR-HPV infection" (($\chi 2$p-values: 0.0014 and 0.0019) when compared to bisexuals and transgender women living with and without HIV

Independent variables associated with anal HPV infection

Being HIV positive (PR 2.81 (95% CI 2.16-3.82) and inconsistent/never condom use (PR 2.61 (1.50-4.50) / (PR 3.08 (1.69-5.60) were independent risk factors associated with **increased prevalence of HPV16 infection**, adjusted for all other demographic, sexual and behavioral risk factors (Table 3).

DISCUSSION

To the best of our knowledge, this is the first report on anal HPV infection among MSM living with and without HIV in Pakistan. The high prevalence of anal HPV among MSM living with HIV (87%) in Pakistan is similar to Thailand (85.0%)[29] and China (81% - 82%) [30,31], slightly lower than in neighboring India (95%)[32] and Canada (98%)[33], but significantly higher than what has reported from the Netherlands (65%) [34]. In HIV-negative MSM, however, studies previously carried out in the United States and Latin America showed similar prevalence levels of HPV prevalence ranging from 42% to 66%, which is comparable to our 48% [35-37]. In Pakistan, there is heterogeneity among MSMs, and it is predominantly MSWs who register with the community-based organizations with a hope to get free medical services of any kind in a secluded setting.

For peer review only - http://bmjopen.bmj.com/site/about/guidelines.xhtml

It is MSW who accounted for 80% of our study population, partly explaining the differences in the prevalence of anal HPV infection between our study and others. In a study from India, which has similar social and cultural values, Hernandez et al [32] noticed HPV prevalence of 98% in MSM living with HIV and commented on the fluid nature of sexual identity without mentioning the exact prevalence of MSW among them. Moreover, given the fact that our study has a cross-sectional design, incubation time for infection and variation in viral clearance could be other possible explanations for the differences in the prevalence.[29,38]

For the distribution of anal HPV types, our study results are similar to the studies done elsewhere[39]. HPV16 (35.1%) was the most common anal oncogenic HPV type detected both in MSM living with and without HIV (44.7% versus 21.2%, $p < 0.001$). This is in close approximation of the summary prevalence for HPV16 calculated from a recent meta-analyses[7,40] and by others [29,33,41,42]. HPV16 has the ability to persist in the anal canal more than any other types and not only tends to cause AIN [29] but also associated with disease severity[7].

The prevalence of HR-HPV type 35 (21.1%) in our study was similar to the levels reported by India (20%)[32], but higher than HPV type 35 levels reported from other parts of the world [31-33]. Given the fact that HPV35 is not included in the FDA-approved 9-valent vaccine, its relatively high prevalence can have implications on anal cancer prevention in our region.

Similar to previous research [43], our results also revealed that almost 4 out of 5 MSM carried at least one of the high-risk genotypes in their anal canal (80% MSM living with HIV versus 73% of MSM without HIV infection). This is of concern in HIV-negative MSM in particular, since infection with HR-HPV types is reported to increase the likelihood of acquiring HIV[44]. Thus, our results further emphasize the need to screen Pakistani MSM more frequently for both HPV and HIV given their high risk of development of malignant disease.

Our study detected a high prevalence of multiple HPV types – i.e., at least 2 types (either low-risk and high risk or high-risk & high-risk) – of 47% in HPV positive MSM as a group, which is similar to other, studies [45]. This indeed is a matter of concern as anal infection with multiple HR-HPV types increases the likelihood of an individual to be infected with HPV16 and, infection with HPV16 has been associated with AIN and progression to higher grade AIN over time[3]. Moreover, its prevalence was significantly higher in MSM living with HIV. Higher HPV reinfection rates and lower clearance rates in individuals living with HIV may also play a crucial role in the development and the progression of anal neoplastic disease[29,46].

The results of our study suggest that the most frequently distributed HPV types among Pakistani MSM could be covered by the currently available nonavalent (93.3%) and quadrivalent (84%) vaccines, which

For peer review only - http://bmjopen.bmj.com/site/about/guidelines.xhtml

means that most of HPV-related diseases including cancers and anogenital warts may potentially be preventable if offered to MSM at initiation of sexual debut. However, the 20% carrying HPV35 would not be covered by the currently available vaccines.

More than 50% of HPV-positive MSM were young, below 25 years of age, most were single and sexually very active, and most make a living and support their families through sex work[47]. As many as 80% of HPV-positive MSM had initiated sex work in their teens, which is in line with other studies[48]. The high number of parallel sexual partners may contribute to further spread of HPV and other STIs among MSM.

Among other sexual behaviors, inconsistent or never use of condom was the only variable that was significantly associated with any HPV, specifically HPV16 infection. For many in our study population, negotiating safe sex is not an option as, on an average, a male sex worker will service 3 to 5 clients per night with a one-time service charge as low as 100 – 150 Pakistani rupees (US$ 0.7 to 1.03)[19 49], though higher rates may be charged for unprotected sex. Thus, financial barriers may impede consistency in condom use. In addition, receptive anal sex was significantly more common among HPV-infected MSM, considering the fact that this is the strong transmission route and poses high risk for HIV acquisition[50]. Since the prevalence of anal HPV was more than twice as common in MSM and transgender women living with HIV, this finding indeed turned out to be consistent with our multiple regression model that identified HIV infection as an independent risk factor for any anal HPV or HPV16 infection confirming MSM and transgender women living with HIV as a priority for the prevention of anal cancer[40] .

Limitations of the study: Our study had some limitations. First, the design of the study was cross-sectional, yet many HPV infections are non-persistent in nature[51]. Moreover, the cross-sectional design precludes us to determine a temporal relationship, however the risk factors identified in our study appear robust and have also been found in studies from other countries. Second, our study results may have limited generalizability because participants were recruited from only one city of Pakistan (Karachi) and were recruited using peer referrals and snowball sampling techniques. Nevertheless, Karachi is the largest metropolitan city of Pakistan, representing all ethnicities of the country. Moreover, in a country like Pakistan, which is by and large a heteronormative society and where homophobia prevails against this sexual minority due to cultural and religious inhibitions, using above mentioned sampling technique was the most feasible option for the recruitment process. Third, we cannot exclude the possibility of under- or over-reporting of sexual behaviors and lifestyle factors that could not only influence the strength of association with the outcome but might also lead to information bias since they were self-reported.

For peer review only - http://bmjopen.bmj.com/site/about/guidelines.xhtml

Recommendations: HIV care and treatment programs in Pakistan represent a potential window of opportunity for providing preventive clinical interventions to avert an anal cancer epidemic nested within the HIV epidemic. Providing cost-effective, life-saving anal cancer prevention services (i.e., screen and treat approach and vaccination) linked to HIV care could not only avert preventable HPV-related malignancies but could also lead to a more efficient utilization of resources and improve health care access for those most at risk. Moreover, our study findings emphasize the need for government support of interventions that can improve access to and quality of services to MSM including MSW. Prevalent cultural and social stigma must also be addressed since this is essential for protecting the dignity and autonomy of this population.

Conclusions: We believe that our study findings are of importance for clinical practice and public health policy. The relatively high prevalence of high-risk HPV indicates a future risk of anal cancer among MSM in general and among MSM living with HIV in particular. Given that HPV35 currently is not included in the 9-valent HPV vaccine, we strongly feel that this is one of our most important contributions and that there is a need for regular anal pap screening among MSM and transgender women to enable early detection and treatment to prevent anal cancer.

Contributors: ME, SB conceptualized the study. ME designed the study. AME, SA & TSA supervised the main study implementation. Lab work was supervised by SB & ME. ME did the data collection, data cleaing and mining. ME did the data analysis. ME prepared the first draft of the manuscript with initial revisison from SA and TSA. AME did the extensive revisions of the manuscript with ME. All authors reviewed and approved the final version of the manuscript.

Acknowledgements: We thank our all study participants and the staff at our study sites for their continous cooperation and colloboration. Special thanks to Dr Asli Kulane for her encouragemnet and guidence to conduct this study. Special thanks to Ms Shazia for her assistanace in the conduct of the lab work.

Financial support: This resaerch has received no specific grant from any funding agency in the public, commercial or not-for-profit sectors. However, we thank the Sindh Institute of Urology and Transplantation (SIUT) for their lab supplies (reagents and the sample collection kits) and contribution to the lab analyses. We are grateful to the staff at the histopathology and molecular laboratories of SIUT who devoted their time and efforts to this project (in-kind). We are also grateful for the financial contribution from the Dean's Fund at The Aga Khan Hospital & Medical University which covered the cost of samples transporation to the SIUT labs from the two study sites.

Potential conflict of interest: All authors: No potential conflicts of interest

For peer review only

For peer review only - http://bmjopen.bmj.com/site/about/guidelines.xhtml

Data availability Statement: The data that support the findings of this study are available from Global & Sexual Health Research Group of Department of Global Public Health on reasonable request and with permission of Karolinska Institutet (KI) Stockholm Sweden

Presentation of this work: This work has been presented in 32nd International Papillomavirus Conference in conjunction with AOGIN 2018, being held in Sydney Australia from October 2-6,2018. This conference attendance was part of my Doctoral Program and financially being supported by Faculty Development Award by The Aga Khan Hospital & Medical University Pakistan.

Acceptance of the work: This work has ben accepted in STI and HIV 2019 World Congress (13th to 17th july 2019 at Vancouver Canada).

For peer review only

For peer review only - http://bmjopen.bmj.com/site/about/guidelines.xhtml

REFERENCES:

1. Sehnal B, Rozsypal H, Nipčová M, Sláma J. The prevalence, incidence, persistence and transmission ways of human papillomavirus infection (HPV). *Epidemiology.* 2017;66(4):198-209.
2. Zhang L, Regan DG, Ong JJ, et al. Targeted human papillomavirus vaccination for young men who have sex with men in Australia yields significant population benefits and is cost-effective. *Vaccine.* 2017;35(37):4923-4929.
3. Machalek DA, Poynten M, Jin F, et al. Anal human papillomavirus infection and associated neoplastic lesions in men who have sex with men: a systematic review and meta-analysis. *Lancet Oncol.* 2012;13(5):487-500.
4. Fani M, Mahmoodi P, Emadzadeh M, et al. Correlation of human papillomavirus 16 and 18 with cervical cancer and their diagnosis methods in Iranian women: A systematic review and meta-analysis. *Current problems in cancer.* 2020;44(1):100493.
5. Rapado-González Ó, Martínez-Reglero C, Salgado-Barreira Á, et al. Association of Salivary Human Papillomavirus Infection and Oral and Oropharyngeal Cancer: A Meta-Analysis. *Journal of clinical medicine.* 2020;9(5):1305.
6. Nogues JC, Fassas S, Mulcahy C, Zapanta PE. Human papillomavirus-associated head and neck cancer. *The Journal of the American Board of Family Medicine.* 2021;34(4):832-837.
7. Lin C, Franceschi S, Clifford GM. Human papillomavirus types from infection to cancer in the anus, according to sex and HIV status: a systematic review and meta-analysis. *The Lancet Infectious Diseases.* 2018;18(2):198-206.
8. International Agency for Research on Cancer. IARC Monograph on the Evaluation of Carcinogenic Risks to Humans. Biological agents. A review of human carcinogens. *IARC Monogr Eval Carcinog Risks Hum* 2012;100 (B) (1-441).
9. Kobayashi T, Sigel K, Gaisa M. Prevalence of Anal Dysplasia in HIV-infected Transgender Women. *Sexually transmitted diseases.* 2017;44(11):714.
10. Cranston RD, Carballo-Diéguez A, Gundacker H, et al. Prevalence and determinants of anal human papillomavirus infection in men who have sex with men and transgender women. *International journal of STD.* 2019;30(2):154-162.
11. Somia IKA, Teeratakulpisarn N, Jeo WS, et al. Prevalence of and risk factors for anal high-risk HPV among HIV-negative and HIV-positive MSM and transgender women in three countries at South-East Asia. *Medicine.* 2018;97(10).
12. Siegel R, Naishadham D, Jemal A. Cancer statistics, 2012.CA. *Cancer J Clin.* 2012;62:10–29.
13. Jemal A, Murray T, Samuels A, Ghafoor A, Ward E, Thun MJ. Cancer statistics, 2003. *CA Cancer J Clin.* 2003;53(1):5-26.
14. Silverberg MJ, Lau B, Justice AC, et al. Risk of anal cancer in HIV-infected and HIV-uninfected individuals in North America. *Clin Infect Dis.* 2012;54(7):1026-1034.
15. Patel P, Hanson DL, Sullivan PS, et al. Incidence of types of cancer among HIV-infected persons compared with the general population in the United States, 1992-2003. *Ann Intern Med.* 2008;148(10):728-736.
16. Houlihan CF, Larke NL, Watson-Jones D, et al. Human papillomavirus infection and increased risk of HIV acquisition. A systematic review and meta-analysis. *AIDS.* 2012;26(17):2211-2222.

For peer review only - http://bmjopen.bmj.com/site/about/guidelines.xhtml

17. Lawton MD, Nathan M, Asboe D. HPV vaccination to prevent anal cancer in men who have sex with men. *Sex Transm Infect.* 2013;89(5):342-343.
18. Hernandez AL, Efird JT, Holly EA, Berry JM, Jay N, Palefsky JM. Incidence of and risk factors for type-specific anal human papillomavirus infection among HIV-positive MSM. *AIDS.* 2014;28(9):1341-1349.
19. Rajabali A, Khan S, Warraich HJ, Khanani MR, Ali SH. HIV and homosexuality in Pakistan. *The Lancet infectious diseases.* 2008;8(8):511-515.
20. Shaw SY, Emmanuel F, Adrien A, et al. The descriptive epidemiology of male sex workers in Pakistan: a biological and behavioural examination. *Sexually transmitted infections.* 2011;87(1):73-80.
21. Abdullah MA, Basharat Z, Kamal B, et al. Is social exclusion pushing the Pakistani Hijras (Transgenders) towards commercial sex work? A qualitative study. *BMC international health and human rights.* 2012;12(1):32.
22. Second-generation HIV surveillance in Pakistan round 5: Integrated Behavior and Biological Survaillance in Pakistan 2016-17. 2016-2017.
23. Mayhew S, Collumbien M, Qureshi A, et al. Protecting the unprotected: mixed-method research on drug use, sex work and rights in Pakistan's fight against HIV/AIDS. *Sexually transmitted infections.* 2009;85(Suppl 2):ii31-ii36.
24. Buse K, Lalji N, Mayhew S, Imran M, Hawkes S. Political feasibility of scaling-up five evidence-informed HIV interventions in Pakistan: a policy analysis. *Sexually Transmitted Infections.* 2009;85(Suppl 2):ii37-ii42.
25. Schiller JT, Castellsague X, Garland SM. A review of clinical trials of human papillomavirus prophylactic vaccines. *Vaccine.* 2012;30 Suppl 5:F123-138.
26. Essaada Belglaiaa, Hicham Elannaz, Bouchra Mouaouya, et al. Human papillomavirus genotypes among women with or without HIV infection: an epidemiological study of Moroccan women from the Souss area. *Infectious agents and cancer.* 2015;10(44).
27. Sotlar K, Diemer D, Dethleffs A, et al. Detection and typing of human papillomavirus by e6 nested multiplex PCR. *Journal of clinical microbiology.* 2004;42(7):3176-3184.
28. Kiani SJ, Malekshahi SS, Ghalejoogh ZY, et al. Detection and typing of human papilloma viruses by nested multiplex polymerase chain reaction assay in cervical cancer. *Jundishapur journal of microbiology.* 2015;8(12).
29. Phanuphak N, Teeratakulpisarn N, Pankam T, et al. Anal human papillomavirus infection among Thai men who have sex with men with and without HIV infection: prevalence, incidence, and persistence. *J Acquir Immune Defic Syndr.* 2013;63(4):472-479.
30. Ren X, Ke W, Zheng H, et al. Human Papillomavirus Positivity in the Anal Canal in HIV-Infected and HIV-Uninfected Men Who Have Anal Sex with Men in Guangzhou, China: Implication for Anal Exams and Early Vaccination. *Biomed Res Int.* 2017;2017:2641259.
31. Li X, Li M, Yang Y, et al. Anal HPV/HIV co-infection among Men Who Have Sex with Men: a cross-sectional survey from three cities in China. *Sci Rep.* 2016;6:21368.
32. Hernandez AL, Karthik R, Sivasubramanian M, et al. Prevalence of Anal HPV Infection Among HIV-Positive Men Who Have Sex With Men in India. *J Acquir Immune Defic Syndr.* 2016;71(4):437-443.

For peer review only - http://bmjopen.bmj.com/site/about/guidelines.xhtml

33. de Pokomandy A, Rouleau D, Ghattas G, et al. Prevalence, clearance, and incidence of anal human papillomavirus infection in HIV-infected men: the HIPVIRG cohort study. *J Infect Dis.* 2009;199(7):965-973.
34. Auvert B, Lissouba P, Cutler E, Zarca K, Puren A, Taljaard D. Association of oncogenic and nononcogenic human papillomavirus with HIV incidence. *J Acquir Immune Defic Syndr.* 2010;53(1):111-116.
35. Critchlow CW, Hawes SE, Kuypers JM, et al. Effect of HIV infection on the natural history of anal human papillomavirus infection. *AIDS.* 1998;12(10):1177-1184.
36. Palefsky JM, Holly EA, Ralston ML, Jay N. Prevalence and risk factors for human papillomavirus infection of the anal canal in human immunodeficiency virus (HIV)-positive and HIV-negative homosexual men. *J Infect Dis.* 1998;177:361-367.
37. Goldstone S, Palefsky JM, Giuliano AR, et al. Prevalence of and risk factors for human papillomavirus (HPV) infection among HIV-seronegative men who have sex with men. *J Infect Dis.* 2011;203(1):66-74.
38. Dona MG, Vescio MF, Latini A, et al. Anal human papillomavirus in HIV-uninfected men who have sex with men: incidence and clearance rates, duration of infection, and risk factors. *Clin Microbiol Infect.* 2016;22(12): 1004. e1-1004. e7.
39. Liu X, Lin H, Chen X, et al. Prevalence and genotypes of anal human papillomavirus infection among HIV-positive vs. HIV-negative men in Taizhou, China. *Epidemiology Infection.* 2019;147.
40. Marra E, Lin C, Clifford GM. Type-specific anal human papillomavirus prevalence among men, according to sexual preference and HIV status: a systematic literature review and meta-analysis. *The Journal of infectious Diseases.* 2019;219(4):590-598.
41. Sahasrabuddhe VV, Castle PE, Follansbee S, et al. Human papillomavirus genotype attribution and estimation of preventable fraction of anal intraepithelial neoplasia cases among HIV-infected men who have sex with men. *J Infect Dis.* 2013;207(3):392-401.
42. Phanuphak N, Teeratakulpisarn N, Triratanachat S, et al. High prevalence and incidence of high-grade anal intraepithelial neoplasia among young Thai men who have sex with men with and without HIV. *AIDS.* 2013;27(11):1753-1762.
43. Blas MM, Brown B, Menacho L, Alva IE, Silva-Santisteban A, Carcamo C. HPV Prevalence in Multiple Anatomical Sites among Men Who Have Sex with Men in Peru. *PLoS One.* 2015;10(10):e0139524.
44. Houlihan CF, Larke NL, Watson-Jones D, et al. HPV infection and increased risk of HIV acquisition. A systematic review and meta-analysis. *AIDS.* 2012;26(17).
45. van Aar F, Mooij SH, van der Sande MA, et al. Anal and penile high-risk human papillomavirus prevalence in HIV-negative and HIV-infected MSM. *AIDS.* 2013;27(18):2921-2931.
46. Tobian AA, Kigozi G, Gravitt PE, et al. Human papillomavirus incidence and clearance among HIV-positive and HIV-negative men in sub-Saharan Africa. *AIDS.* 2012;26(12):1555-1565.
47. Baral SD, Friedman MR, Geibel S, et al. Male sex workers: practices, contexts, and vulnerabilities for HIV acquisition and transmission. *The Lancet.* 2015;385(9964):260-273.

For peer review only - http://bmjopen.bmj.com/site/about/guidelines.xhtml

48. Harrison A, Cleland J, Gouws E, Frohlich J. Early sexual debut among young men in rural South Africa: heightened vulnerability to sexual risk? *Sexually transmitted infections.* 2005;81(3):259-261.

49. Noman ul Haq, Sohail Riaz, Aqeel Nasim, Tahir. M. The transgender prostitution: Threat to the rise of AIDS in Pakistan. *Journal of Infection and Public Health.* 2018;11:448.

50. Winkelstein W, Lyman DM, Padian N, et al. Sexual practices and risk of infection by the human immunodeficiency virus: the San Francisco Men's Health Study. *JAMA.* 1987;257(3):321-325.

51. Alizon S, Murall CL, Bravo IG. Why human papillomavirus acute infections matter. *Viruses.* 2017;9(10):293.

For peer review only

For peer review only - http://bmjopen.bmj.com/site/about/guidelines.xhtml

Table 1: Socio-demographic and lifestyle characteristics of MSM and transgender women in Karachi, Pakistan by HPV status

Characteristics	Overall (n=298)	HPV POSITIVE (n=194)	HPV NEGATIVE (n=104)	P- value#
**Age categories in years n (%)**				
< 25	102(34.2)	72 (37.1)	30 (28.8)	
25 – 29	90 (30.2)	59 (30.4)	31 (29.8)	0.254
30 – 34	41(13.8)	27 (13.9)	14 (13.5)	
>35	65(21.8)	36 (18.6)	29 (27.9)	
**Education n (%)**				
None	83(27.9)	56 (29.2)	27 (26.2)	0.334
Primary school	36 (12.1)	29 (15.1)	07 (6.8)	
Middle school	41 (13.8)	24 (12.5)	17 (16.5)	
Matric school	78 (26.2)	49 (25.5)	29 (28.2)	
Intermediate	38 (12.8)	23 (12.0)	15 (14.6)	
Graduate	19 (6.4)	11 (5.7)	08 (7.8)	
**Marital Status n (%)**				
Unmarried	234 (78.5)	156 (80.4)	78 (75.0)	
Married	57 (19.1)	32 (16.5)	25 (24.0)	0.256
Separated/divorced	7 (2.3)	06 (3.1)	01 (1.0)	
**Smoking status n (%)**				
Yes	232 (77.9)	160(82.5)	72(69.2)	0.009
**Average number of cigarettes/day n (%)**				
<10	128 (55.2)	82 (52.9)	46(63.9)	0.035
10-20	79 (34.1)	52 (33.5)	24(33.3)	
>20	25 (10.7)	21 (13.5)	2(2.8)	
**Alcohol drinking: n (%)**				
Yes	71 (23.8)	44 (23.4)	37 (20.4)	0.555
**Sexual behavior: n (%)**				
Bisexual	68 (22.8)	39 (20.1)	29 (27.9)	0.127
Homosexual	167 (56.0)	108 (54.6)	59 (56.71)	
Transgender	63 (21.2)	47 (25.3)	16 (15.4)	
**Male Sex Workers: n (%)**	237 (79.5)	155 (79.5)	82 (78.8)	0.830
**Anal receptive sexual partners during the last 6 months* n (%)**				
<25	25 (8.4)	16 (8.7)	9 (10.2)	0.004
25 – 50	36 (12.1)	17 (9.3)	19 (21.6)	
51 – 100	89 (29.9)	56 (30.6)	33 (37.5)	
>100	121 (40.6)	94 (51.4)	27 (30.7)	
**Condom use during anal receptive sex n (%)**				
Consistent use	72 (24.2)	15 (7.7)	49 (47.1)	<0.001
Inconsistent use	175 (58.7)	141 (72.7)	41 (39.4)	
Never	51 (17.1)	38 (19.6)	14 (13.5)	
**Preferred Anal sex role n (%)**				
Mainly receptive	264 (88.6)	173(89.2)	81 (77.9)	0.009
Mainly insertive	34 (11.4)	21 (10.8)	23 (22.1)	
**Other STI (gonorrhea, Trichomonas) n (%)**	167 (56.0)	133(68.6)	34(32.7)	<0.001
**Recruitment settings n(%)**				
**ART center**	33 (11)	20(10.3)	13(12.5)	0.566
**CBO**	265(89)	174(89.7)	91(87.5)	

*missing values = 9.1%

#($\chi 2$ *test was used for* $p = values$)

18

For peer review only

**Table 2: Distribution of HPV types among MSM and transgender women in Karachi, Pakistan stratified by HIV Status**				
**HPV TYPES**	**ALL**	**HPV among HIV Positive**	**HPV among HIV negative**	**P-value##**
	**n (%)**	**n (%)**	**n (%)**	**p-value**
**Any type**	194 (65.1)	**114** (58.8) 87.0	**80** (41.2) 48.0	<0.0001
**High risk types**				
HPV 16	68 (35.1)	51 (44.7)	17 (21.2)	0.001
HPV 18	45 (23.2)	30 (26.3)	15 (18.8)	0.219
HPV 31	14 (7.2)	8 (7.5)	6 (7.0)	0.898
HPV 33	13 (6.7)	13 (11.4)	0 (0.0)	NC
HPV 35	41 (21.1)	25 (21.9)	16 (20.0)	0.746
HPV 45	14 (7.2)	6 (5.3)	9 (11.3)	0.124
HPV 52	13 (6.7)	4 (3.5)	9 (11.3)	0.043*
HPV 58	16 (8.2)	13 (11.4)	3 (3.8)	0.066*
HPV 59	21 (10.8)	10 (8.8)	11 (13.8)	0.272
HPV 56	15 (7.7)	8 (7.0)	7 (8.8)	0.657
**Low risk types**				
HPV 6/11	91 (46.9)	42 (36.8)	49 (61.3)	0.001
**VACCINE SPECIFIC TYPES**				
**Bivalent vaccine type** HPV 16 & /18	107 (55.2)	75 (65.8)	32 (40.0)	0.000
**4-valent vaccine type** HPV 6/11/16/18	163 (84.0)	94 (82.4)	69 (86.2)	0.478
****9-valent vaccine type** HPV 6/11/16/18/31 /33/45/52/58	181 (93.3) #	105 (92.1)	76 (95.0)	0.427

NC: Not calculated, one of the cell counts was 0

*Fisher's Exact Test

** Includes 9-valent all types which come as alone, in combination with other 9-valent specific types or in combination with non-9-valent HR types such as HPV type 35, when we excluded HPV 35 (n=33)

93.3% changed to 76.3%

##($\chi 2$ *test was used for p = values*)

For peer review only

For peer review only - http://bmjopen.bmj.com/site/about/guidelines.xhtml

Table 3: factors associated with any type of detectable HPV infection, and HPV type 16, among MSM and transgender women in Karachi Pakistan (N=298)

**Risk factors**	**HPV positive N (%)**	**Any Anal HPV infection Crude PR# (95% CI)**	**Any Anal HPV infection Adjusted PR (95% CI)**	**Anal HPV 16 - infection Crude PR (95% CI)**	**Anal HPV 16 - infection Adjusted PR (95% CI)**
**HIV status**					
No (ref)	80 (48.0)	1.81 (1.36-2.4)	**1.0**	1.0	**1.0**
Yes	114 (87.0)		**1.44 (1.05-1.97)**	3.33(2.55-3.51)	**2.81 (2.16-3.82)**
**Other STI****					
No (ref)	61 (31.4)	1.0	**1.0**	1.0	1.0
yes	133 (68.6.0)	1.32 (1.08-2.13)	**1.29 (1.02-1.91)**	1.97 (1.28-3.04)	1.52 (0.99-2.37)
**Preferred anal sex role**					
Insertive (ref)	21 (10.8)	1.0	1.0	1.0	
Receptive	173 (89.2)	1.95 (1.09-3.50)	1.56 (0.87-2.83)	2.49 (1.02-6.14)	
**Condom use**					
Consistent (ref)	15 (7.7)	1.0	1.0	1.0	**1.0**
Inconsistent	141 (72.7)	3.30 (1.94-5.6)	**2. 01 (1.07-5.20)**	2.96 (1.48-5.92)	**2.61 (1.50-4.50)**
never	38 (19.6)	2.42 (1.04-5.21)	**1.58 (1.03-4.44)**	3.11 (1.71-5.66)	**3.08 (1.69-5.60)**
**Smoking**					
No	34 (17.5)	1.0		1.0	
yes	160 (82.5)	1.34 (0.93-1.93)		1.17(0.71-1.90)	

PR: Prevalence ratio; ** Trichomonas, Gonorrhea *
All significant variables were adjusted for age, sociodemographic and all other sexual behaviors.

For peer review only

Figure 1: Prevalence of any HPV and HR-HPV by HIV status and sexual orientation

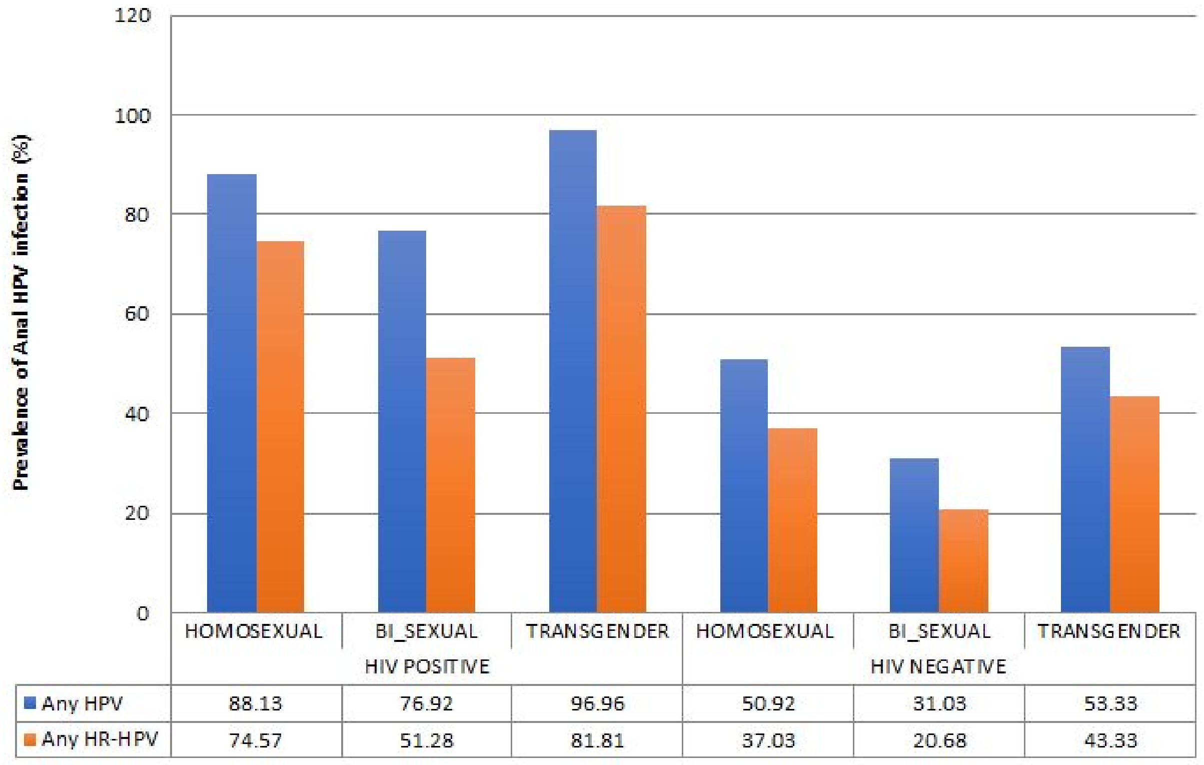

For peer review only - http://bmjopen.bmj.com/site/about/guidelines.xhtml

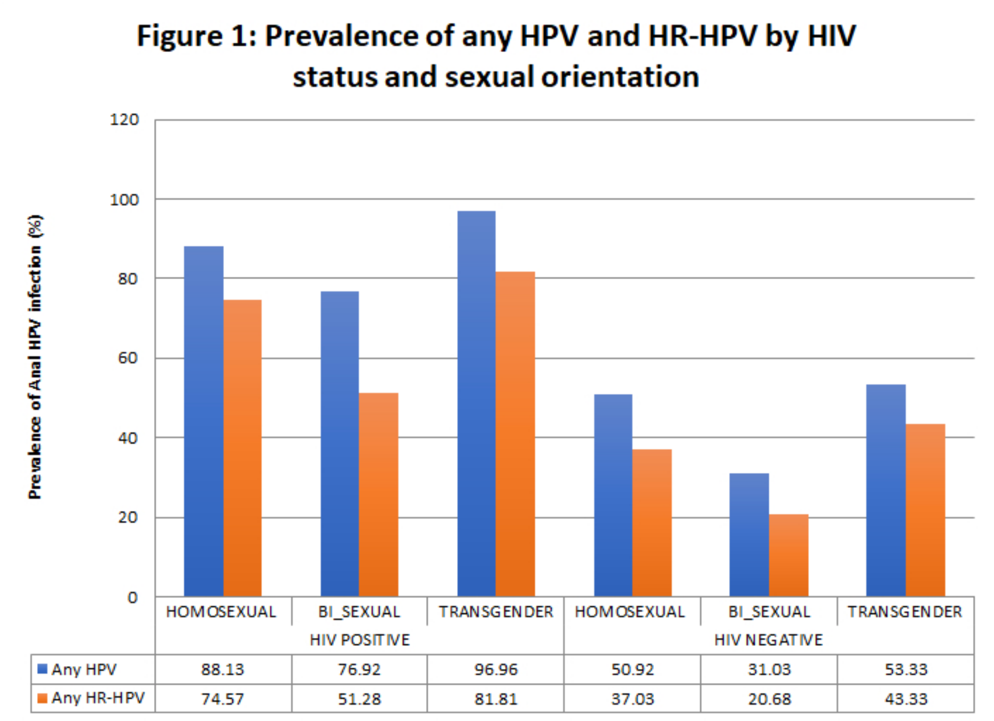

Figure1: Prevalence of any HPV and HR-HPV by HIV status and sexual orientation

187x135mm (96 x 96 DPI)

RESEARCH CHECKLIST

Anal human papillomavirus infection among men who have sex with men and transgender women in Pakistan: Findings from a cross sectional study

Muslima Ejaz, MD MSc[1,2] Sören Andersson MD, PhD[3], Salma Batool PhD[4], Tazeen Saeed Ali PhD[5], Anna Mia Ekström MD MPH PhD[1]

	PAGE NUMBER
Title	1
Abstract	2
**Introduction:**	
Background/rationale	4
Objective	5
**METHODS**	
Study Design	5
Setting	5
Participants	5
Variables	6
Data Sources/measurement	6
Study size	6
Quantitative variables	6
Statistical methods	7
**RESULTS**	
Participants	7
Descriptive data	7
Outcome data	8
Main results	8
**DISCUSSION**	
Key results	9
Limitations	11
Generalisability	11
Interpretation	11
**Other information**	
Contribution	12
Acknowledgement	12
Funding	12
Conflict of interest	12
Presentation of this work	12

For peer review only

Anal human papillomavirus infection among men who have sex with men and transgender women living with and without HIV in Pakistan: Findings from a cross-sectional study

Journal:	*BMJ Open*
Manuscript ID	bmjopen-2021-052176.R3
Article Type:	Original research
Date Submitted by the Author:	13-Oct-2021
Complete List of Authors:	Ejaz, Muslima; Aga Khan University Medical College Pakistan, Community Health Sciences; Karolinska Institutet, Public Health Department<br>Andersson, Soren; Orebro Universitet, Lab Medicine<br>Batool, Salma; Sindh Institute of Urology and Transplantation, Molecular Biology<br>Ali, Tazeen; Aga Khan University Medical College Pakistan, Community Health Sciences & School of Nursing<br>Ekström, Anna; Karolinska Institutet, Department of Public Health Sciences
**Primary Subject Heading**:	Epidemiology
Secondary Subject Heading:	Sexual health, Public health, Infectious diseases, HIV/AIDS, Health policy
Keywords:	Epidemiology < INFECTIOUS DISEASES, HIV & AIDS < INFECTIOUS DISEASES, MOLECULAR BIOLOGY, PUBLIC HEALTH, SEXUAL MEDICINE

I, the Submitting Author has the right to grant and does grant on behalf of all authors of the Work (as defined in the below author licence), an exclusive licence and/or a non-exclusive licence for contributions from authors who are: i) UK Crown employees; ii) where BMJ has agreed a CC-BY licence shall apply, and/or iii) in accordance with the terms applicable for US Federal Government officers or employees acting as part of their official duties; on a worldwide, perpetual, irrevocable, royalty-free basis to BMJ Publishing Group Ltd ("BMJ") its licensees and where the relevant Journal is co-owned by BMJ to the co-owners of the Journal, to publish the Work in this journal and any other BMJ products and to exploit all rights, as set out in our licence.

The Submitting Author accepts and understands that any supply made under these terms is made by BMJ to the Submitting Author unless you are acting as an employee on behalf of your employer or a postgraduate student of an affiliated institution which is paying any applicable article publishing charge ("APC") for Open Access articles. Where the Submitting Author wishes to make the Work available on an Open Access basis (and intends to pay the relevant APC), the terms of reuse of such Open Access shall be governed by a Creative Commons licence – details of these licences and which Creative Commons licence will apply to this Work are set out in our licence referred to above.

Other than as permitted in any relevant BMJ Author's Self Archiving Policies, I confirm this Work has not been accepted for publication elsewhere, is not being considered for publication elsewhere and does not duplicate material already published. I confirm all authors consent to publication of this Work and authorise the granting of this licence.

For peer review only

Anal human papillomavirus infection among men who have sex with men and transgender women living with and without HIV in Pakistan: Findings from a cross-sectional study

Muslima Ejaz, MD MSc[1,2] Sören Andersson MD, PhD[3], Salma Batool PhD[4], Tazeen Saeed Ali PhD[5], Anna Mia Ekström MD MPH PhD[1]

[1]Department of Global Public Health, Karolinska Institutet Stockholm Sweden

[2] Department of Community Health Sciences the Aga Khan University Karachi Pakistan

[3] Department of Laboratory Medicine, Faculty of Medicine and Health, Örebro University, Örebro, Sweden

[4] Department Molecular Biology, Sindh Institute of Urology and Transplantation Karachi Pakistan

[5] School of Nursing, The Aga Khan University Karachi Pakistan

*Corresponding author

Dr. Muslima Ejaz

Doctoral candidate at Department of Global Public Health

Global & Sexual Health Research group (GloSH)

Karolinska Institutet

Stockholm Sweden

Muslima.ejaz@ki.se

Senior Instructor at Department of Community Health Sciences

Aga Khan University (AKU)

Stadium Road, P.O. Box 3500

Karachi 74800, Pakistan

Phone: 92-21-34864886

Muslima.ejaz@aku.edu

For peer review only

ABSTRACT

Objectives: The aim of this study was to determine the prevalence of infection, genotypes and risk-factors for Human Papillomavirus (HPV) among men who have sex with men (MSM) and transgender-women living with and without HIV in Pakistan. Anal infection with HPV is very common worldwide among MSM, particularly among MSM living-with-HIV**.** The high prevalence of HIV among MSM and male-to-female transgendered individuals in Pakistan is a significant health concern since access to screening and health-seeking is often delayed in this stigmatized key population.

Design: This cross-sectional study was conducted between March-2016-to-November-2017.

Participants, setting and data-collection: This study recruited MSM and transgender-women who self-reported to have had anal-sex in the last 6-months, and were at least 18-years-of-age, from the sexual-health and anti-retroviral-therapy centers. Structured questionnaires were administered, and blood-samples were obtained to confirm HIV-status. Anal-swabs were collected for HPV-DNA detection and typing.

Main outcome measures: The primary outcome was the prevalence of "HPV-DNA infection". The prevalence-ratios (PR) were calculated using Cox-proportional-hazard-model algorithms to analyze the association between exposure variables and HPV-infection.

Results: Complete data were available for 298 MSM and transgender-women (HIV+ n=131; HIV- n=167). The overall HPV-DNA prevalence was 65.1% and was higher in participants living-with-HIV as compared to HIV-negative (87%versus48%; χ^2p=<0.001). Likewise, 28.9% of participants living-with-HIV were infected with two or more-than-two types of HPV as compared to 18.8% participants without HIV(χ^2 p=<0.001). The most frequent HPV-type was HPV6/11(46.9%), followed by HPV16(35.1%), HPV18(23.2%), and HPV35(21.1%). HIV-status (**PR:2.81;95%CI 2.16-3.82**) and never-condom-use (**PR:3.08;95%CI 1.69-5.60)**) were independently associated with prevalence of "**Anal-HPV16 infection**" when adjusting for confounding for age, other sexual and behavioral factors e.g., smoking and alcohol consumption.

Conclusion: High prevalence of HPV indicates a substantial future risk of anal cancer in Pakistani MSM and transgender-women, and particularly in those living-with-HIV. Current findings support anal Pap-smear HPV screening for this particular group and vaccination efforts for future generations.

For peer review only - http://bmjopen.bmj.com/site/about/guidelines.xhtml

Strengths and limitations of this study

STRENGTHS

- This study is the first description of the epidemiology of anal HPV infection among sexual and gender minorities (men who have sex with men, and transgender women) living with HIV in Pakistan.
- Given the strong stigma, homophobia and discrimination against sexual minorities in Pakistan, data collection included a careful process of gaining trust among study participants.
- The results may be of a public health importance in Pakistan, indicating the need for gender-neutral HPV vaccination before sexual debut, targeting both women and men.

LIMITATIONS

- The cross-sectional study design precludes any conclusion on temporal relationships between potential risk factors and the outcome.
- Any under- or over-reporting regarding sexual behaviors or lifestyle factors leading to information bias cannot be excluded.

INTRODUCTION:

For peer review only - http://bmjopen.bmj.com/site/about/guidelines.xhtml

Human papillomaviruses (HPV) is the most common sexually transmitted infection (STI) worldwide[1], and is rapidly acquired after sexual debut[2]. A meta-analysis of global data on HPV in men who have sex with men (MSM) found prevalence rates of anal HPV to be 93% in MSM living with HIV and 64% in HIV-negative MSM, and these rates did not decrease with increasing age[3]. More than 90 genotypes of HPV have been identified and classified either as low-risk (LR) types (predominantly HPV6 and HPV11) causing genital warts. High-risk (HR) HPV types (predominantly HPV16 and HPV18) cause cancer of the cervix[4] , its pathogenic role has also been demonstrated by others with oropharyngeal cancers[5,6]. Persistent infection with HPV16 is the major cause of anal cancer[7,8] . Whilst the burden of HPV-related disease in all men is lower than in all women, certain at-risk groups such as MSM and transgender women[9,10], and particularly those with HIV infection[7], are at high risk for HPV infection, and related anal cancer and its precursor high-grade squamous intraepithelial lesions[11]. The reported incidence of anal cancer is 1 – 2/100,000 in the general population[12], while in MSM it has been reported up to 35 cases per 100,000[13] and even up to 131/100,000 in MSM living with HIV[14,15] regardless if they are on antiretroviral treatment or not [16-18].

In Pakistan, the demography of MSM is broadly classified as: ***MSM*** who are in relationships but outwardly closeted; as Male Sex Workers (***MSW)*** who are males and undertakes receptive anal or oral sexual activity with a man in return of money or other financial benefits; and as ***hijras*** (transvestites/transgender women) who are biologically male with male genitals but function in society as if female and take the receptive role in anal sex [19,20]. The "hijra" is a South-Asian identity traced back many centuries to India, where they were held in high-esteem and were political advisors to kings and bodyguards to queens. Today, however, they often face social exclusion and many engage in commercial sex work[21].

Pakistan is an authoritarian Islamic republic with conservative social values, particularly on issues of sex. MSM, and in particular MSW, face high levels of discrimination and contempt from society and are subject to harassment, sexual violence and detention by the state authorities. Their health-seeking behaviors are often poor due to stigmatization in healthcare settings and for the fear of being identified. As a result, they are reluctant to seek health care not only in public set-ups but also in private facilities. One previous study on sex work and MSW in Pakistan showed that the majority of MSWs are poor, have little knowledge about safe sex, and cannot negotiate the use of condoms and safe sex with their paying clients, predisposing them to acquiring STIs[20].

Pakistan's national government has lately recognized MSM as a vulnerable population, and prevention and control programs targeting MSMs in its big cities are being organized (of the estimated 46,264 MSM in Pakistan, Karachi is home to an estimated 18,361 MSM, according a survey conducted for mapping key populations in 2015-2016)[22]. However, program service delivery, such as HIV counseling and testing and

For peer review only - http://bmjopen.bmj.com/site/about/guidelines.xhtml

other STI screening services, is performed by non-governmental and community-based organizations. They are dependent on national and international funders, which has led to fragmented and incomplete coverage of preventive services for this key population.[23,24] Furthermore, the prevalence of HIV in these groups is reaching alarming proportions. Results from the 5th round of Integrated Behavior and Biological second-generation surveillance for HIV/AIDS in Pakistan 2016-17 reported over 9% HIV prevalence in MSM and almost 13% in transgender women living in Karachi[22].

So far there is no research on the burden of HPV infection and HPV-related anal squamous intraepithelial lesions in this key population in Pakistan. Currently, HPV vaccines, in which the bivalent vaccine protects against HPV16/18 and quadrivalent vaccine protects against HPV16/18 and the low-risk types HPV6/11[25], are available in pharmacies in Pakistan but are not yet made available to the target population. The Ministry of Health has not yet included it in their national immunization program for any group in society. We conducted a cross sectional study in Karachi Pakistan in 2016-17 with the objective to determine the prevalence and risk factors for anal HPV infection including sexual risk behaviors and the association with HIV infection in this dynamic population to inform future policy and prevention strategies.

METHODS

Setting and participants:

Enrollment occurred in two phases for this cross-sectional study. The initial recruitment (from March 2016 to May 2016) started at the ART clinic with transgender women, a center run by the National AIDS Control Program of Pakistan in Civil Hospital Karachi (CHK) - the largest (1,900 bed) tertiary care public hospital of Karachi Pakistan (recruited n=33). Low pace, logistic issues and privacy concerns led to the relocation and setting-up of recruitment center at a sexual health clinic run by a community-based organization, Perwaaz Trust Karachi Pakistan, where the study population was reached using snowball technique and peer referrals. During the second phase between April 2017 to November 2017 a total of 265 study participants with different sexual orientations i.e., MSM (homosexuals, bisexuals), and transgender women living with and without HIV were recruited. Participants were eligible for the study who self-reported to have had anal sex in the last preceding at least 6 months and were age 18 and above.

Patient and public Involvement: No patients or member of the public were involved in the design, conduct, reporting, or dissemination plans of our study. Data were kept confidential and anonymous and were used for research purposes only.

Study Procedure: The Ethics Committee on Human Research of Aga Khan (3612-CHS-ERC-15) and Dow (IRB-557/DUHS/APPROVAL/2015/84) Universities of Health Sciences approved the study. Verbal and

For peer review only - http://bmjopen.bmj.com/site/about/guidelines.xhtml

written informed consent were obtained from all eligible and willing participants for all study procedures. For participants who could not read or write their name, a thumb impression was obtained. Participants were informed that they were free to interrupt the interview or withdraw from the study at any time without any negative consequences in terms of clinical services provided to them. A 35-40-minute-long structured questionnaire was administered at a private space to interview the study participants in order to collect data concerning socio-demographic information, sexual and reproductive history and medical history relevant to HIV, any anal disease and use of ART. A blood sample was obtained for confirmation of their HIV status (through Architect HIV Ag-Ab Combo kit by ABBOTT), viral load (Real time PCR Artus Qiagen) and CD4+ T-cell count (through FACS Count TM flow cytometry, Becton Dickinson Co., Franklin Lakes, NJ, USA). Two anal swabs for HPV testing were collected by a trained general physician. A water-moistened swab was inserted 5 – 6 cm proximal to the anal verge and the samples were obtained from anorectal transition zone around the dentate line in the anal canal. The swab was then agitated vigorously in a tube containing 3 mL of methanol-based fixative - a Sample Transport Medium (UTM-RT viral transport media with flocked polyester swabs, Copan Diagnostics Inc., Corona, CA) and transported to the lab. In Molecular lab the solution was stored at -70 °C before further processing and was later used for the extraction and detection of HPV DNA by the polymerase chain reaction (PCR). The DNA from rectal swab was extracted by using Qiagen DNA mini kit and DNA was eluted in 80 ul of elution buffer and stored at -80 °C for further use. HPV DNA was detected by using MY09/MY11 primers from L1 region resulted in amplification of 450 bp of L1 gene. PCR for Housekeeping gene Glyceraldehyde 3-phosphate dehydrogenase (GAPDH) was done for every extracted DNA and samples tested negative for GAPDH gene were excluded from study. Nested Multiplex PCR (NMPCR) assay was used [26] for detection HPV subtypes included low risk types 16/11 and high-risk (group 1) types 16, 18, 31, 33, 35,52,56,58 and 59 as described by Sotlar et al[27]. Primers for first-round PCR for GP-E6/E7 consensus sequence and second round PCR for HPV subtypes 16, 18, 16/11 31, 33, 35,52,56,58 and 59 were synthesized from Eurofins MWG/Operon Germany. The HPV subtype specific primers were used in four cocktails and size of the nested PCR product was used for the identification of each HPV type by gel electrophoresis[28].

Sample size calculation:

For the estimation of HPV prevalence, a sample size of 267 participants were needed with an anticipated prevalence of HPV of 50%, 6% precision and 5% level of significance. For the analysis of factors associated with HPV infection**,** we aimed for a sample of at least 256 participants to achieve 80% power with a range

For peer review only - http://bmjopen.bmj.com/site/about/guidelines.xhtml

of exposures between 9% to 39% and an anticipated prevalence ratio of 2.5 or more with 5% level of significance. An additional sample of 10% was added to cater for inadequate biological samples.

Data management and Statistical Analysis

The database was analyzed using Statistical Package for Social Sciences (IBM SPSS Statistics for Windows, Version 24.0. Armonk, NY: IBM Corp).

The mean value was calculated with standard deviation for symmetrically distributed variables and independent sample t-test was used for statistical significance, while the median with interquartile ranges was used for asymmetrical quantitative variables. For categorical variables, proportions were employed; for comparison of proportions between groups, $\chi 2$ or Fisher Exact test (where needed) were used for p-values. The overall HPV DNA prevalence (as determined by PCR) is reported as the proportion of HPV positive participants among all included individuals. Conversely, type-specific HPV positivity is presented as the proportion of HPV- subtype positive individuals divided by all HPV positive individuals[7]. The confidence intervals for the proportions were estimated using binomial distribution (n,p) assuming a Gaussian error. Prevalence of “Any anal HPV infection” and “Any anal HR-HPV infection” stratified by HIV status for all reported sexual orientations was also assessed using Pearson $\chi 2$. For multiple comparisons for each sexual orientation stratified by HIV status for “Any anal HPV infection” and “Any anal HR-HPV infection” Bonferroni Correction Method was used, the p-value threshold was $(\alpha/n) = 0.05/6 = 0.0083$.

Since the design of the study was cross sectional and the outcome of the interest was common (HPV prevalence); for the association between independent risk factors and the outcome variables “any anal HPV infection” i.e., all those HPV types that we identified in our study sample and “anal HPV**16** infection”, we employed a Cox proportion hazard model algorithm to estimate crude and adjusted prevalence ratios (PRs) and their 95% confidence intervals (CIs) in univariate and multivariate models’. Moreover, we employed robust SEs to get the closest CIs. A significance threshold for the *p value* <0.05 was used for the final model.

RESULTS:

Study Population Characteristics: The final analysis included 298 MSM and transgender women (235 MSM and 63 transgender women) to assess the prevalence of HPV DNA in the anal canal. Out of these 298, 131 (44%) had an HIV-infection and 167 (56%) were HIV-negative. Among MSM living with HIV, 33 (25%) were transgender women. Almost 90% of the participants living with HIV were on ART and two thirds of them had an undetectable HIV viral load.

For peer review only - http://bmjopen.bmj.com/site/about/guidelines.xhtml

The overall mean age (±SD) of the 298 recruited participants was 28.8 (±8.06). The level of education was low: 28% reported no formal education and 12% less than primary level. Most respondents were currently not married. Among study participants almost 78% were smokers with mean age of smoking initiation 16.5 (±4.9) and duration of 12 (±8.5) years. Almost 80% were sex workers, with 12.4 (±8.5) years mean duration of sex work. The mean age at sexual debut was 16.4 (±4.7) years. Only 24.2% reported consistent condom use during sex (Table 1).

MSM with any anal HPV infection (n=194) were younger than MSM without detectable HPV infection (28.9 (±7.3) versus 30.2 (±9.1); independent sample t-test p-value = 0.027). Smoking was more prevalent among HPV-positive MSM (82.5% versus 69.2%) than among those uninfected, with a significant difference in mean age (±SD) of 15.6 (±4.5) at smoking initiation. The mean age (±SD) at sexual debut among study participants with an HPV infection was 15.7 (±4.3) versus 17.6 (±5.2) (independent sample t-test-p= 0.001). HPV-infected had more sexual partners, more inconsistent condom users and more often practiced receptive anal sex. (Table 1).

MSM living with HIV were, however, older than MSM who were HIV negative (mean (±SD): 30.9 (±0.70) versus 27.2(±0.59) independent sample t-test p=<0.001), more frequently smokers (χ2p=0.01), younger at initiation of smoking (independent sample t-test p=<0.001) and had smoked for a longer duration of time ((χ2p=<0.001). They also reported younger age at sexual debut (independent sample t-test p=<0.001), had more sexual clients (χ2p=<0.001), were mainly the receptive anal partner (χ2p=<0.001), and used condoms less consistently than their HIV negative counterparts (χ2p=<0.001).

Sexual orientation of the study sample:

Among participants living with HIV, 45.1% reported their sexual orientation as homosexual, 29.8% as bisexuals, and 25.1% as transgender women. The sexual orientations reported by HIV uninfected participants were, 64.7% as homosexuals, 17.3% as bisexuals and 18.0 % as transgender women.

HPV DNA Prevalence and Genotype Distribution

Overall, 65.1% (n=194/298) of the total study population was positive for HPV DNA (95% CI: 59.5-70.3%). MSM living with HIV had a significantly higher prevalence of HPV (87% (80.0-91.0%)) compared to those who did not have HIV (48% (40.0-55.0%)) as well as any oncogenic anal HPV infection (83.2% (75.8-88.6%) verses (35.3% (28.4-42.8%). Likewise, 28.9% of participants living with HIV were infected with two or more-than-two types of HPV as compared to 18.8% participants without HIV ((χ2p=<0.001). The most common HPV types were HPV6/11 (46.9%), HPV16 (35.1%), HPV18 (23.2%), HPV35 (21.1%), and HPV58 (8.2%). Moreover, the distribution of HPV6/11 was more among HIV uninfected participants (61.3%) as

For peer review only - http://bmjopen.bmj.com/site/about/guidelines.xhtml

compared to HIV infected participants (36.8%) ((χ2p-value;0.001). However, HPV16 was more common among HIV infected participants (44.7% verses 21.2%) and the difference was statistically significant ((χ2 p-value; 0.001) (Table 2)

HPV DNA prevalence by HIV status and sexual orientation:

For **participants living with HIV,** the prevalence of "Any anal HPV infection" were 88.1 % for homosexual men, 76.9% for bisexual men and 96.9% for transgender women, and the prevalence for "Any anal HR-HPV infection" was 74.5%, 51.3% and 81.8% respectively. ((χ2p=0.001) (Figure 1)

For **HIV uninfected participants**, the prevalence of "Any anal HPV infection" was 50.9 % for homosexual men, 31.0% for bisexual men and 53.4% for transgender women, and the prevalence for "Any anal HR-HPV infection" was 37.1%, 20.7% and 43.3% respectively. ((χ2p=0.001)

Post-hoc analysis with the Bonferroni Correction Method determined that the homosexuals were significantly different with being infected with "Any anal HPV infection" and "Any anal HR-HPV infection" ((χ2p-values: 0.0014 and 0.0019) when compared to bisexuals and transgender women living with and without HIV

Independent variables associated with anal HPV infection

Being HIV positive (PR 2.81 (95% CI 2.16-3.82) and inconsistent/never condom use (PR 2.61 (1.50-4.50) / (PR 3.08 (1.69-5.60) were independent risk factors associated with **increased prevalence of HPV16 infection**, adjusted for all other demographic, sexual and behavioral risk factors (Table 3).

DISCUSSION

To the best of our knowledge, this is the first report on anal HPV infection among MSM living with and without HIV in Pakistan. The high prevalence of anal HPV among MSM living with HIV (87%) in Pakistan is similar to Thailand (85.0%)[29] and China (81% - 82%) [30,31], slightly lower than in neighboring India (95%)[32] and Canada (98%)[33], but significantly higher than what has reported from the Netherlands (65%) [34]. In HIV-negative MSM, however, studies previously carried out in the United States and Latin America showed similar prevalence levels of HPV prevalence ranging from 42% to 66%, which is comparable to our 48% [35-37]. In Pakistan, there is heterogeneity among MSMs, and it is predominantly MSWs who register with the community-based organizations with a hope to get free medical services of any kind in a secluded setting. It is MSW who accounted for 80% of our study population, partly explaining the differences in the prevalence of anal HPV infection between our study and others. In a study from India, which has similar

For peer review only

social and cultural values, Hernandez et al [32] noticed HPV prevalence of 98% in MSM living with HIV and commented on the fluid nature of sexual identity without mentioning the exact prevalence of MSW among them. Moreover, given the fact that our study has a cross-sectional design, incubation time for infection and variation in viral clearance could be other possible explanations for the differences in the prevalence.[29,38]

For the distribution of anal HPV types, our study results are similar to the studies done elsewhere[39]. HPV16 (35.1%) was the most common anal oncogenic HPV type detected both in MSM living with and without HIV (44.7% versus 21.2%, p <0.001). This is in close approximation of the summary prevalence for HPV16 calculated from a recent meta-analyses[7,40] and by others [29,33,41,42]. HPV16 has the ability to persist in the anal canal more than any other types and not only tends to cause AIN [29] but also associated with disease severity[7].

The prevalence of HR-HPV type 35 (21.1%) in our study was similar to the levels reported by India (20%)[32], but higher than HPV type 35 levels reported from other parts of the world [31-33]. Given the fact that HPV35 is not included in the FDA-approved 9-valent vaccine, its relatively high prevalence can have implications on anal cancer prevention in our region.

Similar to previous research [43], our results also revealed that almost 4 out of 5 MSM carried at least one of the high-risk genotypes in their anal canal (80% MSM living with HIV versus 73% of MSM without HIV infection). This is of concern in HIV-negative MSM in particular, since infection with HR-HPV types is reported to increase the likelihood of acquiring HIV[44]. Thus, our results further emphasize the need to screen Pakistani MSM more frequently for both HPV and HIV given their high risk of development of malignant disease.

Our study detected a high prevalence of multiple HPV types – i.e., at least 2 types (either low-risk and high risk or high-risk & high-risk) – of 47% in HPV positive MSM as a group, which is similar to other, studies [45]. This indeed is a matter of concern as anal infection with multiple HR-HPV types increases the likelihood of an individual to be infected with HPV16 and, infection with HPV16 has been associated with AIN and progression to higher grade AIN over time[3]. Moreover, its prevalence was significantly higher in MSM living with HIV. Higher HPV reinfection rates and lower clearance rates in individuals living with HIV may also play a crucial role in the development and the progression of anal neoplastic disease[29,46].

The results of our study suggest that the most frequently distributed HPV types among Pakistani MSM could be covered by the currently available nonavalent (93.3%) and quadrivalent (84%) vaccines, which means that most of HPV-related diseases including cancers and anogenital warts may potentially be

For peer review only - http://bmjopen.bmj.com/site/about/guidelines.xhtml

preventable if offered to MSM at initiation of sexual debut. However, the 20% carrying HPV35 would not be covered by the currently available vaccines.

More than 50% of HPV-positive MSM were young, below 25 years of age, most were single and sexually very active, and most make a living and support their families through sex work[47]. As many as 80% of HPV-positive MSM had initiated sex work in their teens, which is in line with other studies[48]. The high number of parallel sexual partners may contribute to further spread of HPV and other STIs among MSM.

Among other sexual behaviors, inconsistent or never use of condom was the only variable that was significantly associated with any HPV, specifically HPV16 infection. For many in our study population, negotiating safe sex is not an option as, on an average, a male sex worker will service 3 to 5 clients per night with a one-time service charge as low as 100 – 150 Pakistani rupees (US$ 0.7 to 1.03)[19 49], though higher rates may be charged for unprotected sex. Thus, financial barriers may impede consistency in condom use. In addition, receptive anal sex was significantly more common among HPV-infected MSM, considering the fact that this is the strong transmission route and poses high risk for HIV acquisition[50]. Since the prevalence of anal HPV was more than twice as common in MSM and transgender women living with HIV, this finding indeed turned out to be consistent with our multiple regression model that identified HIV infection as an independent risk factor for any anal HPV or HPV16 infection confirming MSM and transgender women living with HIV as a priority for the prevention of anal cancer[40] .

Limitations of the study: Our study had some limitations. First, the design of the study was cross-sectional, yet many HPV infections are non-persistent in nature[51]. Moreover, the cross-sectional design precludes us to determine a temporal relationship, however the risk factors identified in our study appear robust and have also been found in studies from other countries. Second, our study results may have limited generalizability because participants were recruited from only one city of Pakistan (Karachi) and were recruited using peer referrals and snowball sampling techniques. Nevertheless, Karachi is the largest metropolitan city of Pakistan, representing all ethnicities of the country. Moreover, in a country like Pakistan, which is by and large a heteronormative society and where homophobia prevails against this sexual minority due to cultural and religious inhibitions, using above mentioned sampling technique was the most feasible option for the recruitment process. Third, we cannot exclude the possibility of under- or over-reporting of sexual behaviors and lifestyle factors that could not only influence the strength of association with the outcome but might also lead to information bias since they were self-reported.

For peer review only - http://bmjopen.bmj.com/site/about/guidelines.xhtml

Recommendations: HIV care and treatment programs in Pakistan represent a potential window of opportunity for providing preventive clinical interventions to avert an anal cancer epidemic nested within the HIV epidemic. Providing cost-effective, life-saving anal cancer prevention services (i.e., screen and treat approach and vaccination) linked to HIV care could not only avert preventable HPV-related malignancies but could also lead to a more efficient utilization of resources and improve health care access for those most at risk. Moreover, our study findings emphasize the need for government support of interventions that can improve access to and quality of services to MSM including MSW. Prevalent cultural and social stigma must also be addressed since this is essential for protecting the dignity and autonomy of this population.

Conclusions: We believe that our study findings are of importance for clinical practice and public health policy. The relatively high prevalence of high-risk HPV indicates a future risk of anal cancer among MSM in general and among MSM living with HIV in particular. Given that HPV35 currently is not included in the 9-valent HPV vaccine, we strongly feel that this is one of our most important contributions and that there is a need for regular anal pap screening among MSM and transgender women to enable early detection and treatment to prevent anal cancer.

Contributors: ME, SB conceptualized the study. ME designed the study. AME, SA & TSA supervised the main study implementation. Lab work was supervised by SB & ME. ME did the data collection, data cleaing and mining. ME did the data analysis. ME prepared the first draft of the manuscript with initial revisison from SA and TSA. AME did the extensive revisions of the manuscript with ME. All authors reviewed and approved the final version of the manuscript.

Acknowledgements: We thank our all study participants and the staff at our study sites for their continuous cooperation and collaboration. We are enormously thankful to the Sindh Institute of Urology and Transplantation (SIUT) for their lab supplies (reagents and the sample collection kits) and contribution to the lab analyses. We are grateful to the staff at the histopathology and molecular laboratories of SIUT who devoted their time and efforts to this project (in-kind). We are also grateful for the financial contribution from the Dean's Fund at The Aga Khan Hospital & Medical University which covered the cost of samples transportation to the SIUT labs from the two study sites. Special thanks to Dr Asli Kulane for her encouragement and guidance to conduct this study. Thanks to Ms Shazia for her assistance in the conduct of the lab work. Special thanks to our English Editor Ms Carla Sturm for reviewing the manuscript for typographical/ grammatical errors.

Funding Information: Organisation number: 202100-2973; Vat number: SE202100297301.

For peer review only - http://bmjopen.bmj.com/site/about/guidelines.xhtml

Potential conflict of interest: All authors: No potential conflicts of interest

Data availability Statement: The data that support the findings of this study are available from Global & Sexual Health Research Group of Department of Global Public Health on reasonable request and with permission of Karolinska Institutet (KI) Stockholm Sweden

Presentation of this work: This work has been presented in 32nd International Papillomavirus Conference in conjunction with AOGIN 2018, being held in Sydney Australia from October 2-6,2018. This conference attendance was part of my Doctoral Program and financially being supported by Faculty Development Award by The Aga Khan Hospital & Medical University Pakistan.

Acceptance of the work: This work has ben accepted in STI and HIV 2019 World Congress (13th to 17th july 2019 at Vancouver Canada).

For peer review only

For peer review only - http://bmjopen.bmj.com/site/about/guidelines.xhtml

REFERENCES:

1. Sehnal B, Rozsypal H, Nipčová M, Sláma J. The prevalence, incidence, persistence and transmission ways of human papillomavirus infection (HPV). *Epidemiology.* 2017;66(4):198-209.
2. Zhang L, Regan DG, Ong JJ, et al. Targeted human papillomavirus vaccination for young men who have sex with men in Australia yields significant population benefits and is cost-effective. *Vaccine.* 2017;35(37):4923-4929.
3. Machalek DA, Poynten M, Jin F, et al. Anal human papillomavirus infection and associated neoplastic lesions in men who have sex with men: a systematic review and meta-analysis. *Lancet Oncol.* 2012;13(5):487-500.
4. Fani M, Mahmoodi P, Emadzadeh M, et al. Correlation of human papillomavirus 16 and 18 with cervical cancer and their diagnosis methods in Iranian women: A systematic review and meta-analysis. *Current problems in cancer.* 2020;44(1):100493.
5. Rapado-González Ó, Martínez-Reglero C, Salgado-Barreira Á, et al. Association of Salivary Human Papillomavirus Infection and Oral and Oropharyngeal Cancer: A Meta-Analysis. *Journal of clinical medicine.* 2020;9(5):1305.
6. Nogues JC, Fassas S, Mulcahy C, Zapanta PE. Human papillomavirus-associated head and neck cancer. *The Journal of the American Board of Family Medicine.* 2021;34(4):832-837.
7. Lin C, Franceschi S, Clifford GM. Human papillomavirus types from infection to cancer in the anus, according to sex and HIV status: a systematic review and meta-analysis. *The Lancet Infectious Diseases.* 2018;18(2):198-206.
8. International Agency for Research on Cancer. IARC Monograph on the Evaluation of Carcinogenic Risks to Humans. Biological agents. A review of human carcinogens. *IARC Monogr Eval Carcinog Risks Hum* 2012;100 (B) (1-441).
9. Kobayashi T, Sigel K, Gaisa M. Prevalence of Anal Dysplasia in HIV-infected Transgender Women. *Sexually transmitted diseases.* 2017;44(11):714.
10. Cranston RD, Carballo-Diéguez A, Gundacker H, et al. Prevalence and determinants of anal human papillomavirus infection in men who have sex with men and transgender women. *International journal of STD.* 2019;30(2):154-162.
11. Somia IKA, Teeratakulpisarn N, Jeo WS, et al. Prevalence of and risk factors for anal high-risk HPV among HIV-negative and HIV-positive MSM and transgender women in three countries at South-East Asia. *Medicine.* 2018;97(10).
12. Siegel R, Naishadham D, Jemal A. Cancer statistics, 2012.CA. *Cancer J Clin.* 2012;62:10–29.
13. Jemal A, Murray T, Samuels A, Ghafoor A, Ward E, Thun MJ. Cancer statistics, 2003. *CA Cancer J Clin.* 2003;53(1):5-26.
14. Silverberg MJ, Lau B, Justice AC, et al. Risk of anal cancer in HIV-infected and HIV-uninfected individuals in North America. *Clin Infect Dis.* 2012;54(7):1026-1034.
15. Patel P, Hanson DL, Sullivan PS, et al. Incidence of types of cancer among HIV-infected persons compared with the general population in the United States, 1992-2003. *Ann Intern Med.* 2008;148(10):728-736.
16. Houlihan CF, Larke NL, Watson-Jones D, et al. Human papillomavirus infection and increased risk of HIV acquisition. A systematic review and meta-analysis. *AIDS.* 2012;26(17):2211-2222.

For peer review only - http://bmjopen.bmj.com/site/about/guidelines.xhtml

17. Lawton MD, Nathan M, Asboe D. HPV vaccination to prevent anal cancer in men who have sex with men. *Sex Transm Infect.* 2013;89(5):342-343.
18. Hernandez AL, Efird JT, Holly EA, Berry JM, Jay N, Palefsky JM. Incidence of and risk factors for type-specific anal human papillomavirus infection among HIV-positive MSM. *AIDS.* 2014;28(9):1341-1349.
19. Rajabali A, Khan S, Warraich HJ, Khanani MR, Ali SH. HIV and homosexuality in Pakistan. *The Lancet infectious diseases.* 2008;8(8):511-515.
20. Shaw SY, Emmanuel F, Adrien A, et al. The descriptive epidemiology of male sex workers in Pakistan: a biological and behavioural examination. *Sexually transmitted infections.* 2011;87(1):73-80.
21. Abdullah MA, Basharat Z, Kamal B, et al. Is social exclusion pushing the Pakistani Hijras (Transgenders) towards commercial sex work? A qualitative study. *BMC international health and human rights.* 2012;12(1):32.
22. Second-generation HIV surveillance in Pakistan round 5: Integrated Behavior and Biological Survaillance in Pakistan 2016-17. 2016-2017.
23. Mayhew S, Collumbien M, Qureshi A, et al. Protecting the unprotected: mixed-method research on drug use, sex work and rights in Pakistan's fight against HIV/AIDS. *Sexually transmitted infections.* 2009;85(Suppl 2):ii31-ii36.
24. Buse K, Lalji N, Mayhew S, Imran M, Hawkes S. Political feasibility of scaling-up five evidence-informed HIV interventions in Pakistan: a policy analysis. *Sexually Transmitted Infections.* 2009;85(Suppl 2):ii37-ii42.
25. Schiller JT, Castellsague X, Garland SM. A review of clinical trials of human papillomavirus prophylactic vaccines. *Vaccine.* 2012;30 Suppl 5:F123-138.
26. Essaada Belglaiaa, Hicham Elannaz, Bouchra Mouaouya, et al. Human papillomavirus genotypes among women with or without HIV infection: an epidemiological study of Moroccan women from the Souss area. *Infectious agents and cancer.* 2015;10(44).
27. Sotlar K, Diemer D, Dethleffs A, et al. Detection and typing of human papillomavirus by e6 nested multiplex PCR. *Journal of clinical microbiology.* 2004;42(7):3176-3184.
28. Kiani SJ, Malekshahi SS, Ghalejoogh ZY, et al. Detection and typing of human papilloma viruses by nested multiplex polymerase chain reaction assay in cervical cancer. *Jundishapur journal of microbiology.* 2015;8(12).
29. Phanuphak N, Teeratakulpisarn N, Pankam T, et al. Anal human papillomavirus infection among Thai men who have sex with men with and without HIV infection: prevalence, incidence, and persistence. *J Acquir Immune Defic Syndr.* 2013;63(4):472-479.
30. Ren X, Ke W, Zheng H, et al. Human Papillomavirus Positivity in the Anal Canal in HIV-Infected and HIV-Uninfected Men Who Have Anal Sex with Men in Guangzhou, China: Implication for Anal Exams and Early Vaccination. *Biomed Res Int.* 2017;2017:2641259.
31. Li X, Li M, Yang Y, et al. Anal HPV/HIV co-infection among Men Who Have Sex with Men: a cross-sectional survey from three cities in China. *Sci Rep.* 2016;6:21368.
32. Hernandez AL, Karthik R, Sivasubramanian M, et al. Prevalence of Anal HPV Infection Among HIV-Positive Men Who Have Sex With Men in India. *J Acquir Immune Defic Syndr.* 2016;71(4):437-443.

For peer review only - http://bmjopen.bmj.com/site/about/guidelines.xhtml

33. de Pokomandy A, Rouleau D, Ghattas G, et al. Prevalence, clearance, and incidence of anal human papillomavirus infection in HIV-infected men: the HIPVIRG cohort study. *J Infect Dis.* 2009;199(7):965-973.

34. Auvert B, Lissouba P, Cutler E, Zarca K, Puren A, Taljaard D. Association of oncogenic and nononcogenic human papillomavirus with HIV incidence. *J Acquir Immune Defic Syndr.* 2010;53(1):111-116.

35. Critchlow CW, Hawes SE, Kuypers JM, et al. Effect of HIV infection on the natural history of anal human papillomavirus infection. *AIDS.* 1998;12(10):1177-1184.

36. Palefsky JM, Holly EA, Ralston ML, Jay N. Prevalence and risk factors for human papillomavirus infection of the anal canal in human immunodeficiency virus (HIV)-positive and HIV-negative homosexual men. *J Infect Dis.* 1998;177:361-367.

37. Goldstone S, Palefsky JM, Giuliano AR, et al. Prevalence of and risk factors for human papillomavirus (HPV) infection among HIV-seronegative men who have sex with men. *J Infect Dis.* 2011;203(1):66-74.

38. Dona MG, Vescio MF, Latini A, et al. Anal human papillomavirus in HIV-uninfected men who have sex with men: incidence and clearance rates, duration of infection, and risk factors. *Clin Microbiol Infect.* 2016;22(12): 1004. e1-1004. e7.

39. Liu X, Lin H, Chen X, et al. Prevalence and genotypes of anal human papillomavirus infection among HIV-positive vs. HIV-negative men in Taizhou, China. *Epidemiology Infection.* 2019;147.

40. Marra E, Lin C, Clifford GM. Type-specific anal human papillomavirus prevalence among men, according to sexual preference and HIV status: a systematic literature review and meta-analysis. *The Journal of infectious Diseases.* 2019;219(4):590-598.

41. Sahasrabuddhe VV, Castle PE, Follansbee S, et al. Human papillomavirus genotype attribution and estimation of preventable fraction of anal intraepithelial neoplasia cases among HIV-infected men who have sex with men. *J Infect Dis.* 2013;207(3):392-401.

42. Phanuphak N, Teeratakulpisarn N, Triratanachat S, et al. High prevalence and incidence of high-grade anal intraepithelial neoplasia among young Thai men who have sex with men with and without HIV. *AIDS.* 2013;27(11):1753-1762.

43. Blas MM, Brown B, Menacho L, Alva IE, Silva-Santisteban A, Carcamo C. HPV Prevalence in Multiple Anatomical Sites among Men Who Have Sex with Men in Peru. *PLoS One.* 2015;10(10):e0139524.

44. Houlihan CF, Larke NL, Watson-Jones D, et al. HPV infection and increased risk of HIV acquisition. A systematic review and meta-analysis. *AIDS.* 2012;26(17).

45. van Aar F, Mooij SH, van der Sande MA, et al. Anal and penile high-risk human papillomavirus prevalence in HIV-negative and HIV-infected MSM. *AIDS.* 2013;27(18):2921-2931.

46. Tobian AA, Kigozi G, Gravitt PE, et al. Human papillomavirus incidence and clearance among HIV-positive and HIV-negative men in sub-Saharan Africa. *AIDS.* 2012;26(12):1555-1565.

47. Baral SD, Friedman MR, Geibel S, et al. Male sex workers: practices, contexts, and vulnerabilities for HIV acquisition and transmission. *The Lancet.* 2015;385(9964):260-273.

For peer review only - http://bmjopen.bmj.com/site/about/guidelines.xhtml

493 48. Harrison A, Cleland J, Gouws E, Frohlich J. Early sexual debut among young men in rural South Africa: heightened vulnerability to sexual risk? *Sexually transmitted infections.* 2005;81(3):259-261.

496 49. Noman ul Haq, Sohail Riaz, Aqeel Nasim, Tahir. M. The transgender prostitution: Threat to the rise of AIDS in Pakistan. *Journal of Infection and Public Health.* 2018;11:448.

498 50. Winkelstein W, Lyman DM, Padian N, et al. Sexual practices and risk of infection by the human immunodeficiency virus: the San Francisco Men's Health Study. *JAMA.* 1987;257(3):321-325.

501 51. Alizon S, Murall CL, Bravo IG. Why human papillomavirus acute infections matter. *Viruses.* 2017;9(10):293.

For peer review only

Table 1: Socio-demographic and lifestyle characteristics of MSM and transgender women in Karachi, Pakistan by HPV status

Characteristics	Overall (n=298)	HPV POSITIVE (n=194)	HPV NEGATIVE (n=104)	P- value#
**Age categories in years n (%)**				
< 25	102(34.2)	72 (37.1)	30 (28.8)	
25 – 29	90 (30.2)	59 (30.4)	31 (29.8)	0.254
30 – 34	41(13.8)	27 (13.9)	14 (13.5)	
>35	65(21.8)	36 (18.6)	29 (27.9)	
**Education n (%)**				
None	83(27.9)	56 (29.2)	27 (26.2)	0.334
Primary school	36 (12.1)	29 (15.1)	07 (6.8)	
Middle school	41 (13.8)	24 (12.5)	17 (16.5)	
Matric school	78 (26.2)	49 (25.5)	29 (28.2)	
Intermediate	38 (12.8)	23 (12.0)	15 (14.6)	
Graduate	19 (6.4)	11 (5.7)	08 (7.8)	
**Marital Status n (%)**				
Unmarried	234 (78.5)	156 (80.4)	78 (75.0)	
Married	57 (19.1)	32 (16.5)	25 (24.0)	0.256
Separated/divorced	7 (2.3)	06 (3.1)	01 (1.0)	
**Smoking status n (%)**				
Yes	232 (77.9)	160(82.5)	72(69.2)	0.009
**Average number of cigarettes/day n (%)**				
<10	128 (55.2)	82 (52.9)	46(63.9)	0.035
10-20	79 (34.1)	52 (33.5)	24(33.3)	
>20	25 (10.7)	21 (13.5)	2(2.8)	
**Alcohol drinking: n (%)**				
Yes	71 (23.8)	44 (23.4)	37 (20.4)	0.555
**Sexual behavior: n (%)**				
Bisexual	68 (22.8)	39 (20.1)	29 (27.9)	0.127
Homosexual	167 (56.0)	108 (54.6)	59 (56.71)	
Transgender	63 (21.2)	47 (25.3)	16 (15.4)	
**Male Sex Workers: n (%)**	237 (79.5)	155 (79.5)	82 (78.8)	0.830
**Anal receptive sexual partners during the last 6 months* n (%)**				
<25	25 (8.4)	16 (8.7)	9 (10.2)	0.004
25 – 50	36 (12.1)	17 (9.3)	19 (21.6)	
51 – 100	89 (29.9)	56 (30.6)	33 (37.5)	
>100	121 (40.6)	94 (51.4)	27 (30.7)	
**Condom use during anal receptive sex n (%)**				
Consistent use	72 (24.2)	15 (7.7)	49 (47.1)	<0.001
Inconsistent use	175 (58.7)	141 (72.7)	41 (39.4)	
Never	51 (17.1)	38 (19.6)	14 (13.5)	
**Preferred Anal sex role n (%)**				
Mainly receptive	264 (88.6)	173(89.2)	81 (77.9)	0.009
Mainly insertive	34 (11.4)	21 (10.8)	23 (22.1)	
**Other STI (gonorrhea, Trichomonas) n (%)**	167 (56.0)	133(68.6)	34(32.7)	<0.001
**Recruitment settings n(%)**				
**ART center**	33 (11)	20(10.3)	13(12.5)	0.566
**CBO**	265(89)	174(89.7)	91(87.5)	

*Missing values = 9.1%

#($\chi 2$ *test was used for* $p = values$)

For peer review only

For peer review only - http://bmjopen.bmj.com/site/about/guidelines.xhtml

Table 2: Distribution of HPV types among MSM and transgender women in Karachi, Pakistan stratified by HIV Status

HPV TYPES	ALL	HPV among HIV Positive	HPV among HIV negative	P-value##
	**n (%)**	**n (%)**	**n (%)**	**p-value**
**Any type**	194 (65.1)	**114** (58.8) 87.0	**80** (41.2) 48.0	<0.0001
**High risk types**				
HPV 16	68 (35.1)	51 (44.7)	17 (21.2)	0.001
HPV 18	45 (23.2)	30 (26.3)	15 (18.8)	0.219
HPV 31	14 (7.2)	8 (7.5)	6 (7.0)	0.898
HPV 33	13 (6.7)	13 (11.4)	0 (0.0)	NC
HPV 35	41 (21.1)	25 (21.9)	16 (20.0)	0.746
HPV 45	14 (7.2)	6 (5.3)	9 (11.3)	0.124
HPV 52	13 (6.7)	4 (3.5)	9 (11.3)	0.043*
HPV 58	16 (8.2)	13 (11.4)	3 (3.8)	0.066*
HPV 59	21 (10.8)	10 (8.8)	11 (13.8)	0.272
HPV 56	15 (7.7)	8 (7.0)	7 (8.8)	0.657
**Low risk types**				
HPV 6/11	91 (46.9)	42 (36.8)	49 (61.3)	0.001
**VACCINE SPECIFIC TYPES**				
**Bivalent vaccine type** HPV 16 & /18	107 (55.2)	75 (65.8)	32 (40.0)	0.000
**4-valent vaccine type** HPV 6/11/16/18	163 (84.0)	94 (82.4)	69 (86.2)	0.478
****9-valent vaccine type** HPV 6/11/16/18/31/33/45/52/58	181 (93.3) #	105 (92.1)	76 (95.0)	0.427

NC: Not calculated, one of the cell counts was 0
*Fisher's Exact Test
** Includes 9-valent all types which come as alone, in combination with other 9-valent specific types or in combination with non-9-valent HR types such as HPV type 35, when we excluded HPV 35 (n=33)
# 93.3% changed to 76.3%
##($\chi 2$ test was used for $p = values$)

For peer review only

Table 3: factors associated with any type of detectable HPV infection, and HPV type 16, among MSM and transgender women in Karachi Pakistan (N=298)

Risk factors	HPV positive N (%)	Any Anal HPV infection Crude PR[#] (95% CI)	Any Anal HPV infection Adjusted PR (95% CI)	Anal HPV 16 - infection Crude PR (95% CI)	Anal HPV 16 - infection Adjusted PR (95% CI)
**HIV status**					
No (ref)	80 (48.0)		**1.0**	1.0	**1.0**
Yes	114 (87.0)	1.81 (1.36-2.4)	**1.44 (1.05-1.97)**	3.33(2.55-3.51)	**2.81 (2.16-3.82)**
**Other STI****					
No (ref)	61 (31.4)	1.0	**1.0**	1.0	1.0
yes	133 (68.6.0)	1.32 (1.08-2.13)	**1.29 (1.02-1.91)**	1.97 (1.28-3.04)	1.52 (0.99-2.37)
**Preferred anal sex role**					
Insertive (ref)	21 (10.8)	1.0	1.0	1.0	
Receptive	173 (89.2)	1.95 (1.09-3.50)	1.56 (0.87-2.83)	2.49 (1.02-6.14)	
**Condom use**					
Consistent (ref)	15 (7.7)	1.0	1.0	1.0	**1.0**
Inconsistent	141 (72.7)	3.30 (1.94-5.6)	**2. 01 (1.07-5.20)**	2.96 (1.48-5.92)	**2.61 (1.50-4.50)**
never	38 (19.6)	2.42 (1.04-5.21)	**1.58 (1.03-4.44)**	3.11 (1.71-5.66)	**3.08 (1.69-5.60)**
**Smoking**					
No	34 (17.5)	1.0		1.0	
yes	160 (82.5)	1.34 (0.93-1.93)		1.17(0.71-1.90)	

[#] PR: Prevalence ratio; ** Trichomonas, Gonorrhea *
All significant variables were adjusted for age, sociodemographic and all other sexual behaviors.

For peer review only

For peer review only

For peer review only - http://bmjopen.bmj.com/site/about/guidelines.xhtml

Figure 1: Prevalence of any HPV and HPV by HIV status and sexual orientation

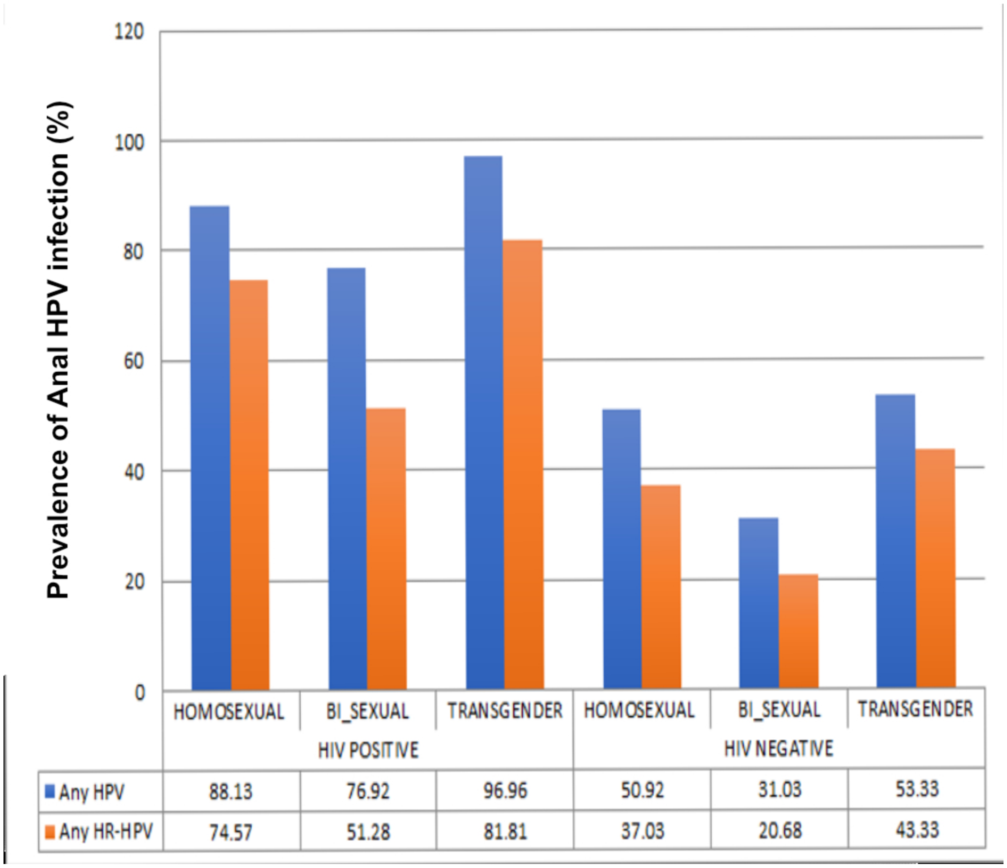

Figure1: Prevalence of any HPV and HR-HPV by HIV status and sexual orientation

RESEARCH CHECKLIST

Anal human papillomavirus infection among men who have sex with men and transgender women in Pakistan: Findings from a cross sectional study

Muslima Ejaz, MD MSc[1,2] Sören Andersson MD, PhD[3], Salma Batool PhD[4], Tazeen Saeed Ali PhD[5], Anna Mia Ekström MD MPH PhD[1]

	PAGE NUMBER
Title	1
Abstract	2
**Introduction:**	
Background/rationale	4
Objective	5
**METHODS**	
Study Design	5
Setting	5
Participants	5
Variables	6
Data Sources/measurement	6
Study size	6
Quantitative variables	6
Statistical methods	7
**RESULTS**	
Participants	7
Descriptive data	7
Outcome data	8
Main results	8
**DISCUSSION**	
Key results	9
Limitations	11
Generalisability	11
Interpretation	11
**Other information**	
Contribution	12
Acknowledgement	12
Funding	12
Conflict of interest	12
Presentation of this work	12

For peer review only
